# Arylboronic Acid Catalyzed *C*-Alkylation and Allylation Reactions Using Benzylic Alcohols

**DOI:** 10.1021/acs.orglett.0c02736

**Published:** 2020-09-22

**Authors:** Susana Estopiñá-Durán, Euan B. Mclean, Liam J. Donnelly, Bryony M. Hockin, James E. Taylor

**Affiliations:** † EaStCHEM, School of Chemistry, University of St Andrews, North Haugh, St. Andrews, KY16 9ST, U.K.; ‡ Department of Chemistry, University of Bath, Claverton Down, Bath, Somerset BA2 7AY, U.K.

## Abstract

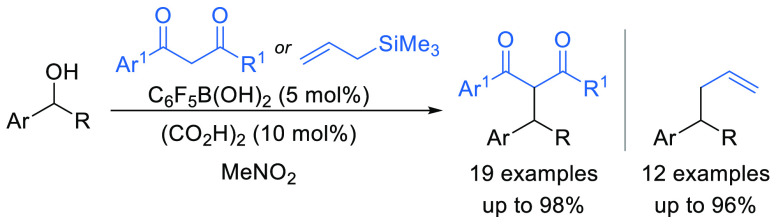

The arylboronic acid catalyzed dehydrative *C*-alkylation of 1,3-diketones and 1,3-ketoesters using secondary benzylic alcohols as the electrophile is reported, forming new C–C bonds (19 examples, up to 98% yield) with the release of water as the only byproduct. The process is also applicable to the allylation of benzylic alcohols using allyltrimethylsilane as the nucleophile (12 examples, up to 96% yield).

The formation of carbon–carbon bonds is central to the synthesis of organic molecules, with the alkylation of carbon-based pro-nucleophiles an important strategy within this area. Traditionally, alkylation reactions are performed using either alkyl halides or stoichiometrically activated alcohol derivatives as the electrophile. However, with the drive to develop more efficient and sustainable organic reactions,^1^ there has been increasing interest in catalytic methods for the direct use of alcohols as electrophiles in alkylation processes, releasing water as the only byproduct.^2^ Catalytic dehydrative substitutions can occur by a number of general mechanistic pathways including nucleophilic substitution, “borrowing hydrogen” via a redox reaction of primary or secondary alcohols,^3^ or addition to metal π-allyl complexes formed from allylic alcohols.^4^

Recently, arylboronic acids have gained increasing attention as catalysts that can activate hydroxyl groups toward both electrophilic and nucleophilic reactivity.^5^ Boronic acids are attractive as catalysts due to their wide availability, tractability, and generally low toxicity.^6^ Of particular relevance is the use of arylboronic acid catalysis for the activation of alcohols toward C–C bond formations through either complete or partial ionization of the C(sp^3^)–OH bond. In this regard, dehydrative Friedel–Crafts alkylation processes have been most widely explored to date (Scheme 1a).^7^ Seminal work by McCubbin^7a,7b^ and Hall^7c^ showed that electron-deficient arylboronic acids catalyze the Friedel–Crafts alkylation of electron-rich arenes and heteroarenes using either allylic or benzylic alcohols as the electrophile. The reaction scope has recently been extended to the use of electron-deficient arenes using 2,3,4,5-tetrafluorophenylboronic acid as the catalyst alongside perfluoropinacol as a cocatalyst.^7g^ Arylboronic acid catalysis can also be combined with enamine catalysis for the enantioselective α-alkylation of aldehydes using tertiary allylic alcohols.^8^ Other C–C bond formations promoted by the catalytic arylboronic acid activation of alcohols include dehydrative Nazarov cyclizations of divinyl alcohols,^9^ and [4 + 3] cycloadditions promoted by the ionization of indolyl alcohols.^10^

**Scheme 1:**
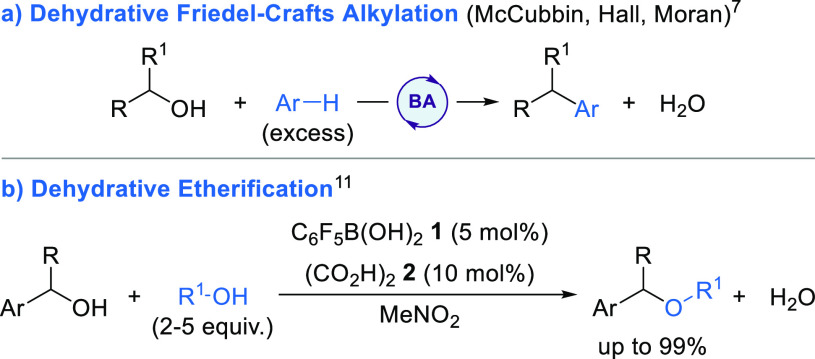
Boronic Acid Catalyzed Dehydrative Substitutions

We recently reported the use of catalytic pentafluorophenylboronic acid **1** alongside cocatalytic oxalic acid **2** for the activation of benzylic alcohols toward inter- and intramolecular dehydrative etherification reactions (Scheme 1b).^11^ Mechanistic investigations suggest that pentafluorophenylboronic acid **1** and oxalic acid **2** condense in situ to form a Brønsted acid catalyst that promotes S_N_1-type reactivity. We therefore questioned whether this system could be applied to the *C*-alkylation of 1,3-diketone derivatives and allylation reactions, which have not previously been explored using arylboronic acid catalysis. Various Brønsted acid catalysts have previously been reported for dehydrative C–C bond formations.^2,12^ However, the use of a tractable arylboronic acid would avoid the direct handling of strong acids and further expand the scope of reactions promoted by these readily available catalytic systems.

First, the use of enolizable 1,3-diketones as potential pro-nucleophiles was investigated with the reaction of benzhydrol with dibenzoylmethane. Reaction optimization showed that a combination of pentafluorophenylboronic acid **1** (5 mol %) and oxalic acid **2** (10 mol %) in MeNO_2_,^13^ a catalytic system first reported by Moran for a dehydrative Friedel–Crafts alkylation reaction,^7f^ gave the desired *C*-alkylation product **3** in 76% yield after 3 h at room temperature. In the absence of any catalyst or with pentafluorophenylboronic acid **1** alone, no reaction was observed, while using only oxalic acid **2** (10 mol %) gave 5% conversion into **3** over 3 h.^13^ The reaction scope was first investigated through variation of the 1,3-diketone component (Scheme 2). Symmetrical diketones bearing both electron-donating and electron-withdrawing substituents were tolerated under the standard reaction conditions, forming products **4** and **5** in good yields. Heterocycle containing acyl benzothiazoles and acyl benzoxazoles were also competent pro-nucleophiles, forming products **6** and **7** after extended 48 h reaction times at 90 °C, although the analogous acyl benzimidazole was unreactive under these conditions. The use of a cyclic 1,3-diketone was also possible, forming product **8** bearing a new quaternary carbon center in an excellent 93% yield. In contrast, the reaction of benzhydrol with 1,3-cyclohexanedione gave selective *O*-alkylation into the corresponding β-keto enol ether.^13,14^ Attempts to extend the scope to alternative enolizable ketones such as 2-phenylacetophenone or benzoylacetonitrile were unsuccessful, with only starting materials returned at room temperature. Using dibenzoylmethane (2 equiv) as standard, the use of various secondary benzylic alcohols as the electrophilic component was trialed. 1-Arylethanol derivatives bearing either neutral or electron-donating substituents were well tolerated, forming products **9**–**11** in excellent yields. The synthetic potential was demonstrated by performing the reaction on gram scale (4 mmol of alcohol) to give 1.25 g of **9** in 93% yield. Halogen substitution on the aryl ring was also possible with 4-fluoro- and 4-bromophenyl ethanol reacting to give **12** and **13** in 90% and 71% yield, respectively. Altering the substitution pattern affected the reactivity, with 1-(2-bromo- and 1-(3-bromophenyl)ethanol giving products **14** and **15** in slightly reduced yields. The presence of an alkyne on the reacting carbinol center was well tolerated, giving **16** in 98% yield. Limitations included the use of a sterically demanding secondary and tertiary alcohols, which are unreactive, while primary benzylic alcohols preferentially form the symmetric ether product.^13^

**Scheme 2:**
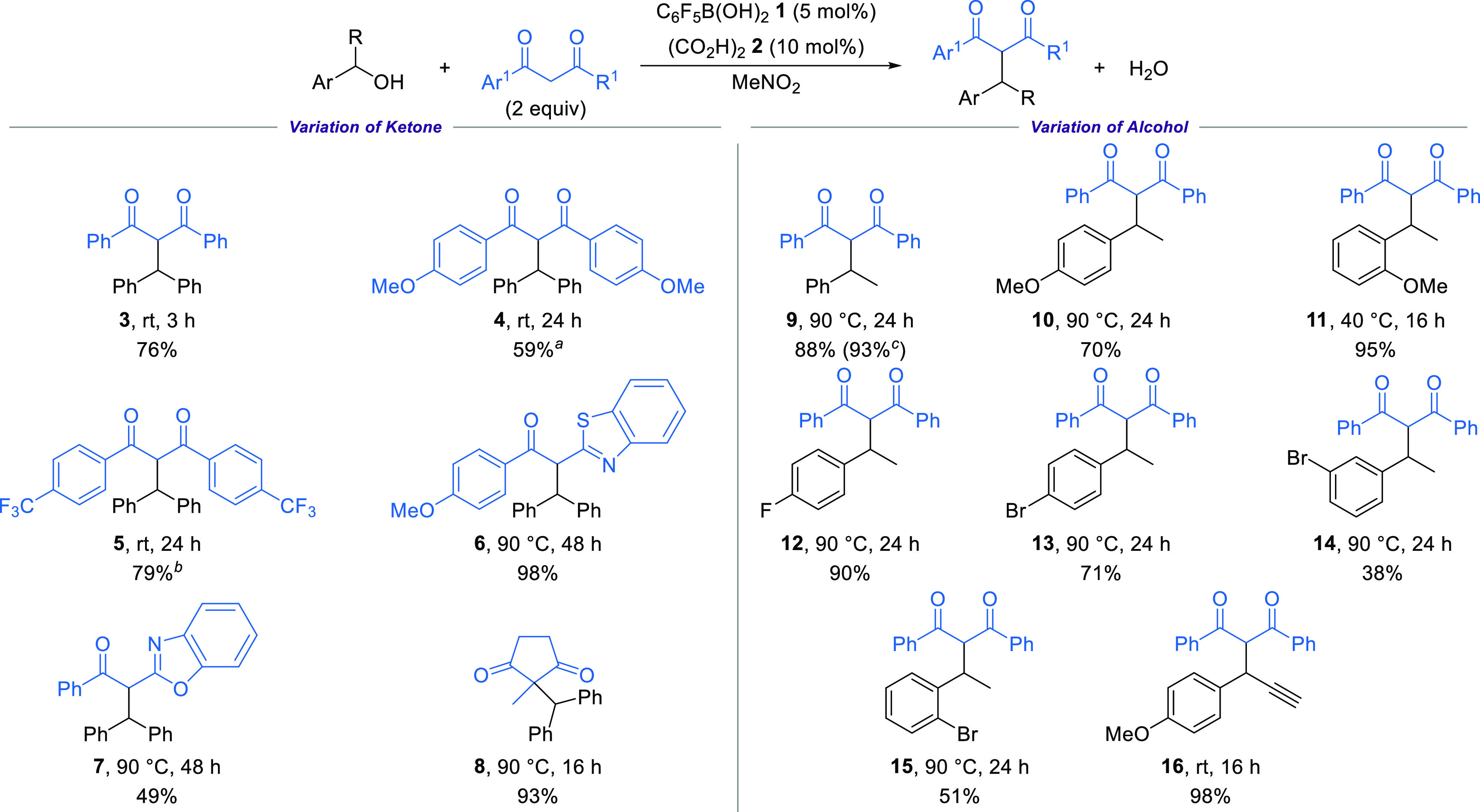
Dehydrative Alkylation of 1,3-Diektone Derivatives Using 1,3-diketone (5 equiv). Using benzhydrol (2 equiv) and 1,3-diketone (1 equiv). Reaction performed on a 4 mmol scale.

The use of 1,3-ketoesters as pro-nucleophiles was possible under the standard conditions (Scheme 3). For example, reacting ethyl benzoylacetate **17** with benzhydrol (2 equiv) gave product **18** in an excellent 97% yield after heating at 90 °C overnight. In this case, an excess of the alcohol was used to aid purification, with the symmetrical ether of benzhydrol formed as a side product. The use of 1-arylethanol derivatives bearing either electron-donating or halogen substituents as the electrophile gave *C*-alkylation products **19**–**22** in generally good yield as a mixture of diastereoisomers. Resubjecting an isolated sample of diastereomerically enriched product **21** (63:37 dr) to the reaction conditions led to equilibration of the diastereoisomers into the observed 53:47 dr, suggesting formation of a thermodynamic mixture. The product epimerization presumably occurs via catalyst-promoted enolization and protonation of the 1,3-ketoester stereocenter. The *C*-alkylation of 1,3-ketoesters could also be performed on gram scale (3.2 mmol of alcohol), giving 0.97 g of product **20** in 97% yield.

**Scheme 3:**
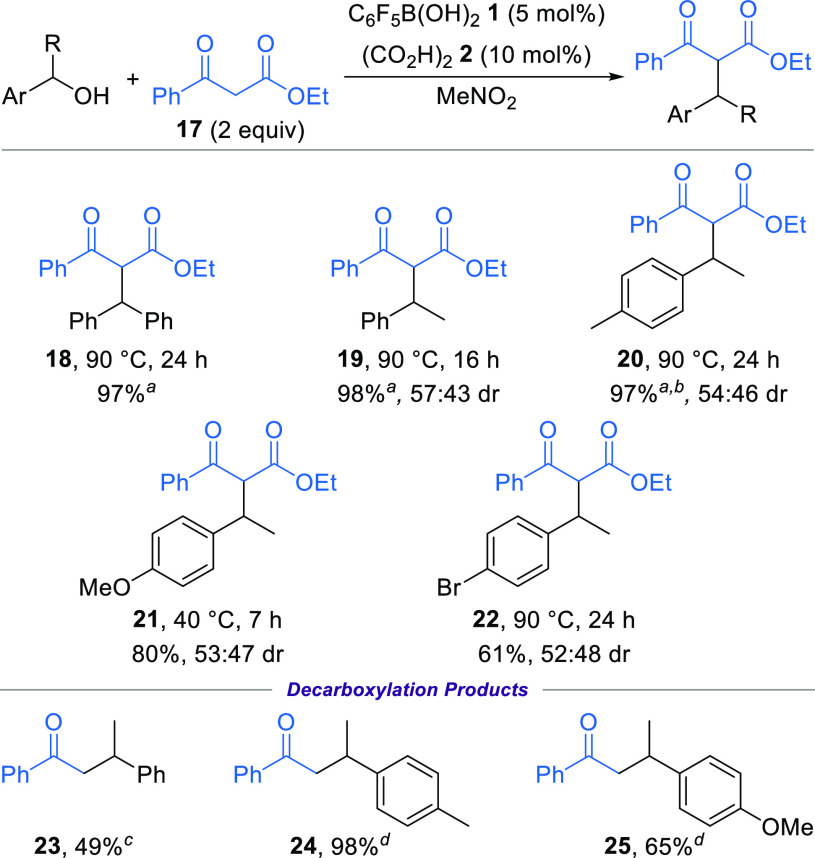
Use of 1,3-Ketoesters as Pro-nucleophiles Using alcohol (2 equiv) and ethyl benzoylacetate **17** (1 equiv). Reaction performed on a 3.2 mmol scale. KOH, MeOH, 70 °C. NaOH, EtOH, 80 °C

Furthermore, the isolated diastereomeric mixtures of products **19**–**21** could be derivatized into the corresponding β-aryl ketones **23**–**25** through decarboxylation under basic conditions.

Next, we sought to extend the *C*-alkylation protocol to a catalytic Hosomi–Sakurai process using allyl silanes as the nucleophile.^15^ Initial investigations reacting benzhydrol as the electrophile with allyltrimethylsilane **26** (2 equiv) using pentafluorophenylboronic acid **1** (5 mol %) and oxalic acid **2** (10 mol %) exclusively gave the symmetrical ether at room temperature in nitromethane. However, increasing the temperature to 90 °C gave allylation product **27** in excellent 96% yield (Scheme 4), with no formation of the unwanted symmetrical ether. Various secondary alcohols were trialed under the standard catalytic conditions. Electron-rich and halogen substituted 1-arylethanol derivatives were suitable electrophiles, forming allylation products **28**–**33** in moderate to good yields. In all cases, complete conversion into the allylation product was observed, but the nonpolar nature of the products resulted in loss of material during purification by chromatography accounting for some of the moderate yields. Unsubstituted 1-phenylethanol derivatives were not reactive, returning either starting materials or the corresponding symmetrical ether byproduct under all conditions tested.^13^ In contrast to the reactivity observed with 1,3-diketone nucleophiles, a secondary alcohol bearing a bulky *tert*-butyl substituent worked well, forming product **34** in 86% yield. Alkynyl and extended alkenyl substituents were also well tolerated, with products **35** and **36** formed in 80% and 90% yield, respectively. The catalytic allylation of an electron-rich tertiary alcohol was also possible, forming product **37** with a new quaternary carbon center in 68% yield. The electron-donating methoxy substituent on the aryl ring was essential for reactivity, with the analogous unsubstituted phenyl substrate returned unreacted under the same conditions. Cinnamyl trimethylsilane could also be used as a nucleophile, giving **38** in 77% yield as a 64:36 mixture of diastereoisomers at room temperature.

**Scheme 4:**
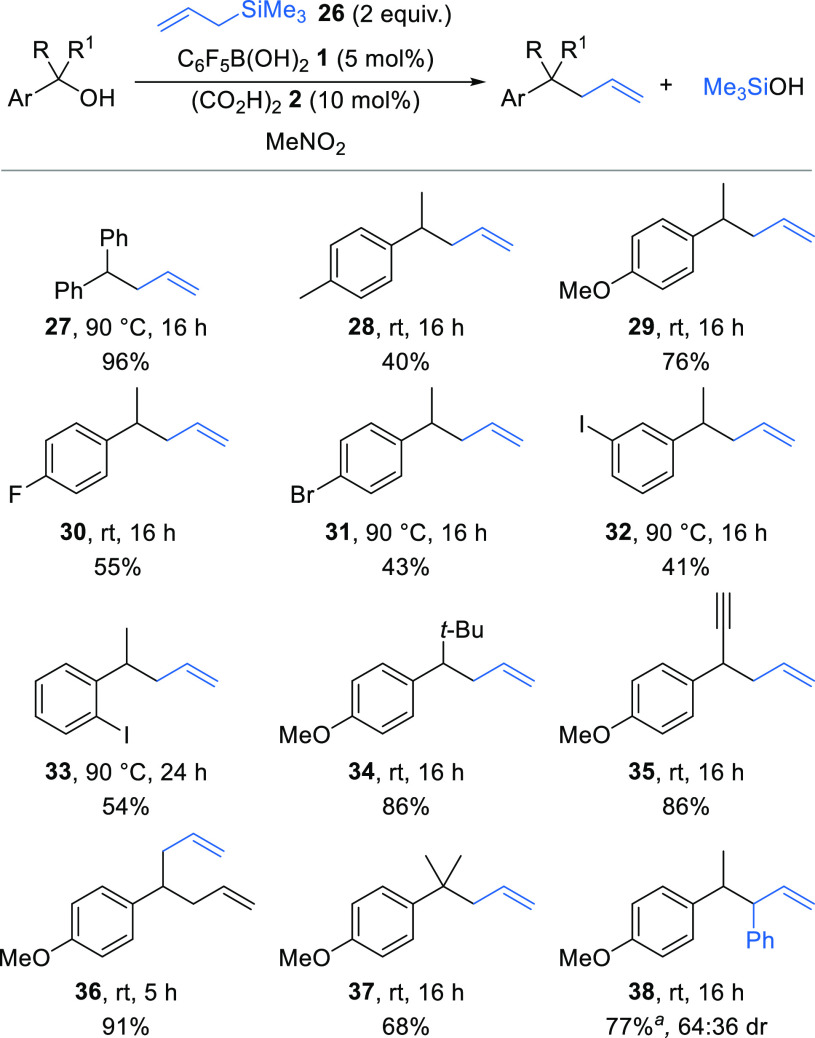
Allylation of Benzylic Alcohols Using cinnamyl trimethylsilane (2 equiv).

We have previously shown that pentafluorophenylboronic acid **1** and oxalic acid **2** condense in situ to form hydrated boronate ester **39**, which acts as a strong Brønsted acid to promote S_N_1 type reactivity through formation of an intermediate benzylic carbocation from the secondary alcohol.^11^ This is consistent with the literature on related arylboronic acid catalyzed reactions and accounts for the higher reactivity observed for electron-rich secondary benzylic alcohols in the substrate scope. A possible catalytic cycle for the dehydrative *C*-alkylation process is outlined in Scheme 5. In solution, pentafluorophenylboronic acid **1** and oxalic acid **2** are in dynamic equilibrium with hydrated boronate **39**,^16^ which is likely to act as a Brønsted acid to protonate the secondary benzylic alcohol. This is consistent with recent work by Moran and co-workers, who found that various arylboronic acid promoted alcohol activation processes are likely to proceed via either a Brønsted acid or H-bond activation mode, as opposed to Lewis acid or covalent catalysis.^17^ Dissociation of ion pair **40** forms benzylic carbocation **41**, which can undergo nucleophilic addition from the enol tautomer of either the 1,3-diketone derivatives or 1,3-ketoesters to form the *C*-alkylation products and release water as the only byproduct. An analogous mechanism is plausible using allyltrimethylsilane **26** as the nucleophile reacting with carbocation **41**, with trimethylsilanol released as the byproduct in this case.^18^

**Scheme 5:**
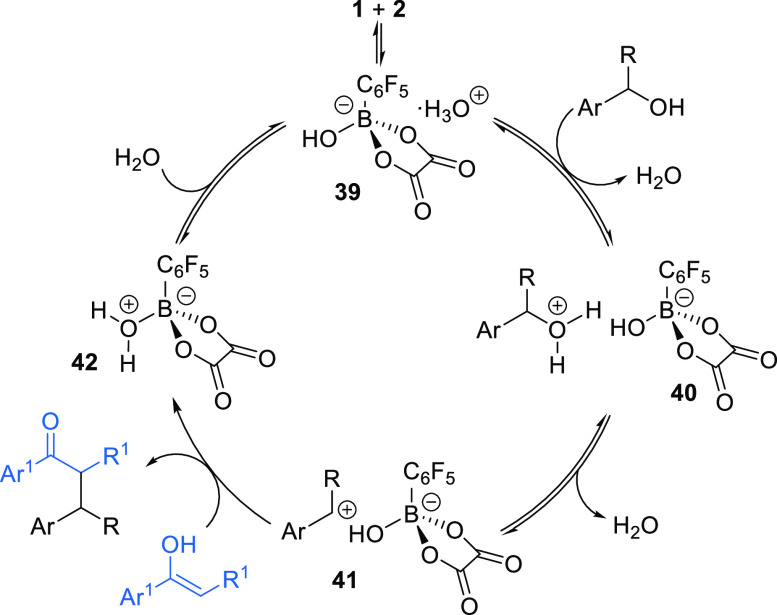
Possible Catalytic Cycle

In conclusion, arylboronic acid catalysis can be used for the dehydrative *C*-alkylation of various carbon nucleophiles using secondary benzylic alcohols as the electrophile. A range of 1,3-diketones and 1,3-ketoesters can be used as pro-nucleophiles toward secondary benzylic alcohols activated by a combination of pentafluorphenylboronic acid **1** (5 mol %) and oxalic acid **2** (10 mol %) to form *C*-alkylation products in good yields, with water formed as the only byproduct. The catalytic system is also compatible with allyltrimethylsilane **26** as the nucleophile, promoting the direct allylation of various benzylic alcohols. Further studies into the applicability of arylboronic acid catalysis toward dehydrative substitution reactions are ongoing in our laboratory.^19^

## Supporting Information Available

Experimental details, compound characterization, and NMR spectra for novel compounds (PDF)
