# Supplementary material for: Arylboronic Acid Catalyzed C-Alkylation and Allylation Reactions Using Benzylic Alcohols
Source: Org Lett. 2020 Sep 22;22(19):7547–51. doi: 10.1021/acs.orglett.0c02736 (PMC8155392; doi:10.1021/acs.orglett.0c02736)
Supplement: Supplementary file 1 — ol0c02736_si_001.pdf [file ol0c02736_si_001.pdf]

Arylboronic Acid-Catalyzed *C*-Alkylation and Allylation Reactions Using Benzylic Alcohols

Susana Estopiñá-Durán,[†,‡] Euan B. Mclean,[†] Liam J. Donnelly,[†] Bryony M. Hockin,[†] James E. Taylor*[,‡]

[†]*EaStCHEM, School of Chemistry, University of St Andrews, North Haugh, St Andrews, KY16 9ST, U.K.*

[‡]*Department of Chemistry, University of Bath, Claverton Down, Bath, Somerset, BA2 7AY, U.K.*

General Information S2

Reaction Optimization S4

Control Experiments S6

Ketoester Epimerization S6
Regioselectivity S6
Symmetrical Ether Intermediate S7

Additional Substrate Scope S9

Cyclic 1,3-Diketones S9
1,3-Ketoesters S10
Allylation S10

Synthesis of Starting Materials S11

Benzylic Alcohols S11
1,3-Diketones S17
Silanes S18

Dehydrative Alkylation of 1,3-Diketone Derivatives S19

Dehydrative Alkylation of 1,3-Ketoesters S28

Decarboxylation of 1,3-Ketoesters S32

Dehydrative Allylation S34

Control Reactions S40

NMR Spectra S42

References S95

General Information

Reactions involving moisture sensitive reagents were carried out in flame-dried glassware under an inert atmosphere (N_2) using standard vacuum line techniques. Anhydrous solvents (Et_2O, CH_2Cl_2, and THF) were obtained after passing through an alumina column (Mbraun SPS-800). Petrol is defined as petroleum ether 40–60 °C. All other solvents and commercial reagents were used as received without further purification unless otherwise stated.

Room temperature (rt) refers to 20–25 °C. Temperatures of 0 °C were obtained using an ice/water bath. Reaction involving heating were performed using DrySyn blocks and a contact thermocouple.

Analytical thin layer chromatography was performed on pre-coated aluminium plates (Kieselgel 60 F254 silica) and visualisation was achieved using ultraviolet light (254 nm) and/or staining with either aqueous $KMnO_4$ solution or ethanolic phosphomolybdic acid followed by heating. Manual column chromatography was performed in glass columns fitted with porosity 3 sintered discs over Kieselgel 60 silica using the solvent system stated.

Melting points were recorded on either an Electrothermal 9100 melting point apparatus or an Stanford Research Systems OptiMelt automated capillary melting point apparatus in open capillary tubes, (dec) refers to decomposition.

Infrared spectra were recorded on either a Shimadzu IRAffinity-1 Fourier transform IR spectrophotometer fitted with a Specac Quest ATR accessory (diamond puck) or on a Perkin-Elmer PerkinElmer Spectrum 100 ATR-FTIR spectrometer. Spectra were recorded of either thin films or solids, with characteristic absorption wavenumbers (ν_{max}) reported in cm^{-1}.

^1H, $^{13}C\{^1H\}$, and $^{19}F\{^1H\}$ NMR spectra were acquired on either a Bruker AV300 (^1H 300 MHz; $^{13}C\{^1H\}$ 75 MHz; $^{19}F\{^1H\}$ 282 MHz), a Bruker AV400 (^1H 400 MHz; $^{13}C\{^1H\}$ 101 MHz; $^{19}F\{^1H\}$ 376 MHz), a Bruker AVII 400 (^1H 400 MHz; $^{13}C\{^1H\}$ 101 MHz; $^{19}F\{^1H\}$ 376 MHz), a Bruker Neo 400 (^1H 400 MHz; $^{13}C\{^1H\}$ 101 MHz; $^{19}F\{^1H\}$ 376 MHz), a Bruker AVIII-HD 500 (^1H 500 MHz, $^{13}C\{^1H\}$ 126 MHz, $^{19}F\{^1H\}$ 470 MHz, ^{11}B 160 MHz), a Bruker AVIII 500 (^1H 500 MHz, $^{13}C\{^1H\}$ 126 MHz, $^{19}F\{^1H\}$ 470 MHz, ^{11}B 160 MHz), or an Agilent ProPulse 500 (^1H 500 MHz, $^{13}C\{^1H\}$ 126 MHz, $^{19}F\{^1H\}$ 470 MHz) in the deuterated solvent stated. All chemical shifts are quoted in parts per million (ppm) relative to the residual solvent peak. All coupling constants, *J*, are quoted in Hz. Multiplicities are indicated as s (singlet), d (doublet), t (triplet), q (quartet), m (multiplet), and multiples thereof. The abbreviation Ar denotes aromatic and app denotes apparent. NMR peak assignments were confirmed using 2D ^1H

correlated spectroscopy (COSY), 2D $^{1}H–^{13}C$ heteronuclear multiple-bond correlation spectroscopy (HMBC), and 2D $^{1}H–^{13}C$ heteronuclear single quantum coherence (HSQC) where necessary.

Mass spectrometry (*m/z*) data were acquired by either electrospray ionisation (ESI), chemical ionisation (CI), electron impact (EI), atmospheric solids analysis probe (ASAP), atmospheric pressure chemical ionization (APCI) or nanospray ionisation (NSI) at either the University of St Andrews Mass Spectrometry Facility ([A] quoted), the EPSRC UK National Mass Spectrometry Facility at Swansea University ($[A]^{+}$ or $[A]^{-}$ quoted), or at the University of Bath ($[A]^{+}$ or $[A]^{-}$ quoted) using either a TOF or QTof mass analyzer.

Reaction Optimization

Table S1. Catalyst and Ligand Screening[a]

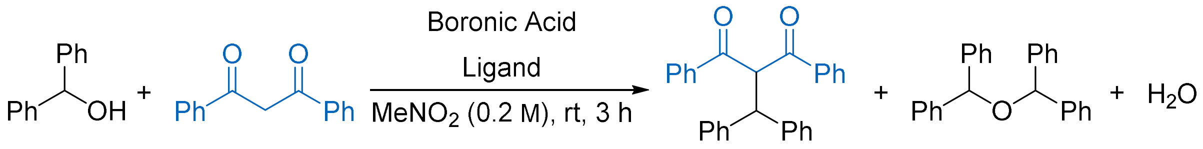

S1 **S2** (2 equiv.) **3** **S3**

Boronic Acids

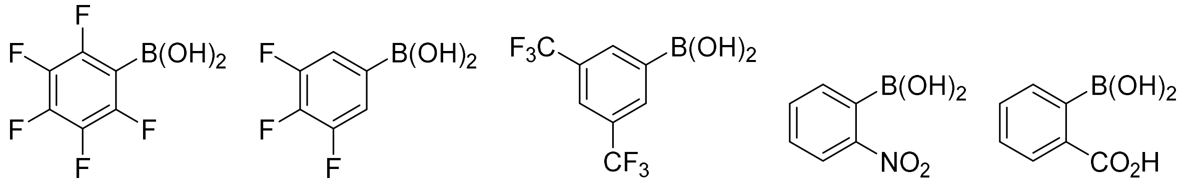

1 **S4** **S5** **S6** **S7**

Ligands

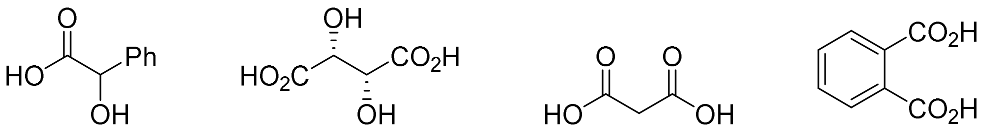

2 **S8** **S9** **S10**

Entry	Boronic acid (mol%)	Ligand (mol%)	Conversion[b]	**3**:**S3**[b]
1	–	–	0%	N/A
2	–	**2** (10)	5%	100:0
3	**1** (5)	**2** (10)	100%	99:1
4	**S4** (5)	**2** (10)	77%	91:9
5	**S5** (5)	**2** (10)	83%	90:10
6	**S6** (5)	**2** (10)	65%	91:9
7	**S7** (5)	**2** (10)	98%	94:6
8	**1** (5)	–	<1%	N/A
9	**1** (5)	**S8** (10)	<5%	N/A
10	**1** (5)	**S9** (10)	<5%	N/A
11	**1** (5)	**S10** (10)	<5%	N/A
12	**1** (5)	**2** (2.5)	56%	89:11
13	**1** (5)	**2** (5)	75%	90:10
14	**1** (2)	**2** (4)	60%	88:12

[a]Reactions performed on 0.2 mmol scale. [b]Determined by ^{1}H NMR analysis.

Table S2. Solvent, Concentration and Equivalents Screening[a]

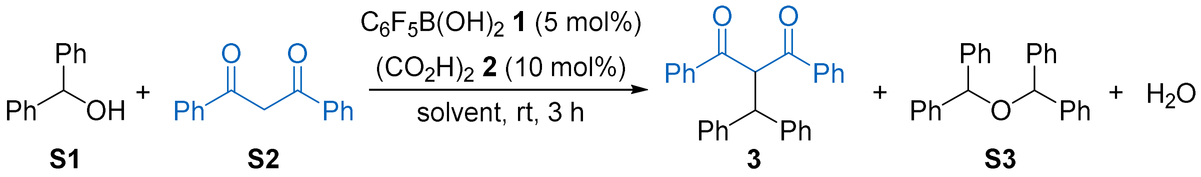

Entry	**S2** (equiv.)	Solvent (M)	Conversion[b]	**3**:**S3**[b]
1	2	$MeNO_2$ (0.2)	100%	99:1
2	2	HFIP[c] (0.2)	100%	>99:1
3	2	MeCN (0.2)	34%	62:38
4	2	PhMe (0.2)	<5%	N/A
5	2	CH_2Cl_2 (0.2)	7%	57:43
6	2	$MeNO_2$ (0.5)	99%	95:5
7	2	$MeNO_2$ (0.1)	100%	>99:1
8	2	$MeNO_2$ (0.05)	100% (76%)[d]	>99:1
9	1.5	$MeNO_2$ (0.05)	100%	>99:1
10	1.2	$MeNO_2$ (0.05)	100%	>99:1
11	1	$MeNO_2$ (0.05)	100%	>99:1

[a]Entries 1-8 performed on 0.1 mmol scale, entries 9-11 performed on 0.2 mmol scale. [b]Determined by ^{1}H NMR analysis. [c]1,1,1,3,3,3-Hexafluoro-2-propanol. [d]Isolated yield.

Control Experiments

Ketoester Epimerization

Reacting an isolated sample of **21** (63:37 dr) under the standard conditions for 4 h at rt resulted in a change in the diastereomeric ratio (to 53:47 dr), showing that the catalyst may promote epimerization of the products, leading to formation of a thermodynamic mixture of diastereoisomers.

Scheme S1. Ketoester Epimerization

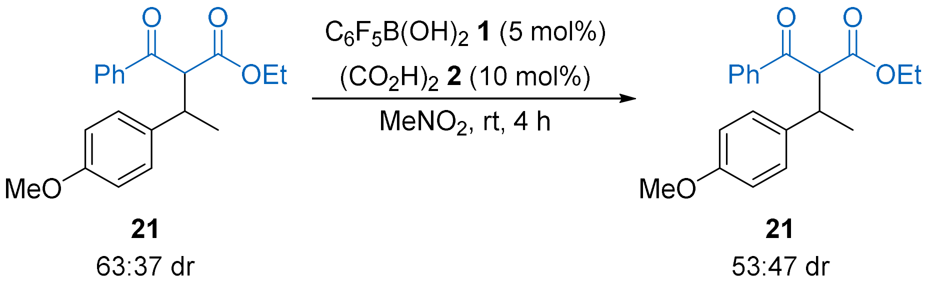

Regioselectivity

Reacting dibenzoylmethane (2 equiv.) with either α-vinylbenzyl alcohol **S11** (Scheme S2a) or isomeric cinnamyl alcohol **S12** (Scheme S2b) under the standard conditions led to a compariable yield and mixture of regioisomeric products **S13** and **S14** in both cases, with the linear *C*-alkylation product favoured.

Scheme S2. *C*-Alkylation Regioselectivity

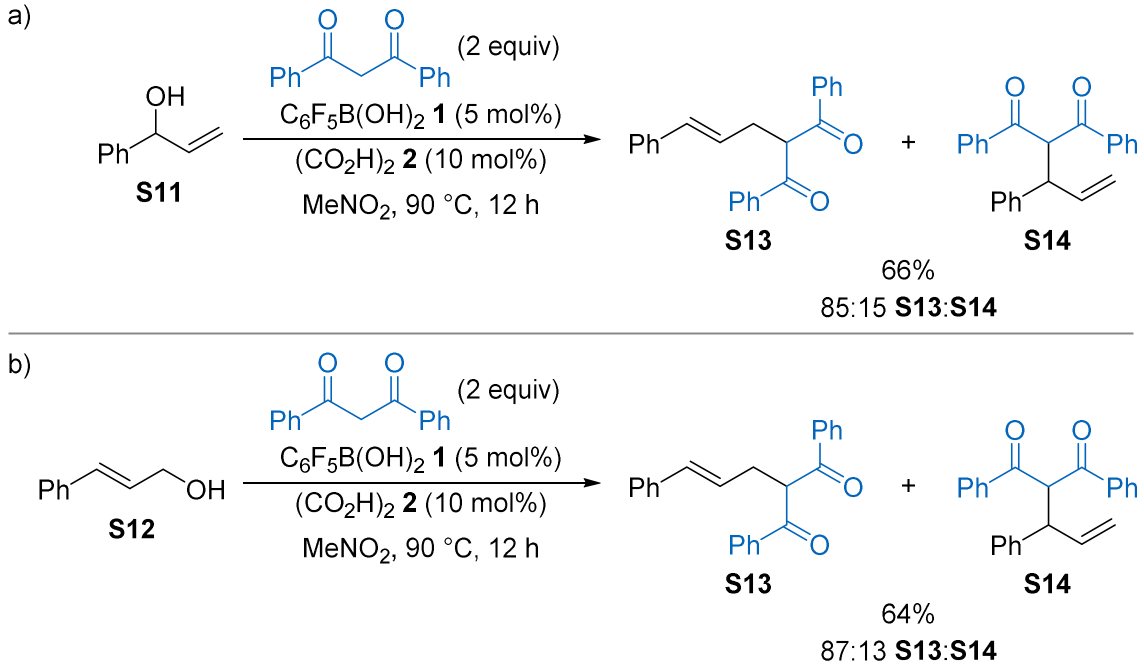

Reacting allyltrimethylsilane (2 equiv.) with either α-vinylbenzyl alcohol **39** (Scheme S3a) or isomeric cinnamyl alcohol **40** (Scheme S3b) under the standard conditions gave complete selectivity for linear product **S15** in both cases in compariable yield. These results support the formation of a common cation intermediate leading to the same product distributions from isomeric starting materials.

Scheme S3. Allylation Regioselectivity

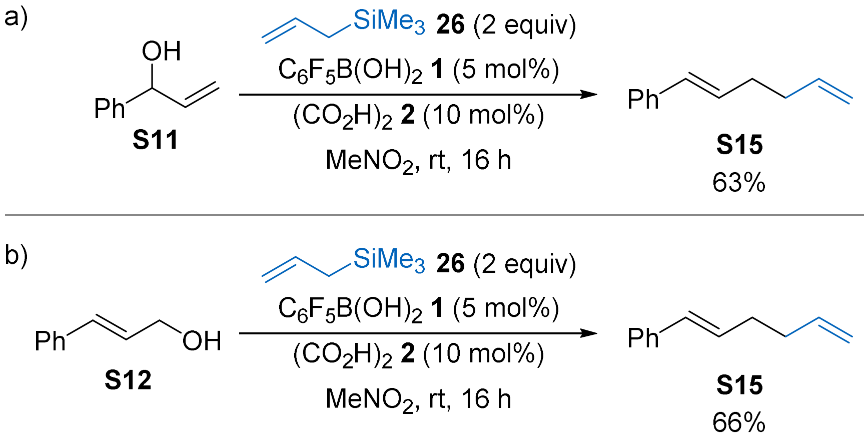

Symmetrical Ether Intermediate

Hall and co-workers have shown that symmetric ether formation was kinetically favored for a 2,3,4,5-tetrafluorophenylboronic acid (10 mol%) and perfluoropinacol (10 mol%) catalyzed Friedel-Crafts alkylation reaction using benzylic alcohols, with the ether subsequently being converted into the desired product.[1] Reaction monitoring showed that the symmetrical ether is likely to be an intermediate and a control experiment showed the ether was a competent starting material. To investigate this possibility for the reaction of 1,3-diketones, ether **S3** was reacted under the standard reaction conditions for 3 h at rt (Scheme S4). Analysis of the crude reaction mixture with ^{1}H NMR showed 58% conversion of ether **S3** into mainly *C*-alkylation product **3**, with a small amount of benzyhydrol **S1** also observed.

Scheme S4. Symmetrical Ether as Starting Material in *C*-Alkylation

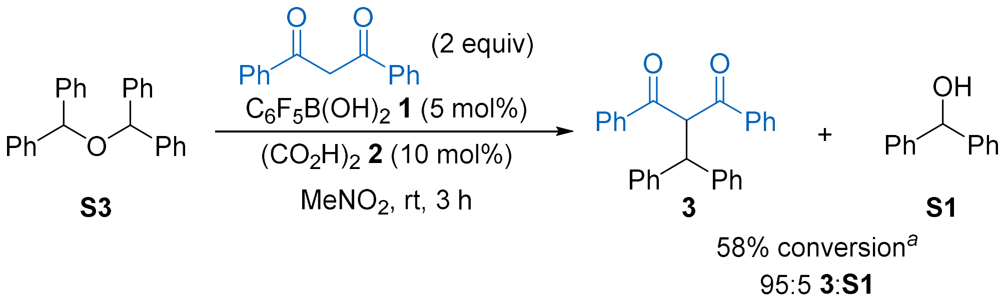

The same experimenmt was performed using ether **S3** and allyltrimethylsilane **26** (2 equiv.) under the standard reaction conditions in $MeNO_2$ at 90 °C for 16 h (Scheme S1). Again, ^1H NMR analysis showed ether **S3** was a competent precursor, giving 74% conversion into product **27** (Scheme S5a). However, an analogous experiment using symmetric ether **S16** (1:1 dr) showed no reaction by ^1H NMR spectroscopy after 16 h (Scheme S5b), suggesting that the role of symmetric ethers as potential intermediates is substrate specific.

Scheme S5. Symmetrical Ethers as Starting Material in Allylation

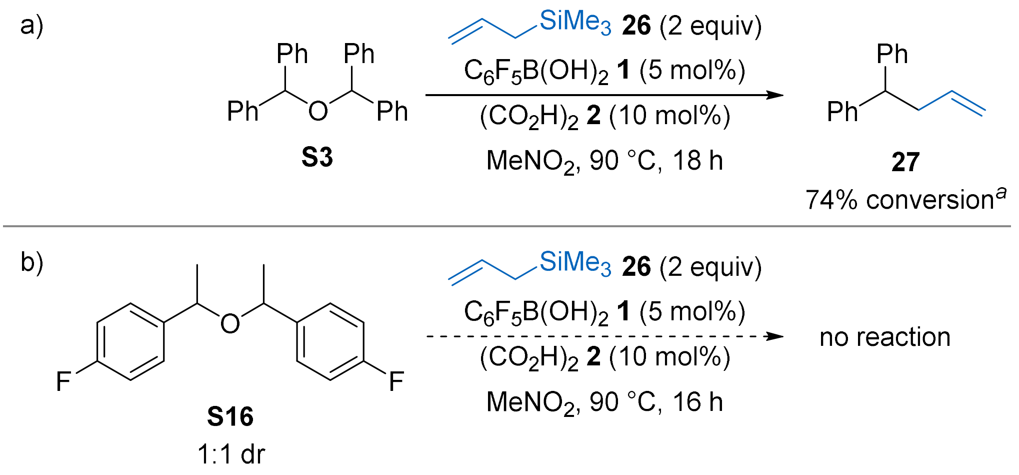

Additional Substrate Scope

Cyclic 1,3-Diketones

The reaction of benzhydrol with 1,3-cyclohexanedione gave selective *O*-alkylation under the standard reaction conditions, forming β-keto enol ether **S17** in 73% yield (Scheme S6). This process was specific to benzyhdrol, with a range of other secondary benzylic alcohols returning only starting materials under the same conditions.

Scheme S6. Reaction with 1,3-Cyclohexanedione

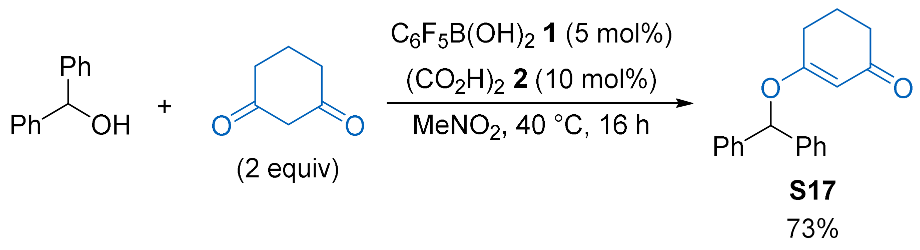

Unsuccessful substrates that were evaluated under the standard reaction conditions for the dehydrative alkylation of 1,3-diketones in Scheme S7 below.

Scheme S7. Addition Substrate Evaluation with 1,3-Diketones

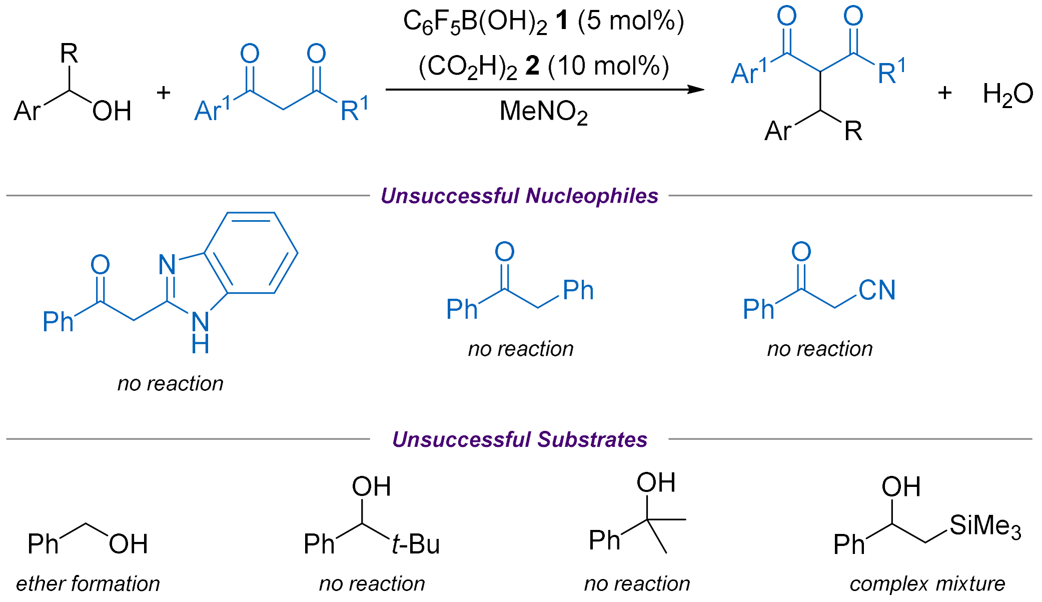

1,3-Ketoesters

Unsuccessful substrates that were evaluated under the standard reaction conditions for the dehydrative alkylation of ethyl benzoylacetate are show in Scheme S8 below.

Scheme S8. Addition Substrate Evaluation with 1,3-Ketoesters

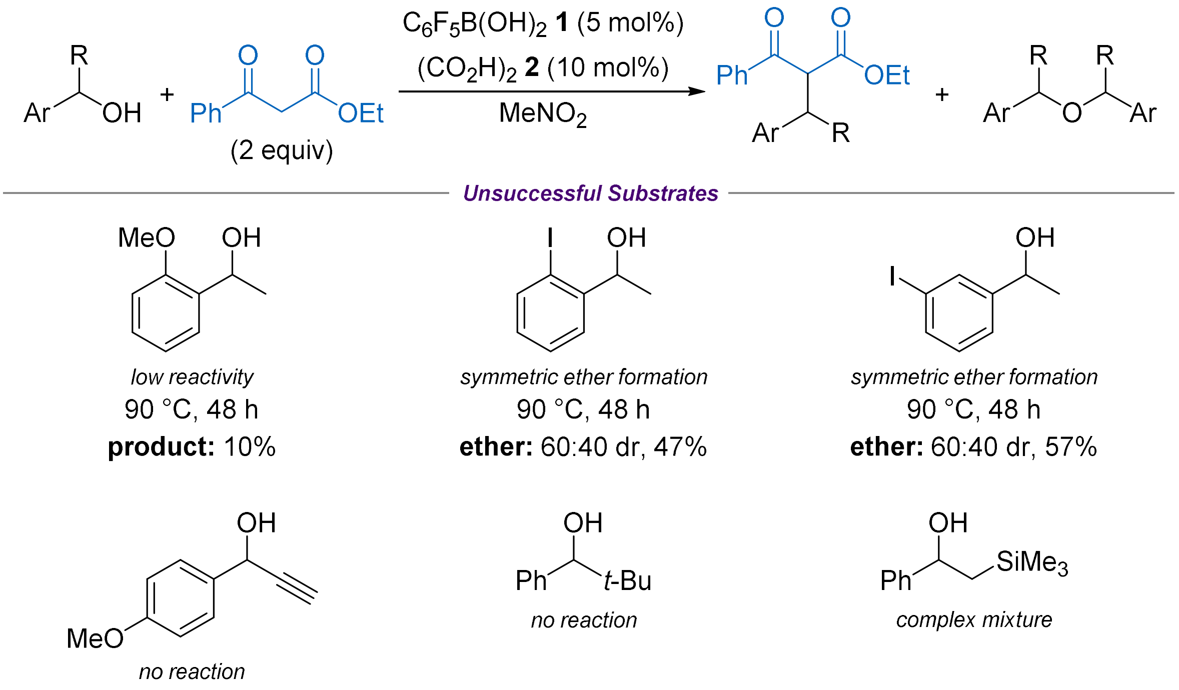

Allylation

Unsuccessful substrates that were evaluated under the standard reaction conditions for the allylation of benzylic alcohols are show in Scheme S9 below.

Scheme S9. Addition Substrate Evaluation for the Allylation of Benzylic Alcohols

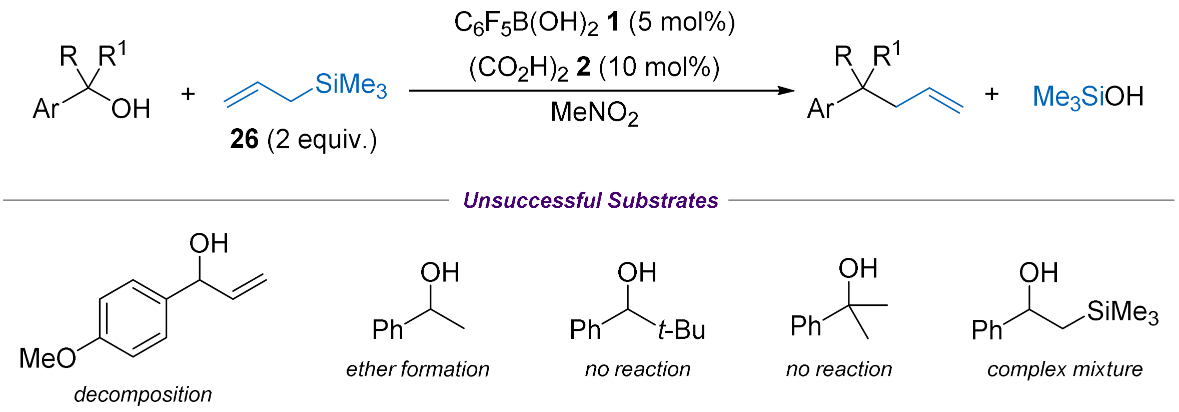

Synthesis of Starting Materials

Benzylic Alcohols

All other benzylic aclohols were used as received from commercial sources.

General Procedure A: Grignard addition

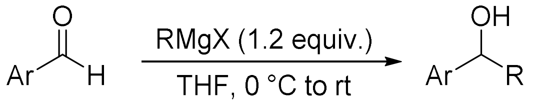

The required Grignard reagent (1.2 equiv.) was added dropwise to a solution of the required aldehyde (1.0 equiv.) in anhydrous THF (0.35 M) at 0 ºC under an atmosphere of N_2. The mixture was stirred at 0 ºC for 30 min and then at rt for 6 h. The reaction was quenched with aq. NH_4Cl and the organic layer was separated. The aqueous layer was extracted with EtOAc (3×20 mL). The combined organic layers were washed with brine (3×10 mL), dried over $MgSO_4$, filtered, and concentrated under reduced pressure. The residual oil was either purified by silica-gel column chromatography (petrol/EtOAc) or used without further purification.

General Procedure B: Reduction

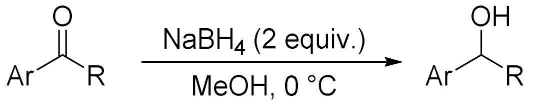

Sodium borohydride (2.0 equiv.) was added to a solution of the required aldehyde (1.0 equiv.) dissolved in methanol (0.4 M) at 0 °C. The mixture was stirred at 0 °C until complete by TLC analysis. The mixture was concentrated under reduced pressure before being diluted with CH_2Cl_2, washed with water, dried over Na_2SO_4, filtered, and concentrated under reduced pressure. to afford the product, which was used without further purification.

1-Phenylethan-1-ol S18

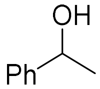

Following **General Procedure A**, benzaldehyde (1.04 g, 9.8 mmol) and MeMgBr (3.0 M in THF, 3.93 mL, 11.8 mmol) were reacted in THF (24 mL). The reaction did not require further purification to give title compound **S18** (1.20 g, 99%) as an orange oil, with spectroscopic data in accordance with the literature.[2] ^{1}H NMR (400 MHz, $CDCl_3$) δ_H: 7.46–7.35 (m, 3H, ArC(3,4,5)*H*), 7.34–7.25 (m, 2H, ArC(2,6)*H*), 4.90 (q, *J* = 6.5 Hz, 1H, OC*H*), 2.04 (s, 1H, O*H*), 1.50 (d, *J* = 6.5 Hz, 3H, CHC*H*$_3$).

1-(4-Methoxyphenyl)ethan-1-ol S19

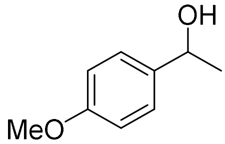

Following **General Procedure A**, 4-methoxybenzaldehyde (1.33 g, 9.8 mmol) and MeMgBr (3.0 M in THF, 3.93 mL, 11.8 mmol) were reacted in THF (24 mL). The crude product was purified by silica-gel column chromatography (petrol/EtOAc, 9:1 to 7:3, R_f 0.39 (8:2)) to give title compound **S19** (0.517 g, 35%) as colourless oil, with spectroscopic data in accordance with the literature.[2] ^{1}H NMR (400 MHz, $CDCl_3$) δ_H: 7.35–7.22 (m, 2H, ArC(2,6)*H*), 6.93–6.82 (m, 2H, ArC(3,5)*H*), 4.85 (q, *J* = 6.4 Hz, 1H, PhC*H*OH), 3.80 (s, 3H, OCH_3), 1.83 (s, 1H, O*H*), 1.47 (d, *J* = 6.4 Hz, 3H, CHCH_3).

1-(2-Methoxyphenyl)ethan-1-ol S20

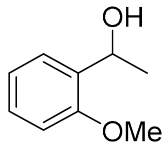

Following **General Procedure A**, 2-methoxybenzaldehyde (0.50 g, 3.7 mmol) and MeMgBr (3.0 M in Et_2O, 1.47 mL, 4.41 mmol) were reacted in Et_2O (15 mL). The reaction did not require further purification to give title compound **S20** (0.51 g, 89%) as an orange oil, with spectroscopic data in accordance with the literature.[2] ^{1}H NMR (400 MHz, $CDCl_3$) δ_H: 7.40–7.34 (m, 1H, Ar*H*), 7.32–7.25 (m, 1H, Ar*H*), 7.04–6.95 (m, 1H, Ar*H*), 6.95–6.87 (m, 1H, Ar*H*), 5.17–5.07 (m, 1H, C*H*CH_3), 3.90 (s, 3H, OCH_3), 2.66 (s, 1H, O*H*), 1.54 (d, *J* = 6.6 Hz, 3H, CHCH_3).

1-(4-Fluorophenyl)ethan-1-ol, S21

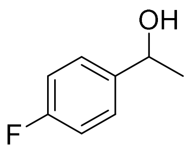

Following **General Procedure B**, 1-(4-fluorophenyl)ethan-1-one (2.0 mL, 16.5 mmol) and $NaBH_4$ (1.25 g, 33 mmol) were reacted in MeOH (83 mL). The crude product was purified by silica-gel column chromatography (petrol/EtOAc, 80:20 to 70/30, R_f: 0.31) to give title compound **S21** (1.94 g, 84%) as a colourless oil, with spectroscopic data in accordance with the literature.[2] H NMR (300 MHz, $CDCl_3$) δ_H: 7.41–7.28 (m, 2H, Ar(2,6)*H*), 7.10–6.97 (m, 2H, ArC(3,5)*H*), 4.89 (q, *J* = 6.4 Hz, 1H, C*H*CH_3), 1.48 (d, *J* = 6.5 Hz, 3H, CHCH_3).

1-(4-Bromophenyl)ethan-1-ol S22

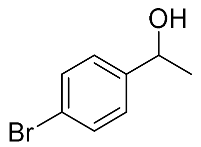

Following **General Procedure A**, 4-bromobenzaldehyde (1.81 g, 9.8 mmol) and MeMgBr (3.0 M in THF, 3.93 mL, 11.8 mmol) were reacted in THF (24 mL). The reaction did not require further purification to give title compound **S22** (1.95 g, 99%) as an orange oil, with spectroscopic data in accordance with the literature.[2] ^{1}H NMR (400 MHz, $CDCl_3$) δ_H: 7.52–7.42 (m, 2H, Ar*H*), 7.33–7.21 (m, 2H, Ar*H*), 4.86 (q, *J* = 6.5 Hz, 1H, C*H*CH_3), 1.98 (s, 1H, O*H*), 1.53–1.45 (m, 3H, CHCH_3).

1-(3-Bromophenyl)ethan-1-ol S23

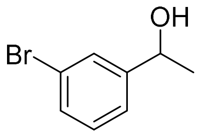

Following **General Procedure A**, 3-bromobenzaldehyde (1.81 g, 9.8 mmol) and MeMgBr (3.0 M in THF, 3.93 mL, 11.8 mmol) were reacted in THF (24 mL). The reaction did not require further purification to give title compound **S23** (1.92 g, 98%) as an orange oil, with spectroscopic data in accordance with the literature.[2] ^{1}H NMR (400 MHz, $CDCl_3$) δ_H: 7.57–7.47 (m, 1H, Ar*H*), 7.43–7.34 (m, 1H, Ar*H*), 7.31–7.23 (m, 1H, Ar*H*), 7.23–7.13 (m, 1H, Ar*H*), 4.84 (q, *J* = 6.5 Hz, 1H, C*H*CH_3), 2.15 (s, 1H, O*H*), 1.46 (d, *J* = 6.5 Hz, 3H, CHCH_3).

1-(2-Bromophenyl)ethan-1-ol S24

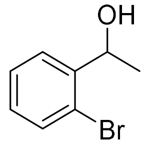

Following **General Procedure A**, 2-bromobenzaldehyde (1.81 g, 9.8 mmol) and MeMgBr (3.0 M in THF, 3.93 mL, 11.8 mmol) were reacted in THF (24 mL). The reaction did not require further purification to give title compound **S24** (1.94 g, 99%) as an orange oil, with spectroscopic data in accordance with the literature.[3] ^{1}H NMR (400 MHz, $CDCl_3$) δ_H: 7.63–7.57 (m, 1H, Ar*H*), 7.52–7.49 (m, 1H, Ar*H*), 7.39–7.31 (m, 1H, Ar*H*), 7.20–7.05 (m, 1H, Ar*H*), 5.33–5.15 (m, 1H, C*H*CH_3), 2.17 (s, 1H, O*H*), 1.49–1.45 (m, 3H, CHCH_3).

1-(4-Methoxyphenyl)prop-2-yn-1-ol, S25

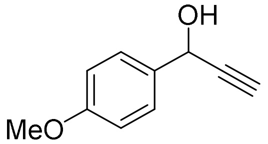

Following **General Procedure A**, 4-methoxybenzaldehyde (1.2 mL, 9.8 mmol) and ethynylmagnesium chloride (0.5 M in THF/Toluene, 24 mL, 11.8 mmol) were reacted in THF (24 mL). The crude product was purified by silica-gel column chromatography (petrol/EtOAc, 90:10 to 70:30, R_f: 0.33) to give title compound **S25** (1.21 g, 76%) as yellow oil, with spectroscopic data in accordance with the literature.[4] ^{1}H NMR (300 MHz, $CDCl_3$) δ_H: 7.58–7.42 (m, 2H, ArC(2,6)*H*), 6.99–6.82 (m, 2H, ArC(3,5)*H*), 5.43 (q, *J* = 6.2 Hz, 1H, ArC*H*OH), 3.82 (s, 3H, OCH_3), 2.67 (d, *J* = 2.3 Hz, 1H, CC*H*).

2,2-Dimethyl-1-phenylpropan-1-ol S26

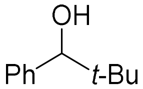

Following **General Procedure A**, benzaldehyde (1.04 mL, 9.8 mmol) and *t*-BuMgCl (1.0 M in THF, 11.8 mL, 11.8 mmol) were reacted in THF (15 mL). The crude product was purified by silica-gel column chromatography (petrol/EtOAc, 9.5:0.5 to 90:10, R_f 0.51) to give title compound **S26** (0.361 g, 22%) as a yellow oil, with spectroscopic data in accordance with the literature.[5] ^{1}H NMR (400 MHz, $CDCl_3$) δ_H: 7.34–7.22 (m, 5H, Ar*H*), 4.38 (s, 1H, PhC*H*), 2.09 (s, 1H, O*H*), 0.94 (s, 9H, CH(CH_3)$_3$).

1-Phenyl-2-(trimethylsilyl)ethan-1-ol, S27

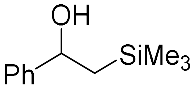

Mg (0.37 g, 15 mmol) was suspended in anhydrous Et_2O (2 mL), before two drops of a solution of chloromethyltrimethylsilane (1.5 g, 12 mmol) in anhydrous THF solution (1.7 mL) and a crystal of iodine were added. The suspension was heated with a heat gun until complete discoloration was shown. After the Mg was activated, the rest of the chloromethyltrimethylsilane solution was added dropwise and the reaction stirred for 1 h at rt. The freshly prepared Grignard was cooled to 0 °C before benzaldehyde (1.0 mL, 10 mmol) was added and reaction was stirred for 1 h at rt. The reaction was quenched with NH_4Cl and extracted with EtOAc (3×20 mL). The combined organic layers were washed with water, brine, dried over $MgSO_4$, filtered, and concentrated under reduced pressure. The crude product was purified by silica-gel column chromatography (Petrol/EtOAc, 95:5 to 80:20 R_f: 0.31) to give

title compound **S27** (0.73 g, 38%) as a colourless oil, with spectroscopic data in accordance with the literature.[6] ^{1}H NMR (300 MHz, $CDCl_3$) δ_H: 7.40–7.27 (m, 5H, ArC*H*), 4.90–4.80 (m, 1H, C*H*COH), 1.33–1.12 (m, 2H, C*H*$_2$Si), –0.08 (s, 9H, Si(C*H*$_3$)$_3$).

1-(3-Iodophenyl)ethan-1-ol, S28

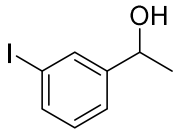

Following **General Procedure B**, 1-(3-iodophenyl)ethan-1-one (1.5 mL, 10 mmoL) and $NaBH_4$ (0.76 g, 20 mmol) were reacted in MeOH (50 mL). The reaction did not require further purification to give title compound **S28** (2.36 g, 96%) as a colourless oil, with spectroscopic data in accordance with the literature.[7] ^{1}H NMR (300 MHz, $CDCl_3$) δ_H: 7.83–7.76 (m, 1H, ArC*H*), 7.61–7.53 (m, 1H, ArC*H*), 7.44–7.31 (m, 1H, ArC*H*), 6.97 (td, J = 7.5, 1.8 Hz, 1H, ArC*H*), 5.07 (q, J = 6.4 Hz, 1H, C*H*CH_3), 1.46 (d, J = 6.4 Hz, 3H, CHC*H*$_3$).

1-(2-Iodophenyl)ethan-1-ol, S29

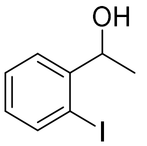

Following **General Procedure B**, 1-(2-iodophenyl)ethan-1-one (1.5 mL, 10 mmol) and $NaBH_4$ (0.76 g, 20 mmol) were reacted in MeOH (50 mL). The reaction did not require further purification to give title compound **S29** (2.35 g, 95%) as a colourless oil, with spectroscopic data in accordance with the literature.[8] ^{1}H NMR (300 MHz, $CDCl_3$) δ_H: 7.77–7.71 (m, 1H, ArC*H*), 7.60–7.52 (m, 1H, ArC*H*), 7.41–7.30 (m, 1H, ArC*H*), 7.08 (t, J = 7.8 Hz, 1H, ArC*H*), 4.85 (q, J = 6.4 Hz, 1H, C*H*CH_3), 1.48 (d, J = 6.5 Hz, 3H, CHC*H*$_3$).

1-(4-Methoxyphenyl)-2,2-dimethylpropan-1-ol, S30

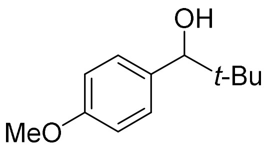

Following **General Procedure A**, 4-methoxybenzaldehyde (1.2 mL, 9.8 mmol) and *t*-BuMgCl (1.7 M in THF, 7 mL, 11.8 mmol) were reacted in THF (24 mL). The crude product was purified by silica-gel column chromatography (petrol/EtOAc, 95:5 to 85:15, R*f*: 0.39) to give title compound **S30** (1.54 g, 81%) as yellow oil, with spectroscopic data in accordance with the literature.[9] ^{1}H NMR (300 MHz, $CDCl_3$) δ_H: 7.25–7.21 (m, 2H, ArC(2,6)*H*), 6.88–6.83 (m, 2H,

ArC(3,5)*H*), 4.36 (s, 1H, ArC*H*OH), 3.81 (s, 3H, OCH_3), 0.91 (s, 9H, C(CH_3)$_3$).

2-(4-Methoxyphenyl)propan-2-ol, S31

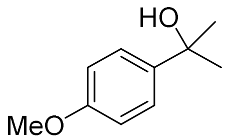

Following **General Procedure A**, 1-(4-methoxyphenyl)ethan-1-one (1.2 mL, 9.8 mmol) and MeMgCl (3 M in Et_2O, 4 mL, 11.8 mmol) were reacted in Et_2O (24 mL). The reaction did not require further purification to give title compound **S31** (1.45 g, 90%) as a colourless oil, with spectroscopic data in accordance with the literature.[10] ^{1}H NMR (300 MHz, $CDCl_3$) δ_H: 7.47–7.38 (m, 2H, ArC(2,6)*H*), 6.93–6.81 (m, 2H, ArC(3,5)*H*), 3.81 (s, 3H, OCH_3), 1.57 (s, 6H, C(CH_3)$_2$).

1-Phenylprop-2-en-1-ol S11

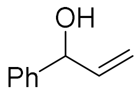

Following **General Procedure A**, benzaldehyde (1.04 mL, 9.8 mmol) and vinylmagnesium bromide (0.7 M in THF, 17.0 mL, 11.8 mmol) were reacted in THF (10 mL). The reaction did not require further purification to give title compound **S11** (1.31 g, 99%) as an orange oil, with spectroscopic data in accordance with the literature.[11] ^{1}H NMR (400 MHz, $CDCl_3$) δ_H: 7.41–7.33 (m, 4H, Ar*H*), 7.33–7.26 (m, 1H, Ar*H*), 6.11–6.00 (m, 1H, C*H*CH_2), 5.39–5.37 (m, 1H, PhC*H*OH), 5.23–5.18 (m, 2H, CHCH_2), 2.02 (s, 1H, O*H*).

1,3-Diketones

All other 1,3-diketones were used as received from commercial sources.

1,3-Bis(4-(trifluoromethyl)phenyl)propane-1,3-dione, S32

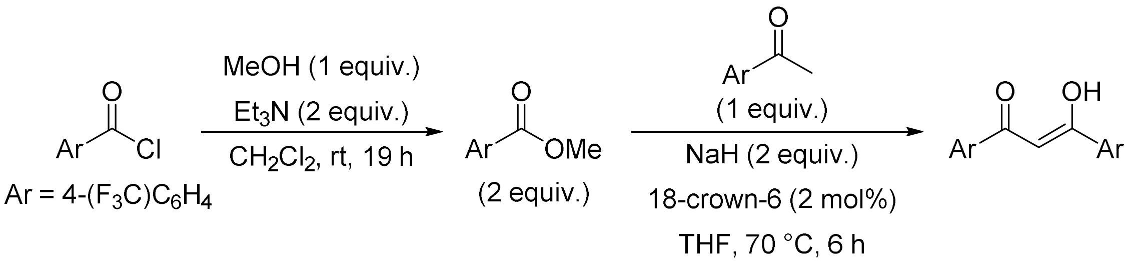

4-(Trifluoromethyl)benzoyl chloride (2.5 g, 12 mmol) was added slowly to a solution of MeOH (0.5 mL, 12.0 mmol) and Et_3N (3.5 mL, 24 mmol) were dissolved in anhydrous CH_2Cl_2 (36 mL) under a N_2 atmosphere. The reaction was stirred at rt for 19 h before being quenched with aq. 1 M HCl (10 mL). The layers were separated and the aqueous extracted with CH_2Cl_2 (3×50 mL). The combined organics were washed successively with aq. $NaHCO_3$, aq. $CuSO_4$, and brine before being dried over $MgSO_4$, filtered, and concentrated at reduced pressure to give methyl 4-(trifluoromethyl)benzoate (1.79 g, 73%) as a yellow oil, with spectroscopic data in accordance with the literature.[12] ^{1}H NMR (400 MHz, $CDCl_3$) δ_H: 8.16 (d, *J* = 8.0 Hz, 2H, ArC(3,5)*H*), 7.71 (d, *J* = 8.2 Hz, 2H, ArC(2,6)*H*), 3.96 (s, 3H, OC*H₃*).

Methyl 4-(trifluoromethyl)benzoate (1.22 g, 6.0 mmol) was dissolved in anhydrous THF (2 mL) under an Ar atmosphere before NaH (60% dispersion in mineral oil, 0.24 g, 6.0 mmol) was added. Absolute ethanol (2 drops) was added followed by a solution of 1-(4-(trifluoromethyl)phenyl)ethan-1-one (0.57 g, 3.0 mmol) in anhydrous THF (2 mL) and then a solution of 18-crown-6 (16 mg, 0.06 mmol) in anhydrous THF (2 mL). The reaction was stirred at rt for 30 min before being heated at reflux for 6 h. The solvent was removed under reduced pressure and the crude purified by silica-gel column chromatography (Petrol/PhMe, 50:50, R_f: 0.68) to give title compound **S32** (0.45 g, 45%) as an orange solid, with spectroscopic data in accordance with the literature.[13] mp 124–126 °C {Lit.[13] 130°C}; ^{1}H NMR (400 MHz, $CDCl_3$) δ_H: 8.10 (d, *J* = 8.1 Hz, 4H, ArC(3,5)*H*), 7.77 (d, *J* = 8.2 Hz, 4H, ArC(2,6)*H*), 6.88 (s, 1H, C*H*).

Silanes

Allyltrimethylsilane **26** was used as received from commercial sources.

Cinnamyltrimethylsilane, S33

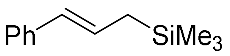

Mg (0.23 g, 9.20 mmol) was suspended in anhydrous THF (2 mL) before two drops of a solution of cinnamyl chloride (0.70 g, 4.60 mmol) in anhydrous THF (0.6 mL) and a crystal of iodine were added. The suspension was heated with a heat gun until complete discoloration was shown. After the Mg was activated, the reaction was cooled to –78 °C and the rest of the cinnamyl chloride solution was added dropwise followed by addition of TMSCl (2.4 mL, 18.4 mmol) and the reaction was stirred at –78 °C for 2 h followed by 30 min at rt. The reaction was quenched with cold water and extracted with EtOAc (3×20 mL). The combined organic layers were washed with brine, dried over $MgSO_4$, filtered, and concentrated under reduced pressure. The reaction did not require further purification to give title compound **S33** (0.80 g, 91%) as a colourless oil, with spectroscopic data in accordance with the literature.[14] ^{1}H NMR (300 MHz, $CDCl_3$) δ_H: 7.35–7.27 (m, 4H, ArC*H*), 7.20–7.12 (m, 1H, ArC(4)*H*), 6.26–6.22 (m, 2H, C*H*C*H*), 1.71–1.63 (m, 2H, CHCH_2), 0.05 (9H, s, Si(CH_3)$_3$).

Dehydrative Alkylation of 1,3-Diketone Derivatives

General Procedure C: Dehydrative alkylation of 1,3-diketone derivatives

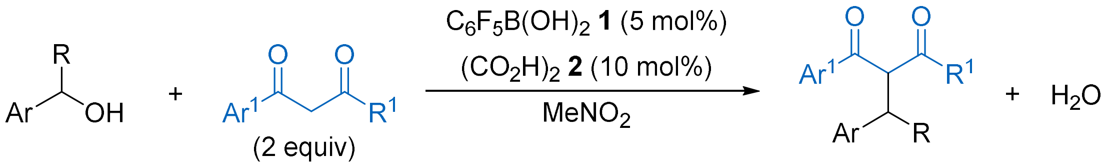

The required nucleophile (2.0 equiv.) was added to a solution of pentafluorophenylboronic acid **1** (5 mol%) and oxalic acid **2** (10 mol%) in $MeNO_2$ (0.05 M) and was stirred at rt for 5 mins. The required benzylic alcohol (1.0 equiv.) was added and the reaction stirred at the stated temperature until complete by TLC analysis. The reaction was cooled to rt, diluted with toluene and concentrated under reduced pressure. The crude product was purified by silica-gel column chromatography.

2-Benzhydryl-1,3-diphenylpropane-1,3-dione 3

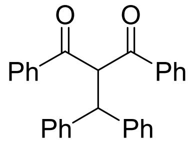

Following **General Procedure C,** benzhydrol (74 mg, 0.4 mmol), 1,3-diphenylpropane-1,3-dione (179 mg, 0.8 mmol), pentafluorophenylboronic acid **1** (4.2 mg, 20 µmol) and oxalic acid **2** (3.6 mg, 40 µmol) in MeNO (8.0 mL) were reacted at rt for 3 h. The crude was purified by trituration with cold Et_2O to give title compound **3** (119 mg, 76%) as a fine white powder, with spectroscopic data in accordance with the literature.[15] mp 226–227 °C {Lit.[15] 227–229 °C}; ^{1}H NMR (500 MHz, $CDCl_3$) δ_H: 7.91–7.82 (m, 4H, ArC*H*), 7.55–7.45 (m, 2H, ArC*H*), 7.40–7.32 (m, 4H, ArC*H*), 7.27 (d, *J* = 7.2 Hz, 4H, ArC*H*), 7.17 (t, *J* = 7.7 Hz, 4H, ArC*H*), 7.11–7.04 (m, 2H, ArC*H*), 6.38 (d, *J* = 11.6 Hz, 1H, OCC*H*), 5.35 (d, *J* = 11.6 Hz, 1H, PhCC*H*).

2-Benzhydryl-1,3-bis(4-methoxyphenyl)propane-1,3-dione 4

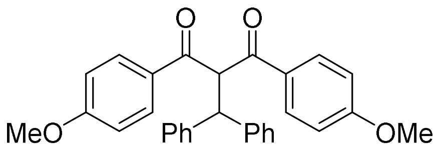

In a modified version of **General Procedure C,** benzhydrol (74 mg, 0.4 mmol), 1,3-bis(4-methoxyphenyl)propane-1,3-dione (570 mg, 2.0 mmol, 5 equiv.), pentafluorophenylboronic acid **1** (4.2 mg, 20 µmol) and oxalic acid **2** (3.6 mg, 40 µmol) in $MeNO_2$ (8.0 mL) were reacted at rt for 24 h. The crude product was purified by silica-gel column chromatography

(petrol/EtOAc, 95:5, R_f: 0.13) to give title compound **4** (107 mg, 59%) as a yellow solid, with spectroscopic data in accordance with the literature.[15] mp 182–183 °C {Lit.[15] 182–183.5 °C}; ^{1}H NMR (500 MHz, $CDCl_3$) δ_H: 7.93–7.85 (m, 4H, ArC*H*), 7.30–7.24 (m, 4H, ArC*H*), 7.13 (m, 4H, ArC*H*), 7.10–7.05 (m, 2H, ArC*H*), 6.87–6.80 (m, 4H, ArC*H*), 6.24 (d, *J* = 11.7 Hz, 1H, ArCOC*H*), 5.35 (d, *J* = 11.7 Hz, 1H, PhCC*H*), 3.83 (s, 6H, 2×OC*H$_3$*).

2-Benzhydryl-1,3-bis(4-(trifluoromethyl)phenyl)propane-1,3-dione 5

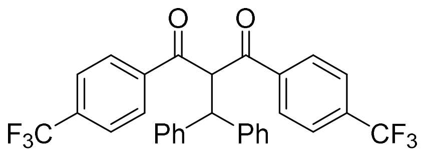

In a modified version of **General Procedure C,** benzhydrol (147 mg, 0.8 mmol, 2 equiv.), 1,3-bis(4-(trifluoromethyl)phenyl)propane-1,3-dione **S32** (145 mg, 0.4 mmol, 1 equiv.), pentafluorophenylboronic acid **1** (4.2 mg, 20 μmol) and oxalic acid **2** (3.6 mg, 40 μmol) in $MeNO_2$ (8.0 mL) were reacted at rt for 24 h. The crude product was purified by silica-gel column chromatography (petrol/EtOAc, 95:5, R_f: 0.27) to give title compound **5** (167 mg, 79%) as a white solid. mp 206–209 °C; ν_{max} (solid) 3061 (C-H), 1699 (C=O); ^{1}H NMR (500 MHz, $CDCl_3$) δ_H: 7.91 (d, *J* = 8.1 Hz, 4H, ArC*H*), 7.62 (d, *J* = 8.1 Hz, 4H, ArC*H*), 7.24–7.22 (m, 4H, ArC*H*), 7.17 (t, *J* = 7.6 Hz, 4H, ArC*H*), 7.11–7.04 (m, 2H, ArC*H*), 6.29 (d, *J* = 11.7 Hz, 1H, ArCOC*H*), 5.30 (d, *J* = 11.7 Hz, 1H, PhCC*H*); ^{13}C{^{1}H} NMR (126 MHz, $CDCl_3$) δ_C: 193.3 (ArC*C*O×2), 141.1 (Ph*C*CH×2), 139.5 (Ar*C*CO×2), 134.8 (q, $^2J_{C\text{-}F}$ = 32.9 Hz, Ar*C(4)*CF_3), 128.9 (q, $^3J_{C\text{-}F}$ 4.7, Ar*C*CF_3), 128.2 (Ar*C*CH×8), 127.2 (Ar*C*CH×2), 125.9 (q, $^3J_{C\text{-}F}$ = 3.5 Hz, Ar*C*CF_3), 123.5 (q, $^1J_{C\text{-}F}$ = 273 Hz, Ar*C*F_3), 63.5 (ArCO*C*H), 52.6 (ArC*C*H); ^{19}F{^{1}H} NMR (471 MHz, $CDCl_3$) δ_F: –63.26; HRMS (APCI$^+$) *m/z*: $[M+NH_4]^+$ calcd $C_{30}H_{24}F_6O_2N$ 544.1711, found 544.1710.

2-(Benzo[*d*]thiazol-2-yl)-1-(4-methoxyphenyl)-3,3-diphenylpropan-1-one, 6

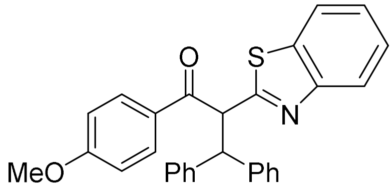

Following **General Procedure C,** benzhydrol (74 mg, 0.4 mmol), 2-(benzo[*d*]thiazol-2-yl)-1-(4-methoxyphenyl)ethan-1-one (227 mg, 0.8 mmol), pentafluorophenylboronic acid **1** (4.2 mg, 20 μmol) and oxalic acid **2** (3.6 mg, 40 μmol) in $MeNO_2$ (8.0 mL) were reacted at 90 °C for 48 h. The crude product was purified by silica-gel column chromatography (petrol/EtOAc, 90:10 to 70:30, R_f: 0.23) to give title compound **6** (151 mg, 98%) as a pale brown solid, mp

182–184 °C; ν_{max} (solid) 3022 (C-H), 1686 (C=O); ^{1}H NMR (500 MHz, $CDCl_3$) δ_H:

8.10 (d, *J* = 8.7 Hz, 2H, ArC(2,6)*H*), 7.92 (d, *J* = 8.1 Hz, 1H, ArC*H*), 7.74 (d, *J* = 8.0 Hz, 1H, ArC*H*), 7.44–7.33 (m, 5H, ArC*H*×5), 7.31–7.24 (m, 1H, ArC*H*), 7.20 (t, *J* = 7.6, 2H, ArC*H*), 7.15 (t, *J* = 7.6 Hz, 2H, ArC*H*), 7.12–7.07 (m, 1H, ArC*H*), 7.06–7.01 (m, 1H, ArC*H*), 6.89 (d, *J* = 8.7 Hz, 2H, ArC(3,5)*H*), 6.30 (d, *J* = 11.9 Hz, 1H, ArCCOC*H*), 5.23 (d, *J* = 11.9 Hz, 1H, PhCC*H*), 3.81 (s, 3H, OC*H₃*); ^{13}C{^{1}H} NMR (126 MHz, $CDCl_3$) δ_C: 194.6(ArC*C*O), 167.9 (SAr*C*N), 164.0 (Ar*C*), 152.2 (HetArN*C*), 142.3 (Ar*C*H), 141.3 (Ar*C*H), 135.7 (HetArN*C*), 131.4 (Ar*C*H), 129.6 (Ar*C*H), 128.8 (Ar*C*H), 128.6 (Ar*C*H), 127.8 (Ar*C*H×2), 126.8 (Ar*C*H), 126.4 (Ar*C*H), 125.9 (Ar*C*H), 125.1 (Ar*C*H), 123.0 (Ar*C*H), 121.7 (Ar*C*H), 114.0 (Ar*C*H), 56.8 (ArCCO*C*H), 55.6 (O*C*H₃), 54.9 (PhCH*C*H); HRMS (APCI$^+$) *m/z*: $[M+H]^+$ calcd for $C_{29}H_{24}NO_2S$ 450.1522, found 450.1521.

2-(Benzo[*d*]oxazol-2-yl)-1,3,3-triphenylpropan-1-one, 7

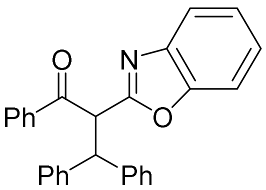

Following **General Procedure C**, benzhydrol (74 mg, 0.4 mmol), 2-(benzo[*d*]oxazol-2-yl)-1-phenylethan-1-one (191 mg, 0.8 mmol), pentafluorophenylboronic acid **1** (4.2 mg, 20 μmol) and oxalic acid **2** (3.6 mg, 40 μmol) in $MeNO_2$ (8.0 mL) were reacted at 90 °C for 48 h. The crude product was purified by silica-gel column chromatography (petrol/EtOAc, 90:10 to 70:30, R_f: 0.23) to give title compound **7** (79 mg, 49%) as a white solid. mp 215–218 °C; ν_{max} (film) 3059 (C-H Ar), 3030 (C-H alkane), 1700 (C=O ketone), 1540 (C=C Ar), 1069 (C-N), 937 (C=C Ar); ^{1}H NMR (400 MHz, $CDCl_3$) δ_H: 8.13–7.98 (m, 2H, ArC*H*), 7.62–7.58 (m, 1H, ArC*H*), 7.56–7.52 (m, 1H, ArC*H*), 7.47–7.41 (m, 3H, ArC*H*×3), 7.38–7.34 (m, 2H, ArC*H*), 7.34–7.30 (m, 2H, ArC*H*), 7.27–7.20 (m, 4H, ArC*H*×4), 7.14–7.09 (m, 3H, ArC*H*×3), 7.07–6.98 (m, 1H, ArC*H*), 6.07 (d, *J* = 12.0 Hz, 1H, ArCOC*H*), 5.40 (d, *J* = 12.0 Hz, 1H, PhCC*H*); ^{13}C{^{1}H} NMR (126 MHz, $CDCl_3$) δ_C: 193.2 (ArC*C*O), 161.7 (Ar*C*N), 150.9 (Ar*C*), 141.6 (Ar*C*), 141.2 (Ar*C*), 140.9 (Ar*C*), 136.3 (Ar*C*), 133.7 (Ar*C*H), 128.8 (Ar*C*H), 128.8 (Ar*C*H), 128.7 (Ar*C*H), 128.6 (Ar*C*H), 128.0 (Ar*C*H), 127.8 (Ar*C*H), 126.8 (Ar*C*H×2), 125.0 (Ar*C*H), 124.3 (Ar*C*H), 120.0 (Ar*C*H), 110.6 (Ar*C*H), 52.6 (ArCO*C*H), 51.8 (PhC*C*H); HRMS (NSI$^+$) *m/z:* $[M+H]^+$ calcd for $C_{28}H_{22}O_2N$ 404.1645, found 404.1645.

2-Benzhydryl-2-methylcyclopentane-1,3-dione, 8

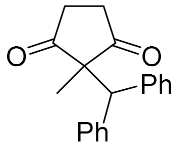

Following **General Procedure C**, benzhydrol (74 mg, 0.4 mmol, 50 μL), 2-methylcyclopentane-1,3-dione (90 mg, 0.8 mmol), pentafluorophenylboronic acid **1** (4.2 mg, 20 μmol) and oxalic acid **2** (3.6 mg, 40 μmol) in $MeNO_2$ (8.0 mL) were reacted at 90 °C for 16 h. The crude product was purified by silica-gel column chromatography (petrol/EtOAc, 95:5 to 80:20 R_f: 0.23) to give title compound **8** (103 mg, 93%) as a light yellow solid. mp 132–133 °C; ν_{max} 3030 (C–H), 2917 (C–H), 1716 (C=C), 1598 (C=C); ^1H NMR (300 MHz, $CDCl_3$) δ_H: 7.47–7.41 (m, 4H, ArC*H*), 7.31–7.27 (m, 2H, ArC*H*), 7.26–7.18 (m, 4H, ArC*H*), 4.36 (s, 1H, PhCC*H*), 2.67–2.48 (m, 2H, CH_2CH_2), 2.13–2.05 (m, 2H, CH_2CH_2), 1.11 (s, 3H, CH_3); $^{13}C\{^1H\}$ NMR (126 MHz, $CDCl_3$) δ_C: 217.8 (*C*O), 139.7 (Ar*C*(1)), 129.7 (Ar*C*H), 128.7 (Ar*C*H), 127.3 (Ar*C*H), 60.5 (CCH_3), 58.5 (Ar*C*HAr), 36.1 (CH_2CH_2), 20.2 (CCH_3); HRMS (ESI^-) *m/z*: $[M–H]^-$ calcd for $C_{19}H_{17}O_2^-$ 277.1234, found 277.1234.

1,3-Diphenyl-2-(1-phenylethyl)propane-1,3-dione, 9

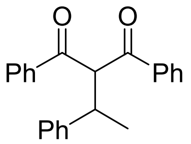

Following **General Procedure C**, 1-phenylethan-1-ol **S18** (52 mg, 0.4 mmol), 1,3-diphenylpropane-1,3-dione **2** (180 mg, 0.8 mmol), pentafluorophenylboronic acid **1** (4.2 mg, 20 μmol) and oxalic acid **2** (3.6 mg, 40 μmol) in $MeNO_2$ (8.0 mL) were reacted at 90 °C for 24 h. The crude product was purified by silica-gel column chromatography (petrol/EtOAc, 93:7 R_f:0.30) to give title compound **9** (123 mg, 88%) as a white solid, with spectroscopic data in accordance with the literature.[16] mp 120–122 °C {Lit.[16] 128–129 °C}; ^1H NMR (400 MHz, $CDCl_3$) δ_H: 8.13–8.02 (m, 2H, ArC*H*CO), 7.79–7.74 (m, 2H, ArC*H*CO), 7.62–7.56 (m, 1H, ArC*H*CO), 7.50–7.42 (m, 1H, ArC*H*CO), 7.34–7.27 (m, 4H, ArC*H* and ArC*H*CO), 7.23–7.17 (m, 2H, ArC*H*), 7.15–7.07 (m, 1H, ArC*H*), 5.63 (d, J = 10.1 Hz, 1H, PhCOC*H*), 4.17–4.04 (m, 1H, PhCC*H*), 1.37 (d, J = 7.0 Hz, 3H, $CHCH_3$).

Gram scale: Following **General Procedure C**, 1-phenylethan-1-ol **S18** (0.49 mL, 4.00 mmol), 1,3-diphenylpropane-1,3-dione (1.79 g, 8.00 mmol), pentafluorophenylboronic acid **1** (42 mg, 0.20 mmol) and oxalic acid **2** (36 mg, 0.400 mmol) in $MeNO_2$ (80 mL) were reacted at 90 °C for 19 h. The crude product was purified by silica-gel column chromatography (petrol/EtOAc,

93:7 R_f:0.30) to give title compound **9** (1.225 g, 93%) as a white powder, with spectroscopic data as above. Excess 1,3-diphenylpropane-1,3-dione was also recovered from the column (R_f:0.53, 0.616 g, 69%) as a pale orange powder.

2-(1-(4-Methoxyphenyl)ethyl)-1,3-diphenylpropane-1,3-dione, 10

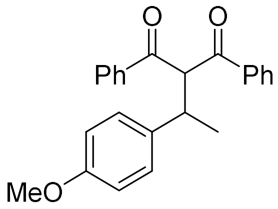

Following **General Procedure C**, 1-(4-methoxyphenyl)ethan-1-ol **S19** (61 mg, 0.4 mmol), 1,3-diphenylpropane-1,3-dione (180 mg, 0.8 mmol), pentafluorophenylboronic acid **1** (4.2 mg, 20 µmol) and oxalic acid **2** (3.6 mg, 40 µmol) in $MeNO_2$ (8.0 mL) were reacted at 90 °C for 24 h. The crude product was purified by silica-gel column chromatography (petrol/EtOAc, 93:7 R_f:0.26) to give title compound **10** (103 mg, 70%) as an off-white crystalline solid, with spectroscopic data in accordance with the literature.[16] mp 106–108 °C{Lit.[16] 109–113 °C}; ^{1}H NMR (400 MHz, $CDCl_3$) δ_H: 8.14–7.95 (m, 2H, ArC*H*CO), 7.79–7.69 (m, 2H, ArC*H*CO), 7.61–7.50 (m, 1H, ArC*H*CO), 7.49–7.37 (m, 3H, ArC*H*CO), 7.32–7.23 (m, 2H, ArC*H*CO), 7.21–7.12 (m, 2H, ArC(2,6)*H*), 6.80–6.65 (m, 2H, ArC(3,5)*H*), 5.55 (d, J = 10.1 Hz, 1H, ArCOC*H*), 4.04 (dq, J = 10.1, 7.0 Hz, 1H, ArCC*H*), 3.70 (s, 3H, OCH_3), 1.32 (d, J = 7.0 Hz, 3H, CHCH_3).

2-(1-(2-Methoxyphenyl)ethyl)-1,3-diphenylpropane-1,3-dione, 11

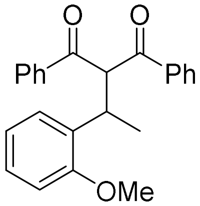

Following **General Procedure C**, 1-(2-methoxyphenyl)ethan-1-ol **S20** (61 mg, 0.4 mmol), 1,3-diphenylpropane-1,3-dione (180 mg, 0.8 mmol), pentafluorophenylboronic acid **1** (4.2 mg, 20 µmol) and oxalic acid **2** (3.6 mg, 40 µmol) in $MeNO_2$ (8.0 mL) were reacted at 40 °C for 48 h. The crude product was purified by silica-gel column chromatography (petrol/EtOAc, 100:0 to 85:15 R_f: 0.35) to give title compound **11** (136 mg, 95%) as colorless oil. ν_{max} (film) 3062 (C-H Ar), 2962 (C-H alkane), 1693 (C=O Ketone), 1662 (C=C Ar), 1250 (C-O ether), 810 (C-H Ar); ^{1}H NMR (400 MHz, $CDCl_3$) δ_H: 8.07–7.95 (m, 2H, ArC*H*), 7.92–7.81 (m, 2H, ArC*H*), 7.61–7.52 (m, 3H, ArC*H*), 7.52–7.41 (m, 3H, PhC*H*), 7.38–7.31 (m, 2H, ArC*H*), 7.25–

7.20 (m, 1H, ArC*H*), 7.15–7.07 (m, 1H, ArC*H*), 6.86–6.78 (m, 1H, ArC*H*), 6.78–6.72 (m, 1H, ArC*H*), 5.99 (d, *J* = 8.8 Hz, 1H, ArCOC*H*), 4.31–4.14 (m, 1H, ArCC*H*), 3.86 (s, 3H, OC*H₃*), 1.37 (d, *J* = 7.1 Hz, 3H, CHC*H₃*); $^{13}C\{^1H\}$ NMR (126 MHz, $CDCl_3$) δ_C: 196.0 (*C*O), 195.5 (*C*O), 157.2 (*C*(2)OCH_3), 137.6 (Ar*C*(1)), 137.0 (Ar*C*(1)), 133.3 (Ar*C*(4)H), 133.0 (Ar*C(4)*H), 131.7 (ArC(1)), 128.8 (Ar*C(2,6)*H×2), 128.6 (Ar*C(3,5)*H×2), 127.8 (Ar*C*(4,6)*H*), 120.8 (Ar*C*(3)*H*), 110.8 (Ar*C*(5*)H)*, 61.1 (OC*C*H), 55.2 (O*C*H_3), 37.03 (CH*C*H_3), 17.9 (*C*HCH_3); HRMS ($APCI^+$) *m/z*: $[M+H]^+$ calcd for $C_{24}H_{23}O_3$ 359.1642, found 359.1643.

2-(1-(4-Fluorophenyl)ethyl)-1,3-diphenylpropane-1,3-dione, 12

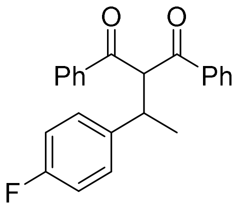

Following **General Procedure C**, 1-(4-fluorophenyl)ethan-1-ol **S21** (56 mg, 0.4 mmol), 1,3-diphenylpropane-1,3-dione (180 mg, 0.8 mmol), pentafluorophenylboronic acid **1** (4.2 mg, 20 µmol) and oxalic acid **2** (3.6 mg, 40 µmol) in $MeNO_2$ (8.0 mL) were reacted at 90 °C for 24 h. The crude product was purified by silica-gel column chromatography (petrol/EtOAc, 100:0 to 85:15 R_f:0.21) to give title compound **12** (131 mg, 90%) as a white solid, with spectroscopic data in accordance with the literature.[17] mp 109–110 °C {Lit.[17] 110–112 °C}; ^1H NMR (400 MHz, $CDCl_3$) δ_H: 8.06–8.00 (m, 2H, ArC*H*CO), 7.77–7.71 (m, 2H, ArC*H*CO), 7.61–7.53 (m, 1H, ArC*H*CO), 7.50–7.40 (m, 3H, ArC*H*CO), 7.35–7.27 (m, 2H, ArC*H*CO), 7.25–7.18 (m, 2H, ArC(2,6)*H*), 6.90–6.82 (m, 2H, ArC(3,5)*H*), 5.54 (d, *J* = 10.2 Hz, 1H, ArCCOC*H*), 4.16–4.07 (m, 1H, ArCC*H*), 1.35 (d, *J* = 7.0 Hz, 3H, CHC*H*₃).

2-(1-(4-Bromophenyl)ethyl)-1,3-diphenylpropane-1,3-dione, 13

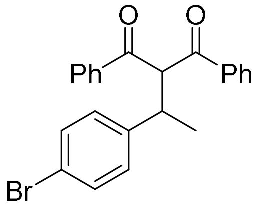

Following **General Procedure C**, 1-(4-bromophenyl)ethan-1-ol **S22** (81 mg, 0.4 mmol), 1,3-diphenylpropane-1,3-dione (180 mg, 0.8 mmol), pentafluorophenylboronic acid **1** (4.2 mg, 20 µmol) and oxalic acid **2** (3.6 mg, 40 µmol) in $MeNO_2$ (8.0 mL) were reacted at 90 °C for 24 h. The crude product was purified by silica-gel column chromatography (petrol/EtOAc, 100:0 to 85:15 R_f:0.21) to give title compound **13** (80 mg, 71%) as a white solid, with spectroscopic

data in accordance with the literature.[18] mp 83–85 °C {Lit.[18] 82–84 °C}; ^{1}H NMR (400 MHz, $CDCl_3$) δ_H: 8.11–8.01 (m, 2H, ArC*H*), 7.84–7.74 (m, 2H, ArC(3,5)*H*), 7.67–7.56 (m, 1H, ArC*H*), 7.56–7.43 (m, 2H, ArC*H*), 7.40–7.27 (m, 4H, ArC*H*), 7.21–7.11 (m, 2H, ArC(2,6)*H*), 5.55 (d, *J* = 10.2 Hz, 1H, ArCOC*H*), 4.15–4.00 (m, 1H, ArCC*H*), 1.38 (d, *J* = 7.0 Hz, 3H, CHC*H*$_3$).

2-(1-(3-Bromophenyl)ethyl)-1,3-diphenylpropane-1,3-dione, 14

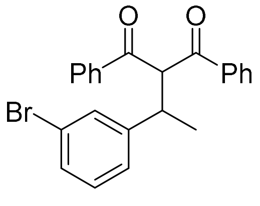

Following **General Procedure C**, 1-(3-bromophenyl)ethan-1-ol **S23** (81 mg, 0.4 mmol), 1,3-diphenylpropane-1,3-dione (180 mg, 0.8 mmol), pentafluorophenylboronic acid **1** (4.2 mg, 20 µmol) and oxalic acid **2** (3.6 mg, 40 µmol) in $MeNO_2$ (8.0 mL) were reacted at 90 °C for 24 h. The crude product was purified by silica-gel column chromatography (petrol/EtOAc, 100:0 to 85:15 R_f:0.21) to give title compound **14** (43 mg, 38%) as a white powder; mp 111–113 °C; ν_{max} (film) 3061 (C-H Ar), 2962 (C-H), 1705 (C=O), 1595 (C=C Ar), 783 (C-H Ar), 758 (C-Br); ^{1}H NMR (300 MHz, $CDCl_3$) δ_H: 8.12–8.01 (m, 2H, ArC*H*), 7.86–7.74 (m, 2H, ArC*H*), 7.63–7.52 (m, 2H, ArC*H*), 7.56–7.40 (m, 4H, ArC*H*), 7.24–7.16 (m, 2H, ArC*H*), 7.08–7.01 (m, 2H, ArC*H*), 5.59 (d, *J* = 10.1 Hz, 1H, ArCOC*H*), 4.21–4.08 (m, 1H, ArCC*H*), 1.34 (d, *J* = 6.9 Hz, 3H, CHC*H*$_3$); ^{13}C{^{1}H} NMR (126 MHz, $CDCl_3$) δ_C: 194.7 (*C*O), 194.4 (*C*O), 146.3 (Ar*C*(1)), 136.7 (Ar*C*(1)), 136.7 (Ar*C*(1)), 133.8 (Ar*C*(4)H), 133.4 (Ar*C*(4)H), 130.8 (Ar*C(2)H*), 130.1 (Ar*C*(5)H), 129.9 (Ar*C*(4)H), 129.0 (Ar*C*(2,6)H), 128.9 (Ar*C*(2,6)H), 128.7 (Ar*C*(3,5)*H*), 128.6 (Ar*C*(3,5)H), 126.8 (Ar*C*(6)H), 122.5 (Ar*C*(3)Br), 64.4 (ArCO*C*H), 40.9 (CH*C*H$_3$), 20.2 (*C*HCH$_3$); HRMS (APCI$^+$) *m/z*: $[M+H]^+$ calcd for $C_{23}H_{20}BrO_2$ 407.0641, found 407.0639.

2-(1-(2-Bromophenyl)ethyl)-1,3-diphenylpropane-1,3-dione, 15

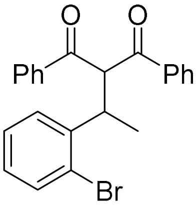

Following **General Procedure C**, 1-(2-bromophenyl)ethan-1-ol **S24** (89 mg, 0.4 mmol), 1,3-diphenylpropane-1,3-dione (180 mg, 0.8 mmol), pentafluorophenylboronic acid **1** (4.2 mg, 20 μmol) and oxalic acid **2** (3.6 mg, 40 μmol) in $MeNO_2$ (8.0 mL) were reacted at 90 °C for 48 h. The crude product was purified by silica-gel column chromatography (petrol/EtOAc, 100:0 to 85:15 R_f:0.35) to give title compound **15** (83 mg, 51%) as a white solid, with spectroscopic data in accordance with the literature.[19] mp 121–123 °C {Lit.[19] 122–124 °C}; ^{1}H NMR (300 MHz, $CDCl_3$) δ_H: 8.01–7.93 (m, 2H, ArC*H*), 7.93–7.86 (m, 2H, ArC*H*), 7.61–7.48 (m, 3H, ArCH), 7.48–7.40 (m, 2H, ArC*H*), 7.40–7.33 (m, 2H, ArC*H*), 7.23–7.18 (m, 1H, ArC*H*), 7. 16–7.10 (m, 1H, ArC*H*), 7.04–6.97 (m, 1H, ArC*H*), 5.59 (d, *J* = 10.1 Hz, 1H, ArCOC*H*), 4.57–4.43 (m, 1H, ArCC*H*), 1.35 (d, *J* = 7.1 Hz, 3H, CHC*H*$_3$).

2-(1-(4-Methoxyphenyl)prop-2-yn-1-yl)-1,3-diphenylpropane-1,3-dione, 16

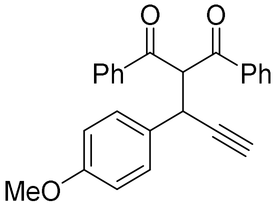

Following **General Procedure C**, 1-(4-methoxyphenyl)prop-2-yn-1-ol **S25** (32 mg, 0.2 mmol), 1,3-diphenylpropane-1,3-dione (90 mg, 0.4 mmol), pentafluorophenylboronic acid **1** (2.2 mg, 10 μmol) and oxalic acid **2** (1.8 mg, 20 μmol) in $MeNO_2$ (4.0 mL) were reacted at rt for 16 h. The crude product was purified by silica-gel column chromatography (petrol/EtOAc, 100:0 to 80:20 R_f:0.22) to give title compound **16** (72 mg, 98%) as a white solid. mp 106–112 °C; ν_{max} 3030 (C–H), 2916 (C–H), 1716 (C=O), 1597 (C=C); ^{1}H NMR (300 MHz, $CDCl_3$) δ_H: 8.06–7.98 (m, 2H, ArC*H*CO), 7.74–7.69 (m, 2H, ArC*H*CO), 7.61–7.53 (m, 3H, ArC*H*CO), 7.49–7.41 (m, 3H, ArC*H*CO), 7.39–7.27 (m, 2H, ArC(2,6)*H*), 6.79–6.71 (m, 2H, ArC(3,5)*H*), 5.79 (d, *J* = 10.2 Hz, 1H, ArCCOC*H*), 4.92 (dd, *J* = 10.2, 2.5 Hz, 1H, ArCC*H*CCH), 3.71 (s, 3H, OC*H*$_3$), 2.18 (d, *J* = 2.5 Hz, 1H, CHCC*H*); ^{13}C{^{1}H} NMR (126 MHz, $CDCl_3$) δ_C: 193.3 (*C*O), 192.9 (*C*O), 159 (Ar*C*(4)OMe), 133.7 (Ar*C*(4)H), 133.5 (Ar*C*(4)H), 130.4 (Ar*C*(1)), 129.7 (Ar*C*(2,6)H), 128.8 (Ar*C*H), 128.8 (Ar*C*H), 128.8 (Ar*C*H), 128.7 (Ar*C*H), 114.2 (Ar*C*(3,5)H), 84.3 (*C*CH), 72.7 (C*C*H), 63.8 (CO*C*HCO), 55.4 (O*C*H$_3$), 37.5 (*C*HC); HRMS

(ESI$^-$) *m/z*: [M–H]$^-$ calcd for $C_{24}H_{22}O_4$ 374.1518, found 374.1500.

3-(Benzhydryloxy)cyclohex-2-en-1-one, S17

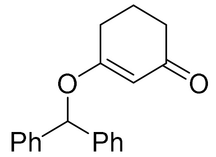

Following **General Procedure C**, cyclohexane-1,3-dione (43 mg, 0.4 mmol), benzhydrol (150 mg, 0.8 mmol), pentafluorophenylboronic acid **1** (4.2 mg, 20 μmol) and oxalic acid **2** (3.6 mg, 40 μmol) in $MeNO_2$ (8.0 mL) were reacted at 40 °C for 16 h. The crude product was purified by silica-gel column chromatography (petrol/EtOAc, 90:10 to 40:60 R_f: 0.12) to give title compound **S17** (81 mg, 73%) as a yellow oil. ν_{max} 3029 (C–H), 2934 (C–H), 1646 (C=C), 1610 (C=C), 1582 (C=C); ^{1}H NMR (300 MHz, $CDCl_3$) δ_H: 7.39–7.28 (m, 10H, ArC*H*), 6.11 (s, 1H, OC*H*), 5.37 (s, 1H, C*H*), 2.55 (t, *J* = 6.2 Hz, 2H, CO*CH₂*CH₂*CH₂*), 2.31 (dd, *J* = 7.3, 5.9 Hz, 2H, *C*H₂CH₂*CH₂*COCH), 2.09–1.91 (m, 2H, CH₂C*H₂*CH₂); ^{13}C{^{1}H} NMR (126 MHz, $CDCl_3$) δ_C: 199.9 (*C*O), 176.6 (COCH*C*O), 144.0 (Ar*C*(1)), 139.8 (Ar*C*(1)), 128.9 (Ar*C*H), 128.7 (Ar*C*H), 128.6 (Ar*C*H), 128.4 (Ar*C*H), 127.7 (Ar*CH*), 127.6 (Ar*CH*), 127.4 (Ar*C*H), 126.8 (Ar*CH*), 105.5 (CO*C*HCO), 76.4 (Ar*C*HAr), 36.8 (CO*C*H₂), 29.4 (CO*C*H₂), 20.7 (CH₂*C*H₂CH₂); HRMS (ESI$^-$) *m/z*: [M–H]$^-$ calcd for $C_{19}H_{18}O_2$ 278.1307, found 278.1302.

Dehydrative Alkylation of 1,3-Ketoesters

General Procedure D: Dehydrative alkylation of 1,3-ketoesters

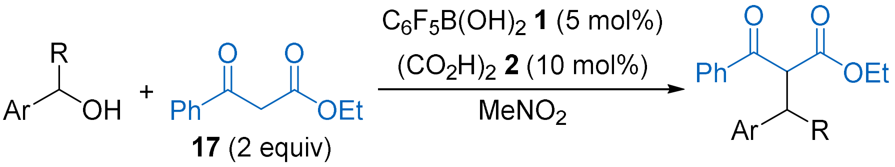

The ethyl benzoylacetate **17** (2.0 equiv.) was added to a solution of pentafluorophenylboronic acid **1** (5 mol%) and oxalic acid **2** (10 mol%) in $MeNO_2$ (0.05 M) and was stirred at rt for 5 mins. The required benzylic alcohol (1.0 equiv.) was added and the reaction stirred at the stated temperature until complete by TLC analysis. The reaction was cooled to rt, diluted with toluene and concentrated under reduced pressure. The crude product was purified by silica-gel column chromatography.

Ethyl 2-benzhydryl-3-oxo-3-phenylpropanoate, 18

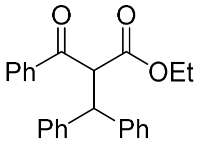

In a modified version of **General Procedure D,** benzhydrol (147 mg, 0.8 mmol, 2 equiv.), ethyl benzoylacetate **17** (80 mg, 0.4 mmol, 1 equiv.), pentafluorophenylboronic acid **1** (4.2 mg, 20 μmol) and oxalic acid **2** (3.6 mg, 40 μmol) in $MeNO_2$ (8.0 mL) were reacted at 90 °C for 24 h. The crude product was purified by silica-gel column chromatography (petrol/EtOAc, 99:1 to 90:10, R_f:0.30) to give title compound **18** (144 mg, 97%) as a white solid, with spectroscopic data in accordance with the literature.[15] mp 133–135 °C {Lit.[15] 138–140 °C}; ^{1}H NMR (400 MHz, $CDCl_3$) δ_H: 8.05–8.01 (m, 2H, ArC*H*CO), 7.59–7.53 (m, 1H, ArC*H*CO), 7.47–7.37 (m, 4H, ArC*H* and ArC*H*CO), 7.32–7.23 (m, 4H, ArC*H*), 7.22–7.13 (m, 3H, ArC*H*), 7.09–7.04 (m, 1H, ArC*H*), 5.43 (d, *J* = 11.8 Hz, 1H, ArCCOC*H*), 5.10 (d, *J* = 11.8 Hz, 1H, ArCC*H*), 3.95–3.90 (m, 2H, CH_2CH_3), 0.94 (t, *J* = 7.1 Hz, 3H, CH_2CH_3).

Ethyl 2-benzoyl-3-phenylbutanoate, 19

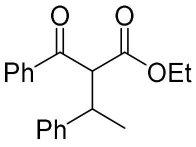

In a modified version of **General Procedure D,** 1-phenylethan-1-ol **S18** (100 mg, 0.8 mmol, 2 equiv.), ethyl benzoylacetate **17** (77 mg, 0.4 mmol, 1 equiv.), pentafluorophenylboronic acid

1 (4.2 mg, 20 μmol) and oxalic acid **2** (3.6 mg, 40 μmol) in $MeNO_2$ (8.0 mL) were reacted at 90 °C for 16 h. The crude product was purified by silica-gel column chromatography (petrol/EtOAc, 98:2 to 90:10 R_f: 0.19) to give **19** as a mixture of diastereoisomers (57:43 dr, 211 mg, 98%) as a colourless oil, with spectroscopic data in accordance with the literature.[16]

Data for major diastereoisomer: ^{1}H NMR (300 MHz, $CDCl_3$) (*selected)* δ_H: 8.14–8.07 (m, 2H, ArC*H*), 7.64–7.57 (m, 1H, ArC*H*), 7.55–7.44 (m, 2H, ArC*H*), 7.41–7.28 (m, 3H, ArC*H*), 7.24–7.06 (m, 2H, ArC*H*), 4.64 (d, *J* = 10.6 Hz, 1H, ArCCOC*H*), 4.26–4.14 (m, 1H, C*H*CH_3), 3.89–3.75 (m, 2H, C$H_2$$CH_3$), 1.32–1.25 (m, 3H, CHC$H_3$), 0.88 (t, *J* = 7.1 Hz, 3H, OCH_2CH_3).

Data for minor diastereoisomer: ^{1}H NMR (300 MHz, $CDCl_3$) (*selected)* δ_H: 8.01–7.92 (m, 2H, ArC*H*), 7.89–7.80 (m, 2H, ArC*H*), 7.64–7.57 (m, 1H, ArC*H*), 7.41–7.28 (m, 3H, ArC*H*), 7.24–7.06 (m, 2H, ArC*H*), 4.69 (d, *J* = 10.6 Hz, 1H, ArCCOC*H*), 4.26–4.14 (m, 1H, C*H*CH_3), 3.89–3.75 (m, 2H, C$H_2$$CH_3$), 1.24–1.19 (m, 3H, OCH_2CH_3).

Ethyl 2-benzoyl-3-(*p*-tolyl)butanoate, 20

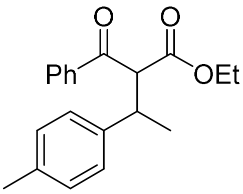

In a modified version of **General Procedure D,** 1-(*p*-tolyl)ethan-1-ol (871 mg, 6.4 mmol, 2 equiv.), ethyl benzoylacetate **17** (615 mg, 3.2 mmol, 1 equiv.), pentafluorophenylboronic acid **1** (34 mg, 0.16 mmol) and oxalic acid **2** (29 mg, 0.32 mmol) in $MeNO_2$ (64 mL) were reacted at 90 °C for 24 h. The crude product was purified by silica-gel column chromatography (petrol/EtOAc, 98:2 to 90:10 R_f: 0.22) to give **20** as a mixture of diastereoisomers (54:46 dr, 970 mg, 97%) as a colourless oil. ν_{max} 2973 (C–H), 1733 (C=C), 1685 (C=C), 1515 (C=C);

Data for major diastereoisomer: ^{1}H NMR (300 MHz, $CDCl_3$) (*selected)* δ_H: 8.15–8.10 (m, 2H, ArC*H*), 7.66–7.60 (m, 1H, ArC*H*), 7.55–7.49 (m, 2H, ArC*H*), 7.16–7.10 (m, 2H, ArC*H*), 7.03–6.98 (m, 2H, ArC*H*), 4.64 (d, *J* = 10.7 Hz, 1H, ArCCOC*H*), 4.32–4.18 (m, 1H, C*H*CH_3), 3.91–3.77 (m, 2H, OC$H_2$$CH_3$), 2.34 (s, 3H, ArCC$H_3$), 1.29–1.24 (m, 3H, CHC$H_3$), 0.93 (t, *J* = 7.1 Hz, 3H, OCH_2CH_3); ^{13}C{^{1}H} NMR (126 MHz, $CDCl_3$) δ_C: 193.8 (ArC*C*O), 168.2 (*C*OOEt), 140.6 (Ar*C*), 136.9 (Ar*C*(4)CH_3), 136.1 (Ph*C(1)*), 133.8 (Ph*C*H), 129.2 (Ar*C*H), 128.9 (Ph*C*H), 128.6 (Ph*C*H), 127.4 (Ar*C*H), 61.3 (ArCCO*C*H), 46.2 (*C*$H_2$$CH_3$), 39.4 (*C*H$CH_3$), 21.1 (ArC*C*$H_3$), 20.5 (CH*C*$H_3$), 13.8 ($CH_2$*C*$H_3$); HRMS ($ESI^+$) *m/z*: $[M+Na]^+$ calcd for $C_{20}H_{22}O_3Na$ 333.1461, found 333.1468.

Data for minor diastereoisomer: ^{1}H NMR (300 MHz, $CDCl_3$) (*selected*) δ_H: 7.90–7.86 (m, 2H, ArC*H*), 7.66–7.60 (m, 1H, ArC*H*), 7.55–7.49 (m, 2H, ArC*H*), 7.25–7.20 (m, 3H, ArC*H*), 7.16–7.10 (m, 2H, ArC*H*), 4.69 (d, *J* = 10.5 Hz, 1H, ArCCOC*H*), 4.32–4.18 (m, 1H, C*H*CH_3), 3.91–3.77 (m, 2H, C$H_2$$CH_3$), 2.24 (s, 3H, ArC$H_3$), 1.24–1.19 (m, 3H, OCH_2CH_3); ^{13}C{^{1}H} NMR (126 MHz, $CDCl_3$) (*selected*) δ_C: 194.1 (ArC*C*O), 168.9 (*C*OOEt), 141.0 (Ar*C*), 137.1 (Ar*C*(4)CH_3), 136.4 (Ar*C*(1)), 133.9 (Ar*C*H), 127.7 (Ar*C*H), 61.6 (ArCCO*C*H), 46.2 (*C*$H_2$$CH_3$), 40.0 (*C*H$CH_3$), 21.2 (ArC$CH_3$), 20.9 (CH*C*$H_3$), 14.2 ($CH_2$*C*$H_3$).

Ethyl 2-benzoyl-3-(4-methoxyphenyl)butanoate, 21

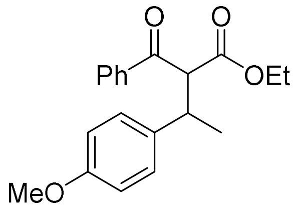

Following **General Procedure D,** 1-(4-methoxyphenyl)ethan-1-ol **S19** (61 mg, 0.4 mmol), ethyl benzoylacetate **17** (154 mg, 0.8 mmol), pentafluorophenylboronic acid **1** (4.2 mg, 20 μmol) and oxalic acid **2** (3.6 mg, 40 μmol) in $MeNO_2$ (8.0 mL) were reacted at 40 °C for 24 h. The crude product was purified by silica-gel column chromatography (petrol/EtOAc, 95:5 to 85:15 R_f: 0.25) to give **21** as a mixture of diastereoisomers (53:47 dr, 110 mg, 80%) as a colourless oil, with spectroscopic data in accordance with the literature.[16]

Data for major diastereoisomer: ^{1}H NMR (400 MHz, $CDCl_3$) δ_H: 8.11–8.08 (m, 2H, PhC(2,6)*H*), 7.61–7.57 (m, 1H, PhC(4)*H*), 7.51–7.46 (m, 2H, PhC(3,5)*H*), 7.24 (d, *J* = 8.7 Hz, 2H, ArC(2,6)*H*), 6.85 (d, *J* = 8.7 Hz, 2H, ArC(3,5)*H*), 4.59 (d, *J* = 10.6 Hz, 1H, C(O)C*H*), 3.90–3.74 (m, 3H, OCH_2 and C*H*CH_3), 3.78 (s, 3H, OCH_3), 1.23 (d, *J* = 6.7 Hz, 3H, CHCH_3), 0.91 (t, *J* = 7.2 Hz, 3H, OCH_2CH_3).

Data for minor diastereoisomer: ^{1}H NMR (400 MHz, $CDCl_3$) δ_H: 7.86–7.83 (m, 2H, PhC(2,6)*H*), 7.51–7.46 (m, 1H, PhC(4)*H*), 7.38–7.34 (m, 2H, PhC(3,5)*H*), 7.13 (d, *J* = 8.7 Hz, 2H, ArC(2,6)*H*), 6.71 (d, *J* = 8.7 Hz, 2H, ArC(3,5)*H*), 4.64 (d, *J* = 10.4 Hz, 1H, C(O)C*H*), 4.18 (qd, *J* = 7.2, 1.2 Hz, 2H, OCH_2), 3.90–3.74 (m, 1H, C*H*CH_3), 3.69 (s, 3H, OCH_3), 1.36 (d, *J* = 7.0 Hz, 3H, CHCH_3), 1.21 (t, *J* = 7.2 Hz, 3H, OCH_2CH_3).

Ethyl 2-benzoyl-3-(4-bromophenyl)butanoate, 22

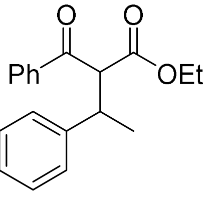

Following **General Procedure D,** 1-(4-bromophenyl)ethan-1-ol **S22** (80 mg, 0.4 mmol), ethyl benzoylacetate **17** (159 g, 0.8 mmol), pentafluorophenylboronic acid **1** (4.2 mg, 20 μmol) and oxalic acid **2** (3.6 mg, 40 μmol) in $MeNO_2$ (8.0 mL) were reacted at 90 °C for 24 h. The crude product was purified by silica-gel column chromatography (petrol/EtOAc, 95:5 to 85:15 R_f: 0.19) to give **22** as a mixture of diastereoisomers (52:48 dr, 91 mg, 61%) as a colourless oil. ν_{max} 2979 (C–H), 1742 (C=C), 1695 (C=C), 1523 (C=C),

Data for major diastereoisomer: ^{1}H NMR (300 MHz, $CDCl_3$) (*selected)* δ_H: 8.14–8.05 (m, 2H, ArC*H*), 7.54–7.46 (m, 2H, ArC*H*), 7.46–7.36 (m, 1H, ArC*H*), 7.34–7.28 (m, 2H, ArC*H*), 7.23–7.16 (m, 2H, ArC*H*), 4.58 (d, *J* = 10.8 Hz, 1H, ArCCOC*H*), 4.30–4.13 (m, 1H, C*H*CH_3), 3.90–3.75 (m, 2H, OCH_2CH_3), 1.30–1.18 (m, 3H, CHCH_3), 0.92 (t, *J* = 7.1 Hz, 3H, OCH_2CH_3); ^{13}C{^{1}H} NMR (126 MHz, $CDCl_3$) δ_C: 193.4 (Ar*C*O), 168.0 (*C*OOEt), 142.7 (Ar*C(1)*), 136.2 (Ar*C(1)*), 133.9 (Ar*C(4)*H), 133.6 (Ar*C*(3,5)H), 129.4 (Ar*C*(2,6)H), 128.9 (Ar*C*H), 128.7 (Ar*C*H), 120.4 (Ar*C*(4)Br), 61.4 (ArCCO*C*H), 46.2 (*C*H_2CH_3), 39.3 (*C*HCH_3), 20.3 (CH*C*H_3), 13.9 (CH_2*C*H_3); HRMS (ESI^-) *m/z:* $[M–H]^-$ calcd for $C_{19}H_{18}BrO_3$ 373.0445, found 373.0446.

Data for minor diastereoisomer: ^{1}H NMR (300 MHz, $CDCl_3$) (*selected)* δ_H: 7.89–7.82 (m, 2H, ArC*H*), 7.66–7.57 (m, 1H, ArC*H*), 7.54–7.47 (m, 2H, ArC*H*), 7.46–7.36 (m, 2H, ArC*H*), 7.14–7.06 (m, 2H, ArC*H*), 4.62 (d, *J* = 10.7 Hz, 1H, ArCCOC*H*), 4.30–4.13 (m, 1H, C*H*CH_3), 3.90–3.75 (m, 2H, OCH_2CH_3), 1.30–1.18 (m, 3H, OCH_2CH_3);^{13}C{^{1}H} NMR (126 MHz, $CDCl_3$) (*selected)* δ_C: 193.6 (Ar*C*O), 168.5 (*C*OOEt), 143.1 (Ar*C(1)*), 136.9 (Ar*C(1)*), 133.9 (Ar*C*(3,5)H), 129.6 (Ar*C*(2,6)H), 129.0 (Ar*C*H), 128.8 (Ar*C*H), 120.7 (Ar*C*(4)Br), 61.6 (ArCCO*C*H), 39.4 (*C*HCH_3), 20.7 (CH*C*H_3), 14.2 (CH_2*C*H_3).

Decarboxylation of 1,3-Ketoesters

General Procedure E: Decarboxylation with KOH

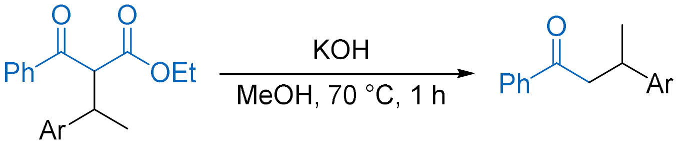

In accordance with the literature,[20] the required ethyl 3-aryl-2-benzoylbutanoate (1.0 equiv.) was dissolved in MeOH (0.5 M) before aqueous KOH (10 equiv., 4.4 M in H_2O) was added. The reaction was heated at 70 °C until complete by TLC analysis (*ca.* 1 h). The reaction was cooled to rt before CH_2Cl_2 (20 mL) was added. The solution was washed with water, brine, dried over $MgSO_4$, filtered, and concentrate under reduced pressure. The crude product was purified by silica-gel column chromatography (Petrol/EtOAc).

General Procedure F: Decarboxylation with NaOH

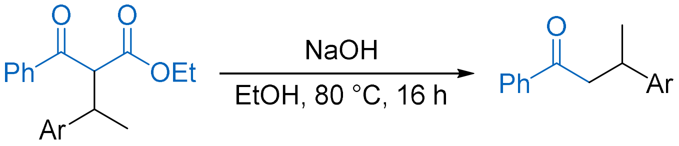

In accordance with the literature,[21] the required ethyl 3-aryl-2-benzoylbutanoate (1.0 equiv.) was dissolved in EtOH (0.75 M) before aqueous NaOH (47 equiv., 2 M in H_2O) was added. The reaction was heated at 80 °C for 16 h. The reaction was cooled to rt before being acifdifid to pH 4 using 2 M HCl. The solution was extracted with Et_2O (3×20 mL), washed with water, brine, dried over $MgSO_4$, filtered, and concentrated under reduced pressure. The crude product was purified by silica-gel column chromatography (Petrol/EtOAc).

1,3-Diphenylbutan-1-one, 23

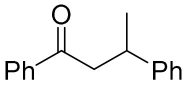

Following **General Procedure E**, ethyl 2-benzoyl-3-phenylbutanoate **19** (50 mg, 0.17 mmol) in MeOH (0.5 mL), was added KOH (100 mg, 1.7 mmol in 0.5 mL of H_2O) and was heated at reflux for 1 h. The crude product was purified by silica-gel column chromatography (petrol/EtOAc, 98:2 to 90:10 R_f: 0.31) to give title compound **23** (19 mg, 49%) as a colourless oil, with spectroscopic data in accordance with the literature.[22] mp 67–69 °C {Lit.[22] 68–69 °C}; ^{1}H NMR (300 MHz, $CDCl_3$) δ_H: 7.99–7.87 (m, 2H, ArC*H*), 7.61–7.49 (m, 1H, ArC*H*), 7.49–7.39 (m, 2H, ArC*H*), 7.36–7.26 (m, 4H, ArC*H*), 7.23–7.15 (m, 1H, ArC*H*), 3.57–3.43 (m, 1H, C*H*CH_3), 3.31 (dd, J = 16.5, 5.7 Hz, 1H, CH^AH^B), 3.19 (dd, J = 16.5, 8.3 Hz, 1H, CH^AH^B),

1.34 (d, J = 6.9 Hz, 3H, CHCH_3).

1-Phenyl-3-(*p*-tolyl)butan-1-one, 24

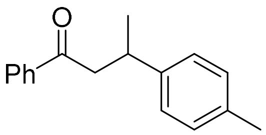

Following **General Procedure F**, ethyl 2-benzoyl-3-(*p*-tolyl)butanoate **20** (266 mg, 0.85 mmol), aqueous 2 M NaOH (4 mL) in EtOH (4 mL) was heated at reflux for 16 h. Crude product was purified by silica-gel column chromatography (petrol/EtOAc, 98:2 to 95:5 + Et_3N (1%), R_f: 0.27) to give title compound **24** (198 g, 98%) as a viscous yellow oil, with spectroscopic data in accordance with the literature.[22] ^1H NMR (300 MHz, $CDCl_3$) δ_H: 7.98–7.90 (m, 2H, ArCH), 7.60–7.51 (m, 1H, ArCH), 7.50–7.38 (m, 2H, ArCH), 7.21–7.07 (m, 4H, ArCH), 3.55–3.39 (m, 1H, CHCH$_3$), 3.28 (dd, J = 16.5, 5.7 Hz, 1H, CH^AH^B), 3.17 (dd, J = 16.5, 8.3 Hz, 1H, CH^AH^B), 2.32 (s, 3H, ArCCH_3), 1.32 (d, J = 6.9 Hz, 3H, CHCH_3).

3-(4-Methoxyphenyl)-1-phenylbutan-1-one, 25

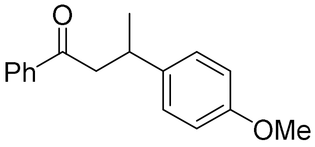

Following **General Procedure F** ethyl 2-benzoyl-3-(4-methoxy)butanoate **21** (200 mg, 0.60 mmol), aqueous 2 M NaOH (4 mL) in EtOH (4 mL) was heated at reflux for 16 h. The crude product was purified by silica-gel column chromatography (petrol/EtOAc, 98:2 to 95:5 R_f: 0.23) to give title compound **25** (99 mg, 65%) as a white powder, with spectroscopic data in accordance with the literature.[22] mp 66–68 °C {Lit.[22] 64–66 °C}; ^1H NMR (300 MHz, $CDCl_3$) δ_H: 7.98–7.88 (m, 2H, ArCH), 7.62–7.51 (m, 1H, ArCH), 7.50–7.40 (m, 2H, ArCH), 7.24–7.13 (m, 2H, ArC(2,6)H), 6.91–6.78 (m, 2H, ArC(3,5)H), 3.79 (s, 3H, OCH_3), 3.53–3.38 (m, 1H, CHCH$_3$), 3.27 (dd, J = 16.4, 6.8 Hz, 1H, CH^AH^B), 3.15 (dd, J = 16.4, 8.1 Hz, 1H, CH^AH^B), 1.31 (d, J = 6.9 Hz, 3H, CHCH_3).

Dehydrative Allylation

General Procedure G: Dehydrative allylation

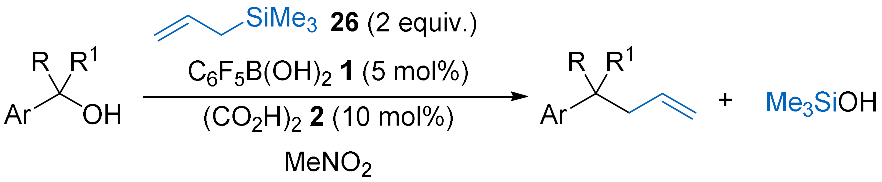

Allyltrimethylsilane **26** (2 equiv.) was added to a solution of pentafluorophenylboronic acid **1** (5 mol%) and oxalic acid **2** (10 mol%) in $MeNO_2$ (0.05 M) and was stirred at rt for 5 mins. The required benzylic alcohol (1.0 equiv.) was added and the reaction stirred at the stated temperature until complete by TLC analysis. The reaction was cooled to rt, diluted with toluene and concentrated under reduced pressure to give the crude product, which was further purified by silica-gel column chromatography.

But-3-ene-1,1-diyldibenzene, 27

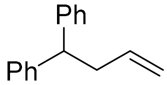

Following a modified **General Procedure G,** benzhydrol (74 mg, 0.4 mmol), allyltrimethylsilane **26** (320 µL, 2 mmol, 5 equiv.), pentafluorophenylboronic acid **1** (4.2 mg, 20 µmol) and oxalic acid **2** (3.6 mg, 40 µmol) in $MeNO_2$ (8.0 mL) were reacted at 90 °C for 16 h. The crude product was purified by silica-gel column chromatography (petrol/EtOAc, 95:5 R_f: 0.33) to give title compound **27** (80 mg, 96%) as a colourless oil, with spectroscopic data in accordance with the literature.[23] ^1H NMR (300 MHz, $CDCl_3$) δ_H: 7.36–7.15 (m, 10H, ArC*H*×2), 5.84–5.66 (m, 1H, $CH_2C\textit{H}CH_2$), 5.12–4.94 (m, 2H, $CH_2CHC\textit{H}_2$), 4.04 (t, J = 7.9 Hz, 1H, ArCC*H*), 2.90–2.79 (m, 2H, $C\textit{H}_2CH$).

1-Methyl-4-(pent-4-en-2-yl)benzene, 28

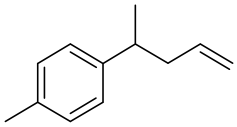

Following **General Procedure G**, 1-(*p*-tolyl)ethan-1-ol (54 mg, 0.4 mmol), allyltrimethylsilane **26** (130 µL, 0.8 mmol), pentafluorophenylboronic acid **1** (4.2 mg, 20 µmol) and oxalic acid **2** (3.6 mg, 40 µmol) in $MeNO_2$ (8.0 mL) were reacted at rt for 16 h. The crude product was purified by silica-gel column chromatography (petrol/EtOAc, 98:2 R_f: 0.31) to give title compound **28** (37 mg, 40%) as a colourless oil, with spectroscopic data in

accordance with the literature.[24] ^{1}H NMR (300 MHz, $CDCl_3$) δ_H: 7.13–7.07 (m, 4H, ArC*H*), 5.78–5.65 (m, 1H, CH_2C*H*CH_2), 5.03–4.93 (m, 2H, CH_2CHC*H_2*), 2.81–2.70 (m, 1H, CH_2C*H*), 2.32 (s, 3H, ArC*H_3*), 2.42–2.21 (m, 2H, C*H_2*CH), 1.23 (d, J = 6.9 Hz, 3H, CHC*H_3*).

1-Methoxy-4-(pent-4-en-2-yl)benzene, 29

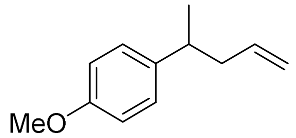

Following **General Procedure G**, 1-(4-methoxyphenyl)ethan-1-ol **S19** (61 mg, 0.4 mmol), allyltrimethylsilane **26** (130 µL, 0.8 mmol), pentafluorophenylboronic acid **1** (4.2 mg, 20 µmol) and oxalic acid **2** (3.6 mg, 40 µmol) in $MeNO_2$ (8.0 mL) were reacted at rt for 16 h. The crude product was purified by silica-gel column chromatography (petrol/EtOAc, 98:2 R_f: 0.38) to give title compound **29** (54 mg, 76%) as a colourless oil, with spectroscopic data in accordance with the literature.[24] ^{1}H NMR (300 MHz, $CDCl_3$) δ_H: 7.17–7.05 (m, 2H, ArC(2,6)*H*), 6.90–6.77 (m, 2H, ArC(3,5)*H*), 5.80–5.60 (m, 1H, CH_2C*H*CH_2), 5.06–4.89 (m, 2H, CH_2CHC*H_2*), 3.79 (s, 3H, OC*H_3*), 2.84–2.67 (m, 1H, CH_2C*H*), 2.42–2.15 (m, 2H, C*H_2*CH), 1.23 (d, J = 7.0 Hz, 3H, CHC*H_3*).

1-Fluoro-4-(pent-4-en-2-yl)benzene, 30

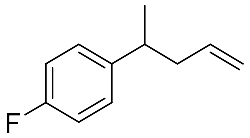

Following **General Procedure G**, 1-(4-fluorophenyl)ethan-1-ol **S21** (56 mg, 0.4 mmol), allyltrimethylsilane **26** (130 µl, 0.8 mmol), pentafluorophenylboronic acid **1** (4.2 mg, 20 µmol) and oxalic acid **2** (3.6 mg, 40 µmol) in $MeNO_2$ (8.0 mL) were reacted at rt for 16 h. The crude product was either extracted with EtOAc (3×10 mL), washed with $NaHCO_3$, brine, dried over anhydrous $MgSO_4$, filtered, and concentrated under reduced pressure. The crude material was purified by silica-gel column chromatography (petrol/EtOAc, 98:2 to 95:5 R_f: 0.42) to give title compound **30** (35 mg, 55%) as a colourless oil, with spectroscopic data in accordance with the literature.[24] ^{1}H NMR (300 MHz, $CDCl_3$) δ_H: 7.19–7.09 (m, 2H, ArC(2,6)*H*), 7.04–6.92 (m, 2H, ArC(3,5)*H*), 5.77–5.59 (m, 1H, CH_2C*H*CH_2), 5.04–4.90 (m, 2H, CH_2CHC*H_2*), 2.86–2.70 (m, 1H, C*H*CH_3), 2.40–2.20 (m, 2H, CHC*H_2*CH), 1.23 (d, J = 6.9 Hz, 3H, CHC*H_3*).

1-Bromo-4-(pent-4-en-2-yl)benzene, 31

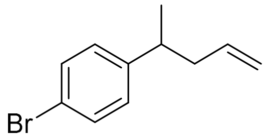

Following **General Procedure G**, 1-(4-bromophenyl)ethan-1-ol **S22** (80 mg, 0.4 mmol), allyltrimethylsilane **26** (130 μL, 0.8 mmol), pentafluorophenylboronic acid **1** (4.2 mg, 20 μmol) and oxalic acid **2** (3.6 mg, 40 μmol) in $MeNO_2$ (8.0 mL) were reacted at rt for 16 h. The crude product was purified by silica-gel column chromatography (petrol/EtOAc, 90:10 R_f: 0.36) to give title compound **31** (51 mg, 43%) as a colourless oil, with spectroscopic data in accordance with the literature.[24] ^{1}H NMR (300 MHz, $CDCl_3$) δ_H: 7.49–7.32 (m, 2H, ArC(3,5)*H*), 7.14–6.96 (m, 2H, ArC(2,6)*H*), 5.80–5.60 (m, 1H, $CH_2C\textit{H}CH_2$), 5.04–4.90 (m, 2H, $CH_2CHC\textit{H}_2$), 2.84–2.67 (m, 1H, $CH_2C\textit{H}$), 2.40–2.16 (m, 2H, $C\textit{H}_2CH$), 1.22 (d, J = 7.0 Hz, 3H, $CHC\textit{H}_3$).

1-Iodo-3-(pent-4-en-2-yl)benzene, 32

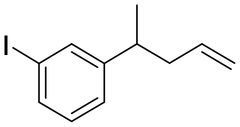

Following **General Procedure G**, 1-(3-iodophenyl)ethan-1-ol **S28** (100 mg, 0.4 mmol), allyltrimethylsilane **26** (130 μL, 0.8 mmol), pentafluorophenylboronic acid **1** (4.2 mg, 20 μmol) and oxalic acid **2** (3.6 mg, 40 μmol) in $MeNO_2$ (8.0 mL) were reacted at 90 °C for 16 h. The crude product was purified by silica-gel column chromatography (petrol/EtOAc, 95:5 R_f: 0.33) to give title compound **32** (45 mg, 41%) as a colourless oil. ν_{max} 3074 (C–H), 2961 (C–H), 1639 (C=C), 1561 (C=C); ^{1}H NMR (300 MHz, $CDCl_3$) δ_H: 7.56–7.48 (m, 2H, ArC*H*), 7.19–7.12 (m, 1H, ArC*H*), 7.07–6.97 (m, 1H, ArC*H*), 5.77–5.58 (m, 1H, $CH_2C\textit{H}CH_2$), 5.05–4.91 (m, 2H, $CH_2CHC\textit{H}_2$), 2.79–2.63 (m, 1H, $CH_2C\textit{H}$), 2.43–2.17 (m, 2H, $C\textit{H}_2CH$), 1.22 (d, J = 6.9 Hz, 3H, $CHC\textit{H}_3$); $^{13}C\{^1H\}$ NMR (126 MHz, $CDCl_3$) δ_C: 149.7 (Ar*C*(1)I), 136.7 (Ar*C*(2)I), 136.3 (*C*HCH_2), 135.2 (Ar*C*(4)I), 130.2 (Ar*C*(5)I), 126.5 (Ar*C*(6)I), 116.5 ($CH\textit{C}H_2$), 94.6 (Ar*C*(3)I), 42.6 ($CH\textit{C}H_2CH$), 39.6 (*C*HCH_3), 21.2 ($CH\textit{C}H_3$); HRMS (ESI^-) *m/z:* $[M]^-$ calcd for $C_{11}H_{13}I$ 272.0062, found 272.0052.

1-Iodo-2-(pent-4-en-2-yl)benzene, 33

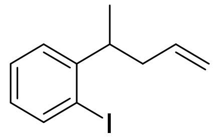

Following **General Procedure G**, 1-(2-iodophenyl)ethan-1-ol **S29** (100 mg, 0.4 mmol), allyltrimethylsilane **26** (130 µl, 0.8 mmol), pentafluorophenylboronic acid **1** (4.2 mg, 20 µmol) and oxalic acid **2** (3.6 mg, 40 µmol) in $MeNO_2$ (8.0 mL) were reacted at 90 °C for 16 h. Crude product was purified by silica-gel column chromatography (petrol/EtOAc, 95:5 R*f*: 0.34) to give title compound **33** (59 mg, 54%) as a colourless oil. ν_{max} 3084 (C–H), 2943 (C–H), 1628 (C=C), 1542 (C=C); ^{1}H NMR (300 MHz, $CDCl_3$) δ_H: 7.86–7.78 (m, 1H, ArC*H*), 7.35–7.26 (m, 1H, ArC*H*), 7.21–7.14 (m, 1H, ArC*H*), 6.94–6.83 (m, 1H, ArC*H*), 5.85–5.64 (m, 1H, $CH_2C\mathit{H}CH_2$), 5.07–4.93 (m, 2H, $CH_2CHC\mathit{H_2}$), 3.23–3.07 (m, 1H, $CH_2C\mathit{H}$), 2.47–2.33 (m, 1H, $CH^AC\mathit{H^B}CH$), 2.29–2.13 (m, 1H, $C\mathit{H^A}CH^BCH$), 1.21 (d, *J* = 6.9 Hz, 3H, $CHC\mathit{H_3}$); $^{13}C\{^1H\}$ NMR (126 MHz, $CDCl_3$) δ_C: 148.8 (Ar*C*(1)H), 139.7 (Ar*C*(3)H), 136.6 (*C*HCH*2*), 128.6 (Ar*C*(6)H), 127.9 (Ar*C*(4)H), 126.8 (Ar*C*(5)H), 116.5 (CH*CH2*), 101.7 (Ar*C*(1)I), 43.1 (*C*HCH3), 41.7 (CH*C*H2CH), 20.6 (CH*C*H3); HRMS (ESI$^-$) *m/z:* [M–H]$^-$ calcd for $C_{10}H_{13}I$ 271.0052, found 271.0042.

1-(2,2-Dimethylhex-5-en-3-yl)-4-methoxybenzene, 34

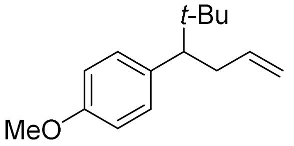

Following **General Procedure G**, 1-(4-methoxyphenyl)-2,2-dimethylpropan-1-ol **S30** (40 mg, 0.2 mmol), allyltrimethylsilane **26** (64 µL, 0.4 mmol), pentafluorophenylboronic acid **1** (2 mg, 10 µmol) and oxalic acid **2** (1 mg, 20 µmol) in $MeNO_2$ (4.0 mL) were reacted at rt for 16 h. The crude product was purified by silica-gel column chromatography (petrol/EtOAc, 95:5 to 90:10 R*f*: 0.31) to give title compound **34** (38 mg, 86%) as a colourless oil. ν_{max} 3030 (C–H), 2931 (C–H), 1698 (C=C), 1581 (C=C); ^{1}H NMR (300 MHz, $CDCl_3$) δ_H: 7.05–7.03 (m, 2H, ArC(2,6)*H*), 6.82–6.80 (m, 2H, ArC(3,5)*H*), 5.55–5.47 (m, 1H, $CH_2C\mathit{H}CH_2$), 4.93–4.78 (m, 2H, $CH_2CHC\mathit{H_2}$), 3.79 (s, 3H, $OC\mathit{H_3}$), 2.56–2.51 (m, 1H, $CH_2C\mathit{H}$), 2.45–2.36 (m, 2H, $C\mathit{H_2}CH$), 0.87 (s, 9H, $C(C\mathit{H_3})_3$); $^{13}C\{^1H\}$ NMR (126 MHz, $CDCl_3$) δ_C: 157.9 (Ar*C*(4)OMe), 138.7 (*C*HCH*2*), 134.5 (Ar*C*(1)), 130.6 (Ar*C*(2,6)H), 115.1 (CH*CH2*), 113.0 (Ar*C*(3,5)H), 56.0 (*C*HC(CH3)3), 55.3 (O*C*H3), 34.2 (*C*(CH3)3), 34.1 (CH*CH2*CH), 28.3 (C(*C*H3)3); HRMS (ESI$^-$) *m/z:* [M]$^-$ calcd for $C_{15}H_{22}O$ 218.1671, found 218.1650.

1-(Hex-5-en-1-yn-3-yl)-4-methoxybenzene, 35

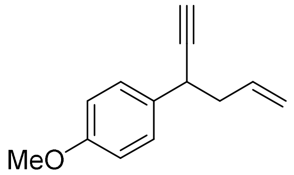

Following **General Procedure G**, 1-(4-methoxyphenyl)prop-2-yn-1-ol **S25** (32 mg, 0.2 mmol), allyltrimethylsilane **26** (64 µL, 0.4 mmol), pentafluorophenylboronic acid **1** (2 mg, 10 µmol) and oxalic acid **2** (1 mg, 20 µmol) in MeNO$_2$ (4.0 mL) were reacted at rt for 16 h. The crude product was purified by silica-gel column chromatography (petrol/EtOAc, 95:5 to 90:10 R*f*: 0.31) to give title compound **35** (32 mg, 86%) as a colourless oil, with spectroscopic data in accordance with the literature.[25] ^{1}H NMR (300 MHz, CDCl$_3$) δ_H: 7.30–7.24 (m, 2H, ArC(2,6)*H*), 6.92–6.82 (m, 2H, ArC(3,5)*H*), 5.94–5.75 (m, 1H, CH$_2$C*H*CH$_2$), 5.15–4.99 (m, 2H, CH$_2$CHC*H*$_2$), 3.80 (s, 3H, OC*H*$_3$), 3.71–3.60 (m, 1H, CH$_2$C*H*), 2.54–2.44 (m, 2H, C*H*$_2$CH), 2.29 (d, *J* = 2.5 Hz, 1H, CC*H*).

1-(Hepta-1,6-dien-4-yl)-4-methoxybenzene, 36

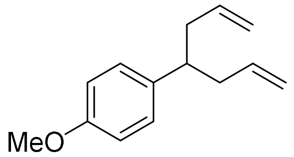

Following **General Procedure G**, 1-(4-methoxyphenyl)but-3-en-1-ol (36 mg, 0.2 mmol), allyltrimethylsilane **26** (64 µL, 0.4 mmol), pentafluorophenylboronic acid **1** (2 mg, 10 µmol) and oxalic acid **2** (1 mg, 20 µmol) in MeNO$_2$ (4.0 mL) were reacted at rt for 5 h. The crude product was purified by silica-gel column chromatography (petrol/EtOAc, 95:5 R*f*: 0.38) to give title compound **36** (37 mg, 91%) as a colourless oil, with spectroscopic data in accordance with the literature.[26] ^{1}H NMR (300 MHz, CDCl$_3$) δ_H: 7.12–6.97 (m, 2H, ArC(2,6)*H*), 6.89–6.75 (m, 2H, ArC(3,5)*H*), 5.74–5.53 (m, 2H, CH$_2$C*H*CH$_2$), 5.11–4.80 (m, 4H, CH$_2$CHC*H*$_2$), 3.78 (s, 3H, OC*H*$_3$), 2.63–2.58 (m, 1H, CH$_2$C*H*), 2.45–2.20 (m, 4H, C*H*$_2$CH and C*H*$_2$CH).

1-Methoxy-4-(2-methylpent-4-en-2-yl)benzene, 37

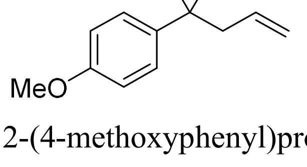

Following **General Procedure G**, 2-(4-methoxyphenyl)propan-2-ol **S31** (33 mg, 0.2 mmol), allyltrimethylsilane **26** (64 µL, 0.4 mmol), pentafluorophenylboronic acid **1** (2 mg, 10 µmol) and oxalic acid **2** (1 mg, 20 µmol) in MeNO$_2$ (4.0 mL) were reacted at rt for 16 h. The crude

product was purified by silica-gel column chromatography (petrol/EtOAc, 95:5 R_f: 0.29) to give title compound **37** (26 mg, 68%) as a colourless oil, with spectroscopic data in accordance with the literature.[27] ^{1}H NMR (300 MHz, $CDCl_3$) δ_H: 7.27–7.25 (m, 2H, ArC(2,6)*H*), 6.86–6.83 (m, 2H, ArC(3,5)*H*), 5.64–5.48 (m, 1H, $CH_2C\mathit{H}CH_2$), 4.99–4.93 (m, 2H, $CH_2CHC\mathit{H_2}$), 3.80 (s, 3H, $OC\mathit{H_3}$), 2.33 (t, J = 7.3 Hz, 2H, $C\mathit{H_2}CH$), 1.28 (s, 6H, $C(C\mathit{H_3})_2$).

1-Methoxy-4-(3-phenylpent-4-en-2-yl)benzene, 38

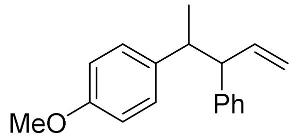

Cinnamyltrimethylsilane **S33** (76 mg, 0.4 mmol) was added to a solution of pentafluorophenylboronic acid **1** (4.2 mg, 20 µmol) and oxalic acid **2** (3.6 mg, 40 µmol) in $MeNO_2$ (8.0 mL) were stirred at rt for 5 mins. 1-(4-Methoxyphenyl)ethan-1-ol **S19** (30 mg, 0.2 mmol) was added and the reaction reacted at rt for 16 h. The reaction was diluted with toluene and concentrated under reduced pressure to give the crude product, which was further purified by silica-gel column chromatography (Petrol/EtOAc, 99:1 to 95:5 R_f:0.23) to give **38** as a mixture of diastereoisomers (64:36 dr, 73 mg, 77%) as a colourless oil. ν_{max} 3093 (C–H), 2968 (C–H), 1629 (C=C), 1537 (C=C),

Data for major diastereoisomer: ^{1}H NMR (300 MHz, $CDCl_3$) (*selected*) δ_H: 7.38–7.27 (m, 1H, ArC*H*), 7.25–7.03 (m, 4H, ArC*H*), 7.03–6.96 (m, 2H, ArC*H*), 6.95–6.88 (m, 2H, ArC*H*), 6.08–5.99 (m, 1H, $C\mathit{H}CH_2$), 5.15–5.02 (m, 2H, $CHC\mathit{H_2}$), 3.72 (s, 3H, $OC\mathit{H_3}$), 3.41–3.30 (m, 1H, PhC*H*CH), 3.09–2.94 (m, 1H, $C\mathit{H}CH_3$), 1.30 (d, J = 7.0 Hz, 3H, $CHC\mathit{H_3}$); $^{13}C\{^1H\}$ NMR (126 MHz, $CDCl_3$) δ_C: 157.7 (Ar*C*(4)OMe), 143.6 (Ar*C*(1)), 140.8 ($\mathit{C}HCH_2$), 137.5 (Ar*C*H), 128.2 (Ar*C*H×2), 126.4 (Ar*C*(2,6)H), 125.9 (Ar*C*H), 115.5 ($CH\mathit{C}H_2$), 113.4 (Ar*C*(3,5)H), 57.7 ($O\mathit{C}H_3$), 55.2 ($\mathit{C}HCH_2CH$), 44.2 ($\mathit{C}HCH_3$), 20.5 ($CH\mathit{C}H_3$); HRMS (ESI$^-$) *m/z:* [M+CH_3COO^-] calcd for $C_{20}H_{23}O_3$ 311.1653, found 311.1656.

Data for minor diastereoisomer: ^{1}H NMR (300 MHz, $CDCl_3$) (*selected*) δ_H: 7.38–7.27 (m, 1H, ArC*H*), 7.25–7.03 (m, 4H, ArC*H*), 6.87–6.80 (m, 2H, ArC*H*), 6.72–6.64 (m, 2H, ArC*H*), 5.92–5.77 (m, 1H, $C\mathit{H}CH_2$), 4.90–4.68 (m, 2H, $CHC\mathit{H_2}$), 3.80 (s, 3H, $OC\mathit{H_3}$), 3.41–3.30 (m, 1H, PhC*H*CH), 3.09–2.94 (m, 1H, $C\mathit{H}CH_3$), 1.06 (d, J = 6.9 Hz, 3H, $CHC\mathit{H_3}$); $^{13}C\{^1H\}$ NMR (126 MHz, $CDCl_3$) δ_C: 158.0 (Ar*C*(4)OMe), 128.8 (Ar*C*H×2), 126.8 (Ar*C*(2,6)H), 115.6 ($CH\mathit{C}H_2$), 113.6 (Ar*C*(3,5)H), 58.5 ($O\mathit{C}H_3$), 55.3 ($\mathit{C}HCH_2CH$), 44.7 ($\mathit{C}HCH_3$), 21.0 ($CH\mathit{C}H_3$).

Control Reactions

1,3-Diphenyl-2-[(2*E*)-3-phenylprop-2-en-1-yl]propane-1,3-dione, S13, and 1,3-Diphenyl-2-(1-phenylallyl)propane-1,3-dione, S14

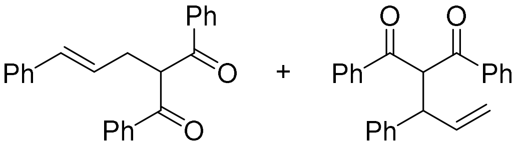

Following **General Procedure C,** cinnamyl alcohol **S12** (54 mg, 0.4 mmol), 1,3-diphenylpropane-1,3-dione (180 mg, 0.8 mmol), pentafluorophenylboronic acid **1** (4.2 mg, 20 μmol) and oxalic acid **2** (3.6 mg, 40 μmol) in $MeNO_2$ (8.0 mL) were reacted at 90 °C for 24 h. The crude product was purified by silica-gel column chromatography (petrol/EtOAc, 95:5 to 85:15 R_f: 0.21) to give an 87:13 mixture of **S13**:**S14** (87 mg, 64%) as a yellow solid, with spectroscopic data in accordance with the literature.[16]

S13 (*major*): ^{1}H NMR (400 MHz, $CDCl_3$) δ_H: 7.99–7.95 (m, 4H, ArC*H*), 7.59–7.54 (m, 2H, ArC*H*), 7.47–7.43 (m, 4H, ArC*H*), 7.32–7.23 (m, 5H, ArC*H*), 6.46 (d, *J* = 15.9 Hz, 1H, PhC*H*=CH), 6.28–6.21 (m, 1H, PhCH=C*H*), 5.35 (t, *J* = 6.8 Hz, 1H C(O)C*H*), 3.02 (td, *J* = 7.1, 1.1 Hz, 2H, CH_2CH).

S14 (*minor*): ^{1}H NMR (400 MHz, $CDCl_3$) δ_H: 8.04–8.02 (m, 2H, ArC*H*), 7.79–7.75 (m, 2H, ArC*H*), 7.21–7.16 (m, 8H, ArC*H*), 7.11–6.98 (m, 3H, ArC*H*), 6.06–5.97 (m, 1H, C*H*=CH_2), 5.88 (d, *J* = 10.6 Hz, 1H, C(O)C*H*), 5.03–4.92 (m, 2H, CH=CH_2), 4.66 (dd, *J* = 10.6, 8.0 Hz, 1H, PhCC*H*).

The reaction was also performed using vinylbenzyl alcohol **S11** (54 mg, 0.4 mmol), giving an 85:15 mixture of **S13**:**S14** in 66% yield, with spectroscopic data as above.

(*E*)-Hexa-1,5-dien-1-ylbenzene, S15

Following **General Procedure G**, cinnamyl alcohol **S12** (54 mg, 0.4 mmol), allyltrimethylsilane **26** (130 μl, 0.8 mmol), pentafluorophenylboronic acid **1** (4.2 mg, 20 μmol) and oxalic acid **2** (3.6 mg, 40 μmol) in $MeNO_2$ (8.0 mL) were reacted at rt for 16 h. ^{1}H NMR catalysis of the crude reaction mixture showed 100% conversion. The crude product was purified by silica-gel column chromatography (petrol/EtOAc, 100:0 to 90:10 R_f: 0.36) to give title compound **S15** (57 mg, 66%) as a colourless oil, with spectroscopic data in accordance with the literature.[28] ^{1}H NMR (300 MHz, $CDCl_3$) δ_H: 7.38–7.28 (m, 4H, ArC*H*), 7.24–7.12 (m, 1H, ArC(4)*H*), 6.47–6.35 (m, 1H, ArCC*H*), 6.31–6.15 (m, 1H, CHC*H*CH_2), 5.96–5.78 (m, 1H,

$CH_2C\mathit{H}CH_2$), 5.13–4.95 (m, 2H, $CH_2CHC\mathit{H_2}$), 2.38–2.18 (m, 4H, $C\mathit{H_2}C\mathit{H_2}CH$).

The reaction was also performed using vinylbenzyl alcohol **S11** (54 mg, 0.4 mmol), giving **S15** in 63% yield, with spectroscopic data as above.

NMR Spectra

3 ^{1}H NMR (500 MHz, $CDCl_3$)

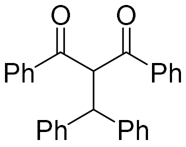

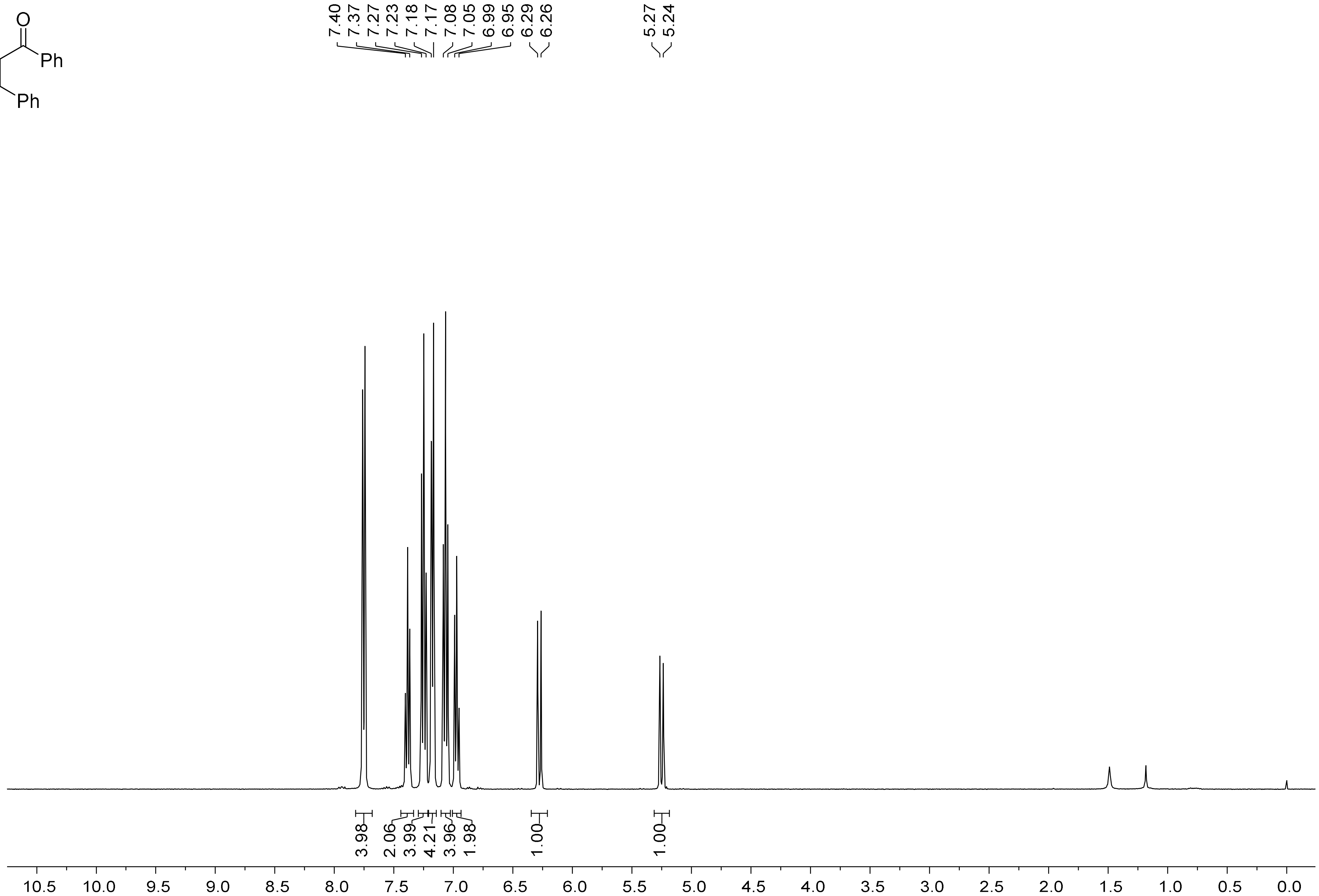

4 ^{1}H NMR (500 MHz, $CDCl_3$)

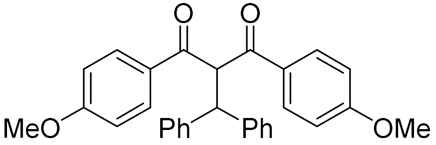

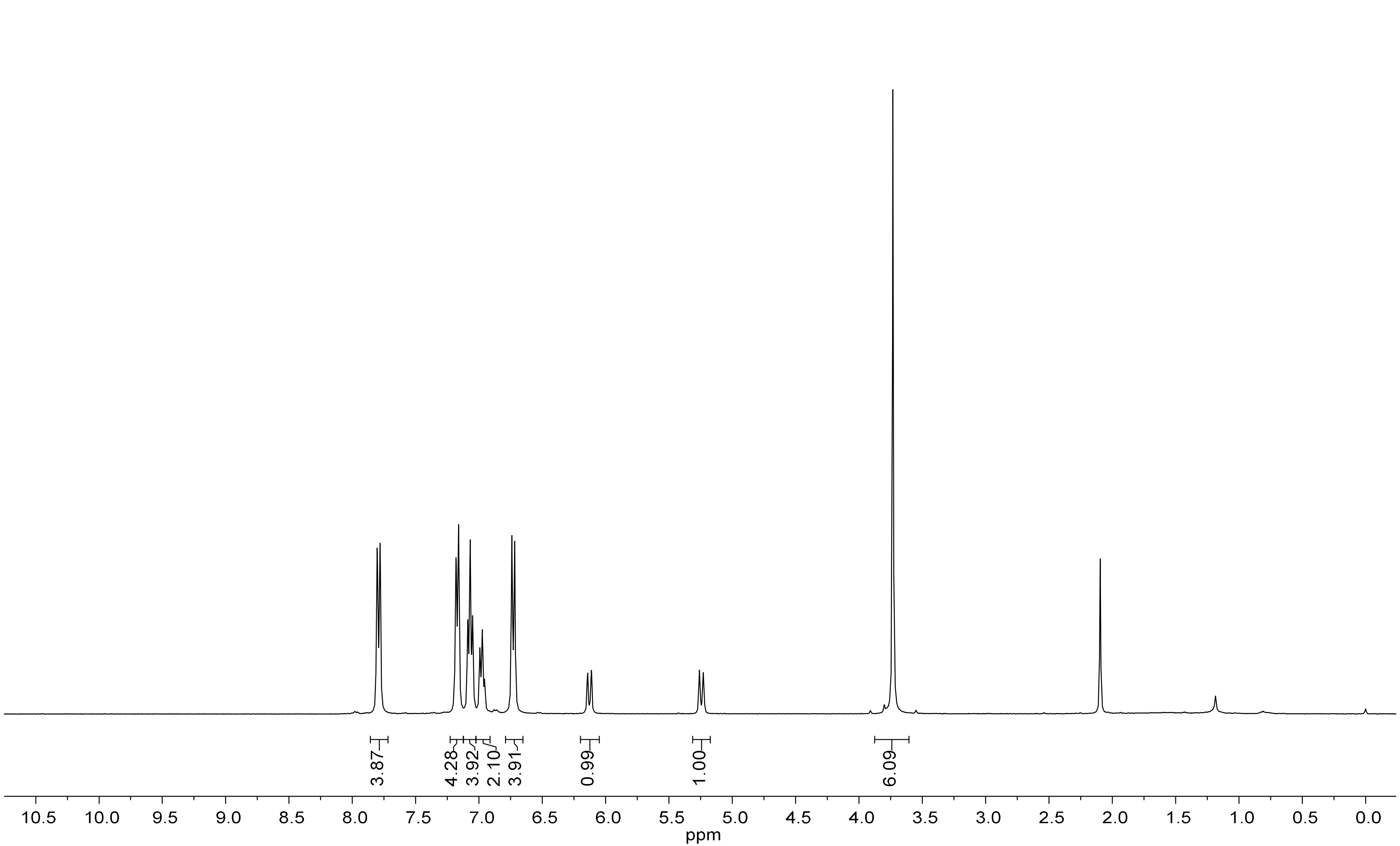

5 ^{1}H NMR ($CDCl_3$, 500 MHz)

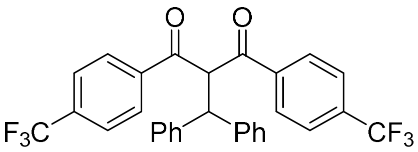

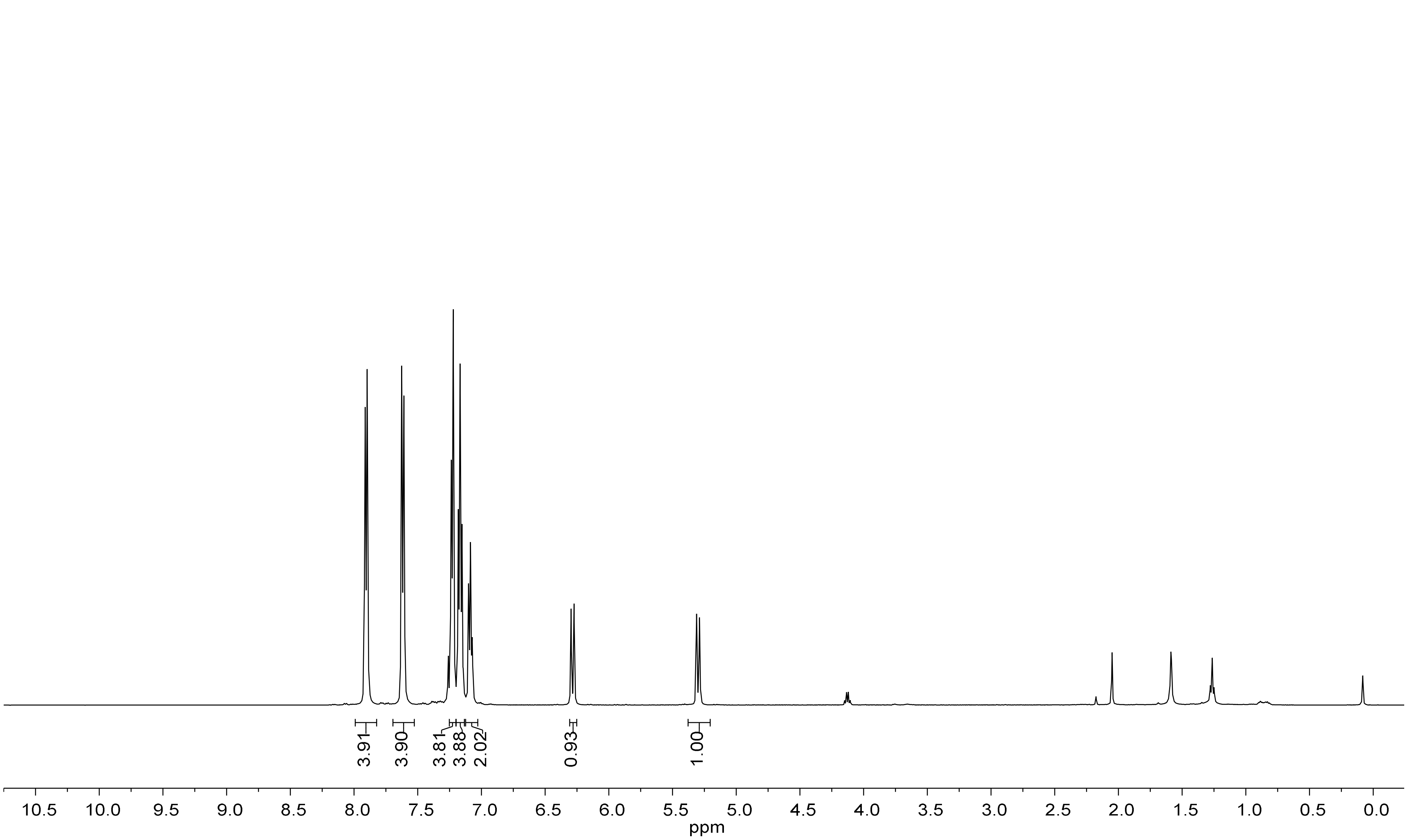

5 $^{13}C\{^{1}H\}$ NMR (126 MHz, $CDCl_3$)

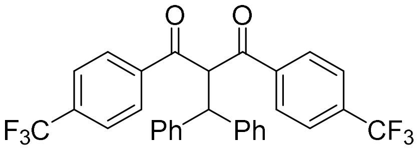

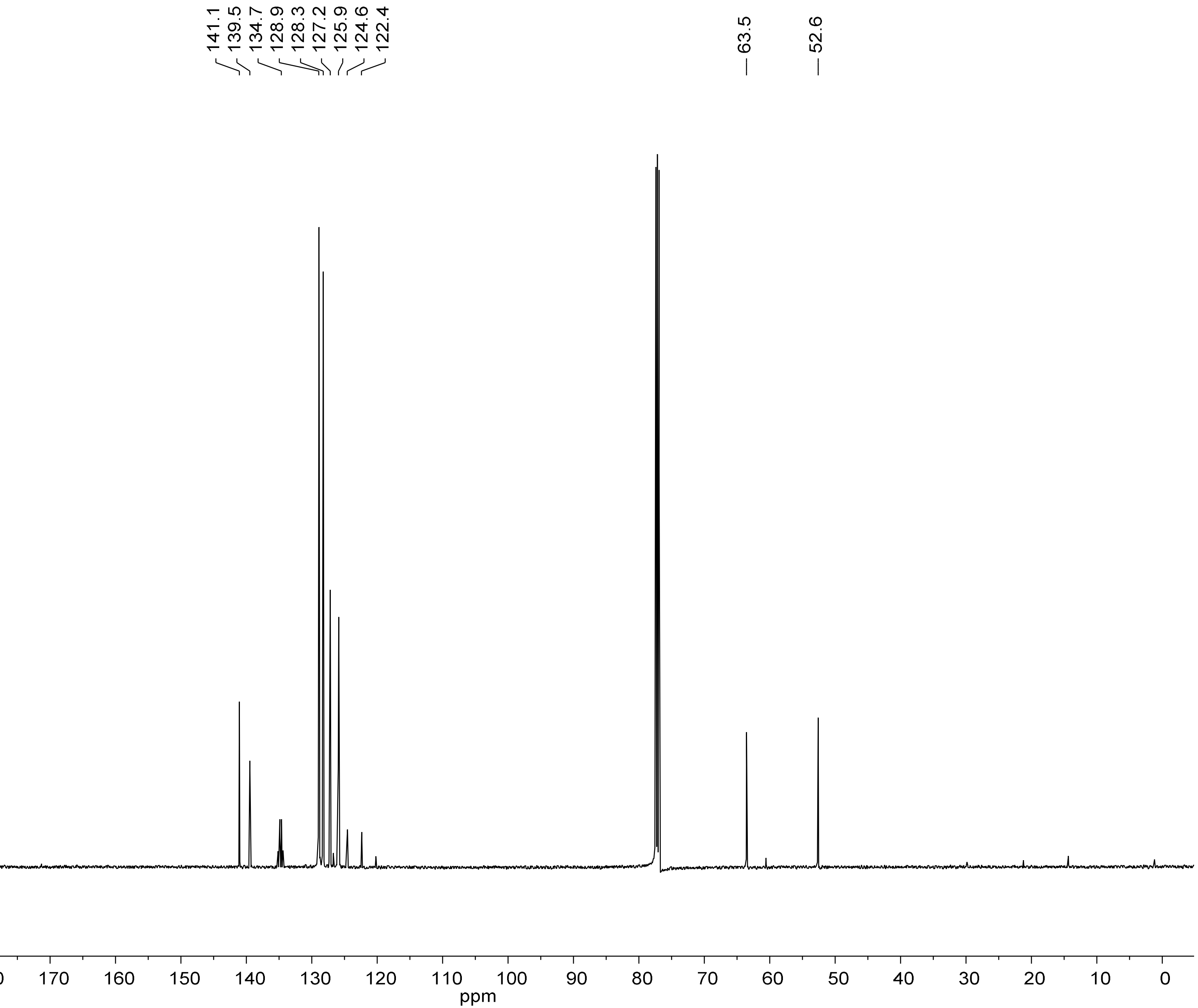

5 $^{19}F\{^{1}H\}$ NMR (471 MHz, $CDCl_3$)

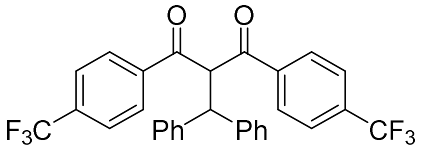

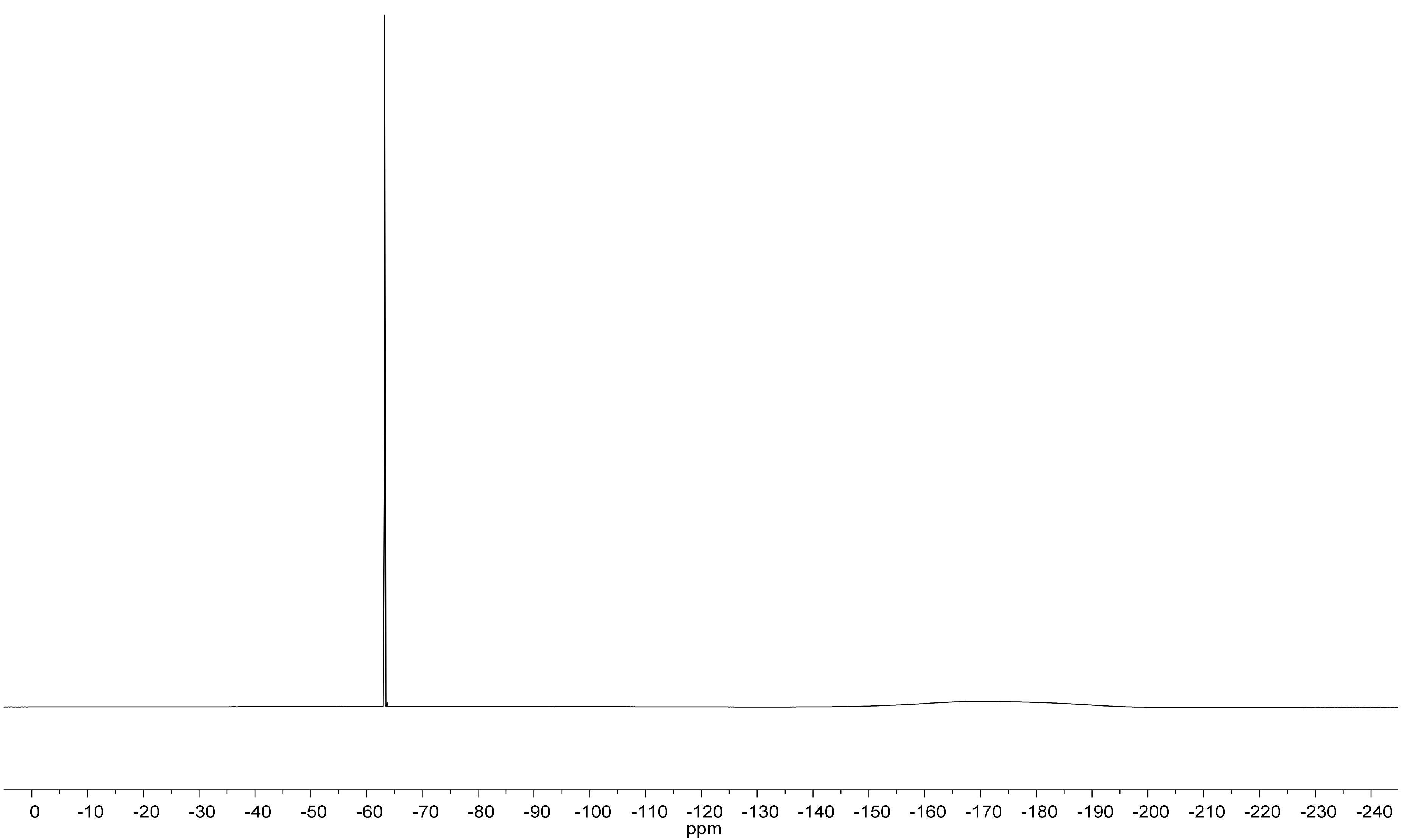

6 ^{1}H NMR ($CDCl_3$, 500 MHz)

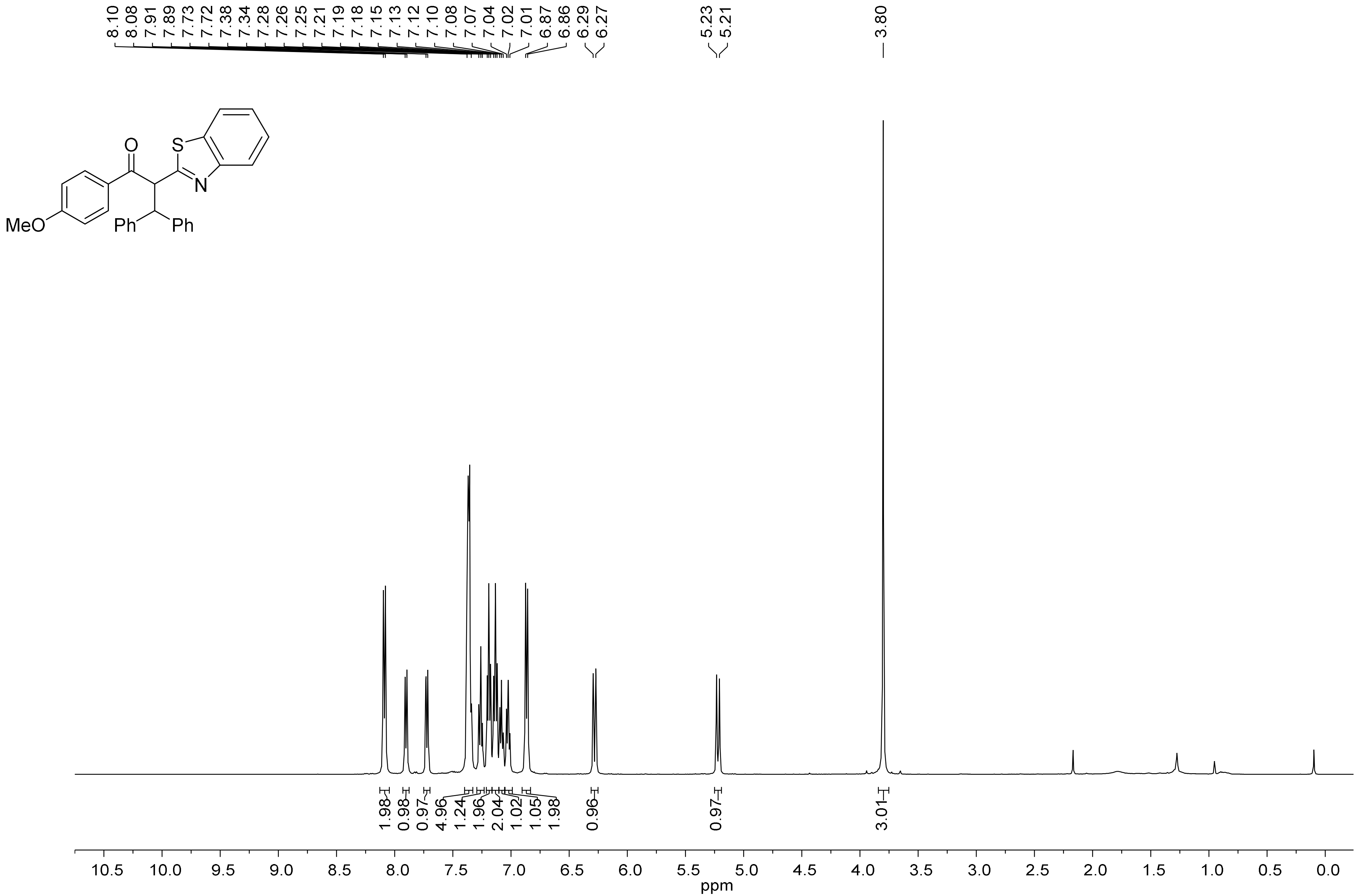

6 $^{13}C\{^1H\}$ NMR (126 MHz, $CDCl_3$)

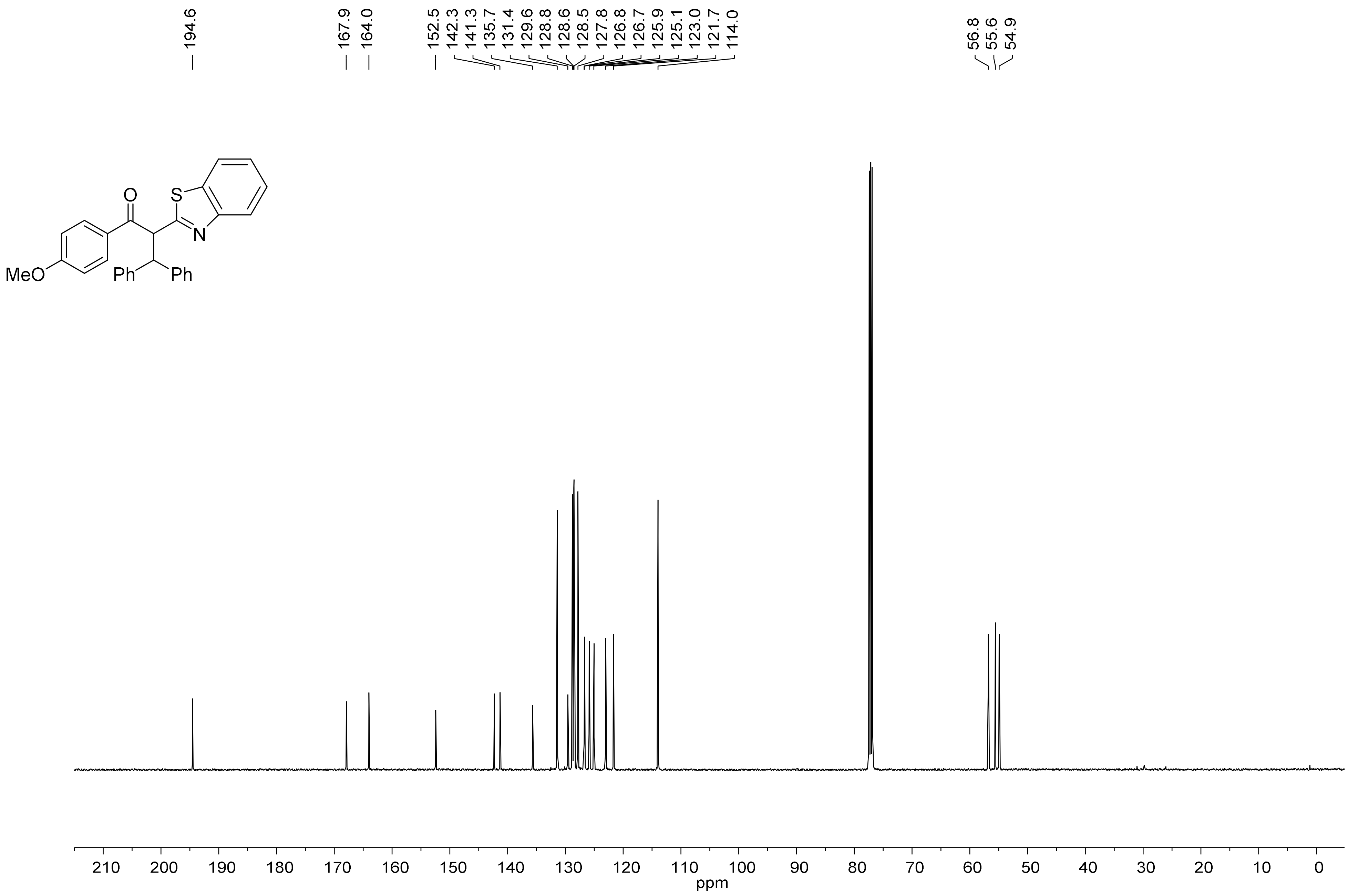

7 ^1H NMR (400 MHz, $CDCl_3$)

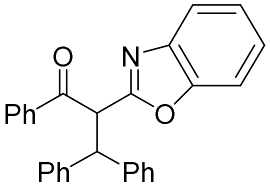

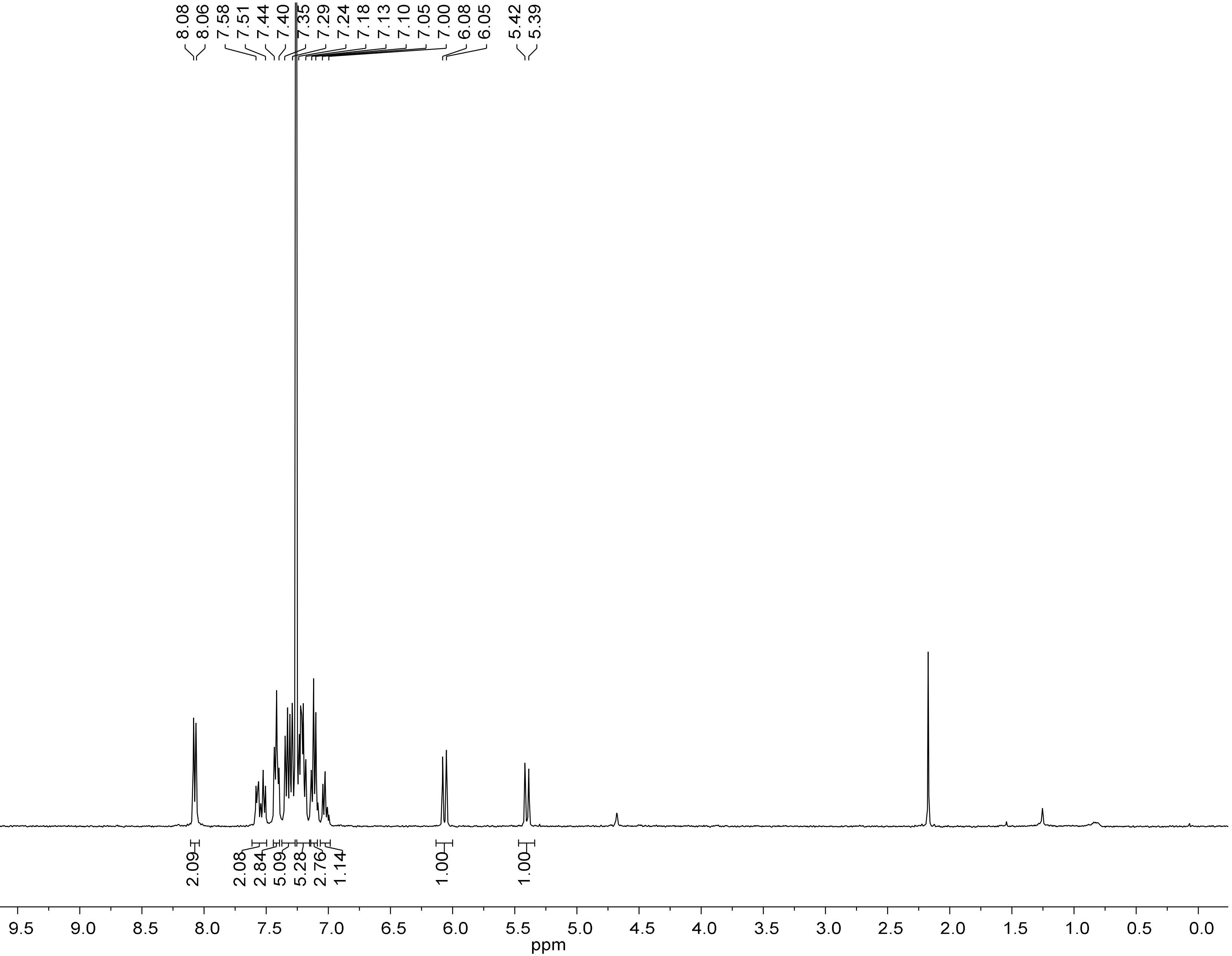

7 $^{13}C\{^{1}H\}$ NMR (126 MHz, $CDCl_3$)

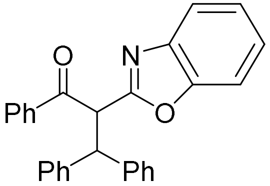

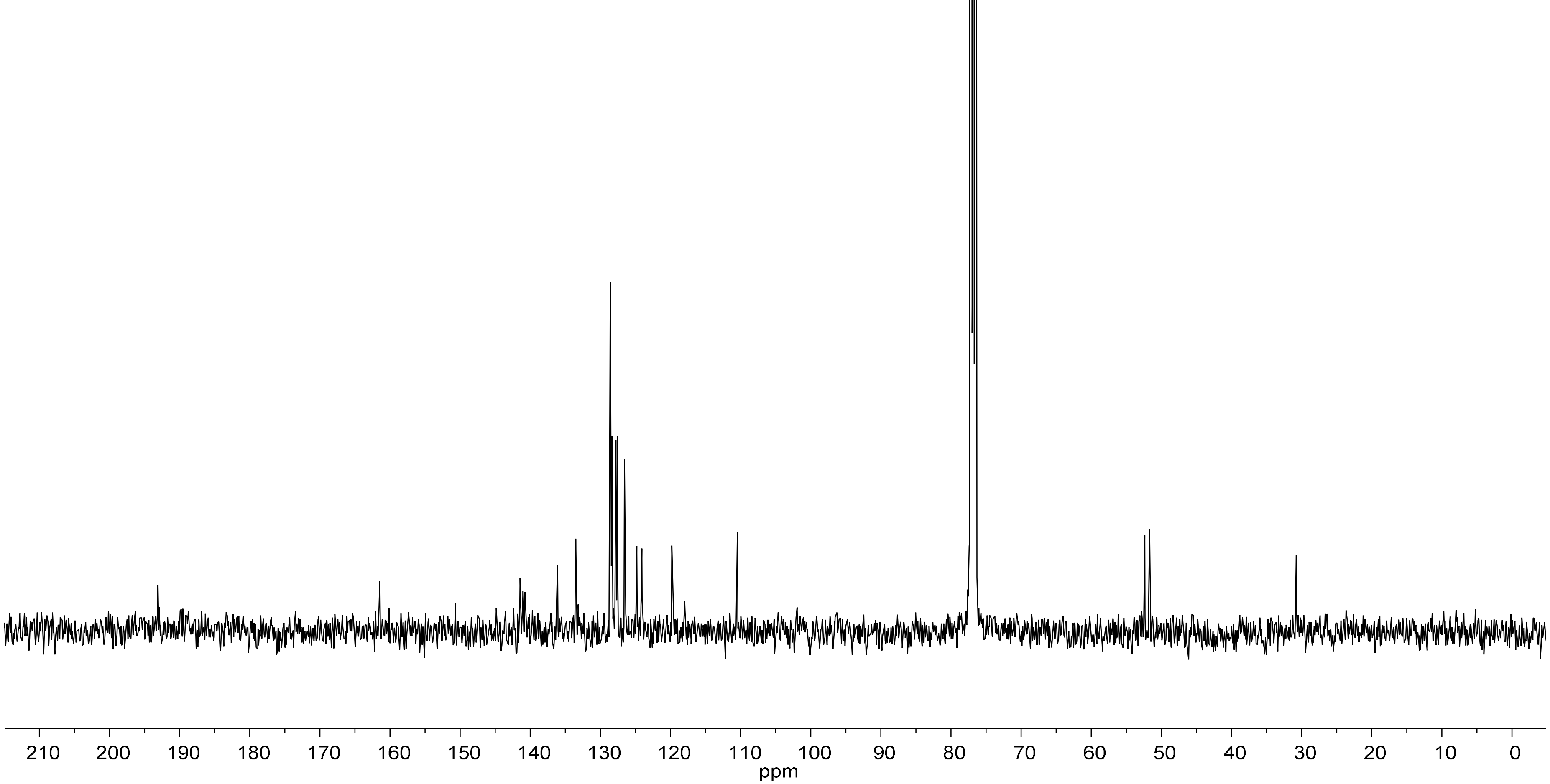

8 ^{1}H NMR (300 MHz, $CDCl_3$)

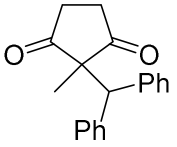

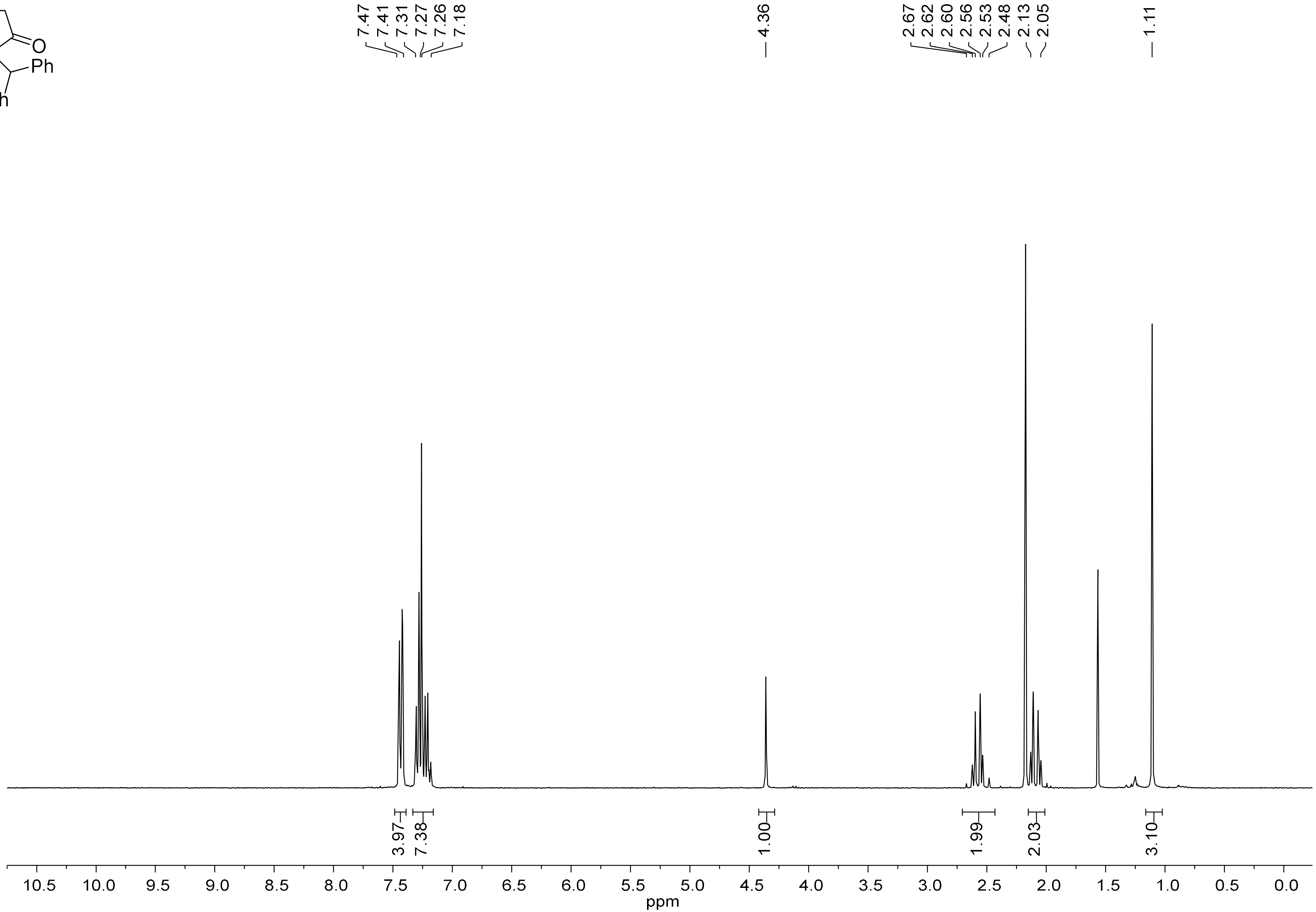

8 $^{13}C\{^1H\}$ NMR (126 MHz, $CDCl_3$)

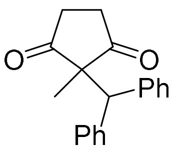

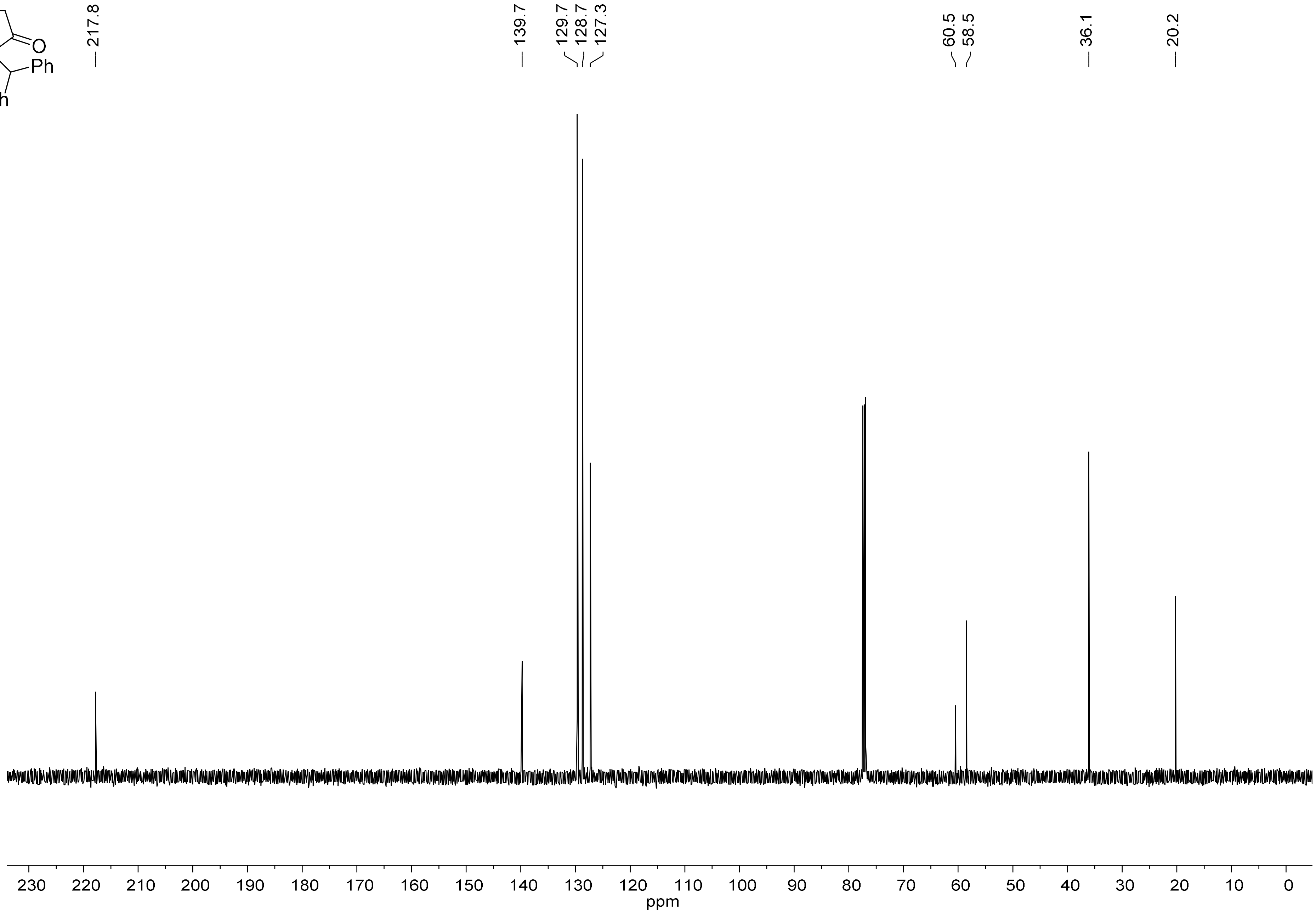

9 ^{1}H NMR (400 MHz, $CDCl_3$)

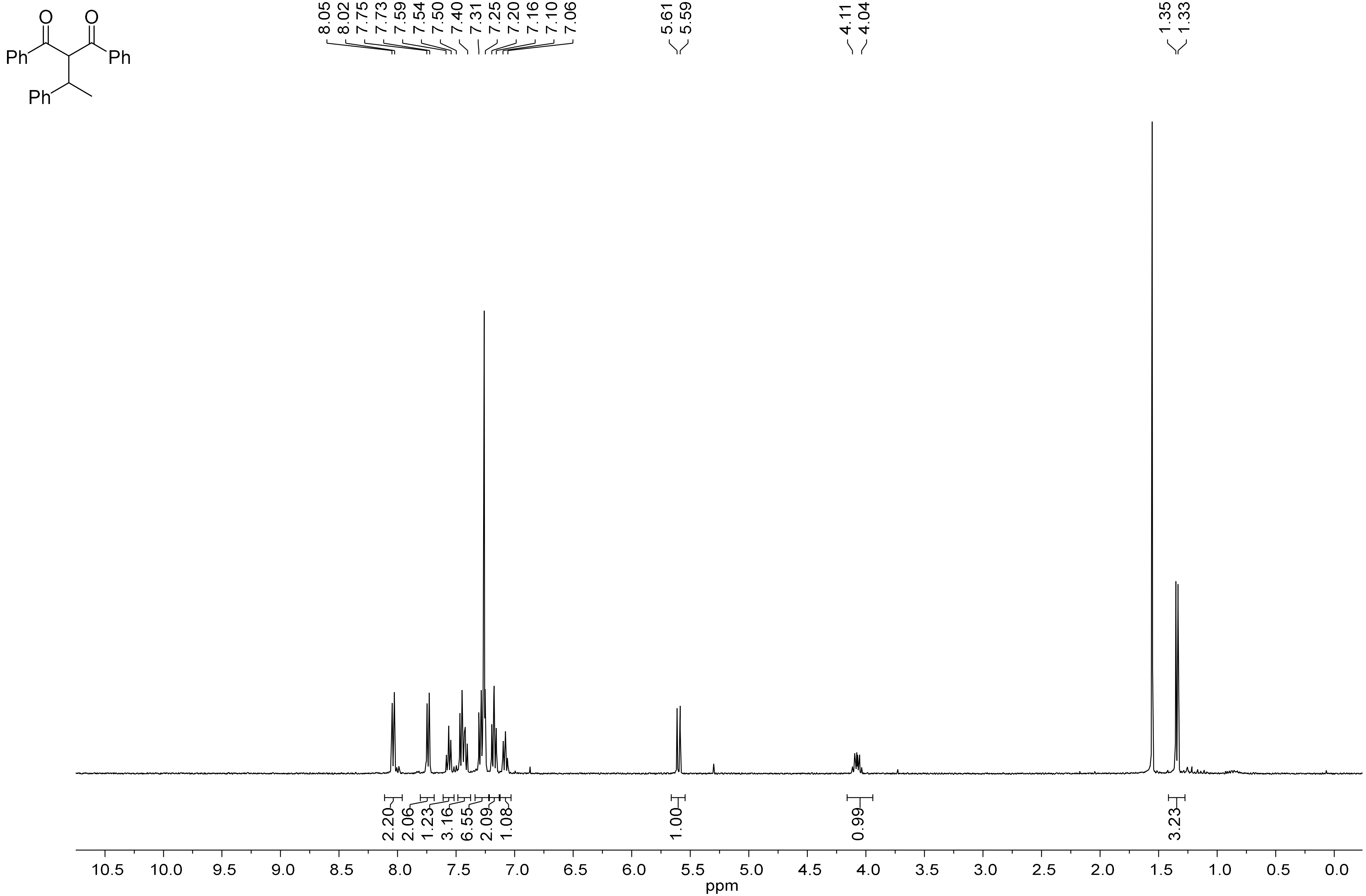

10 ^{1}H NMR (400 MHz, $CDCl_3$)

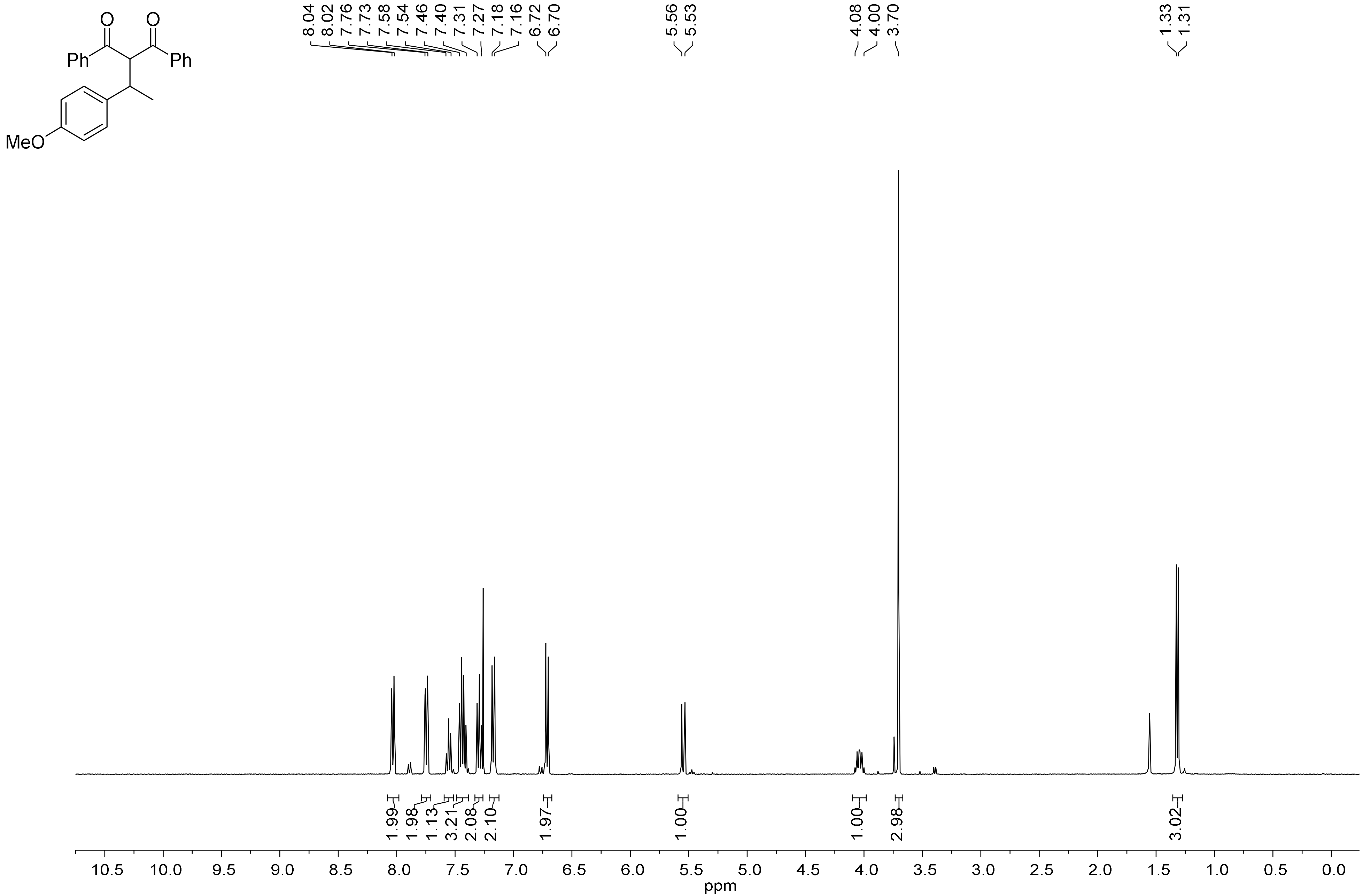

11 ^{1}H NMR (400 MHz, $CDCl_3$)

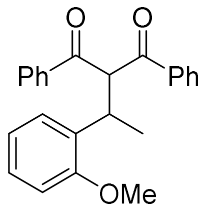

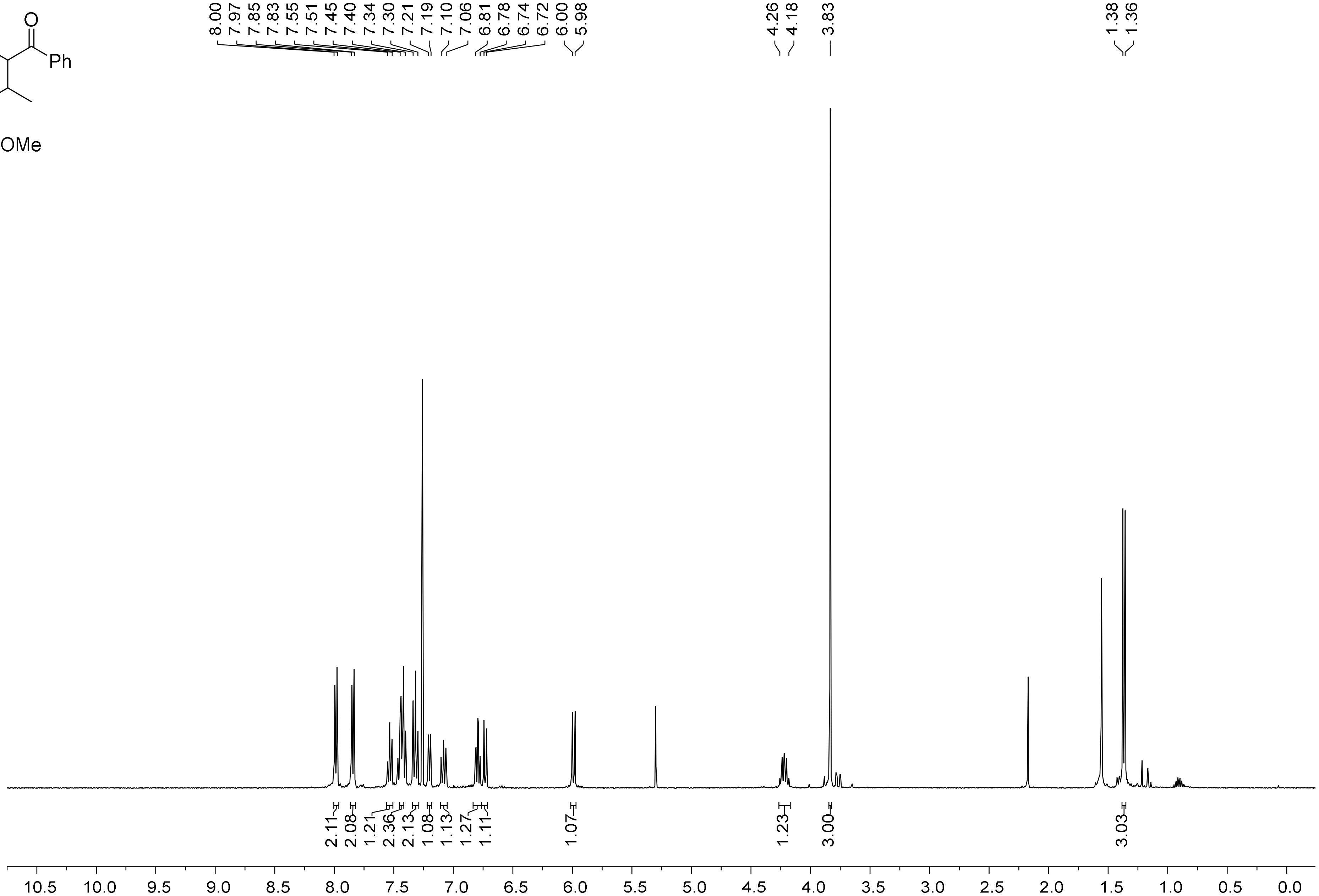

11 $^{13}C\{^{1}H\}$ NMR (126 MHz, $CDCl_3$)

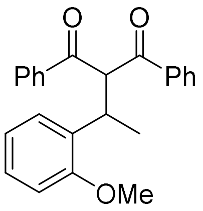

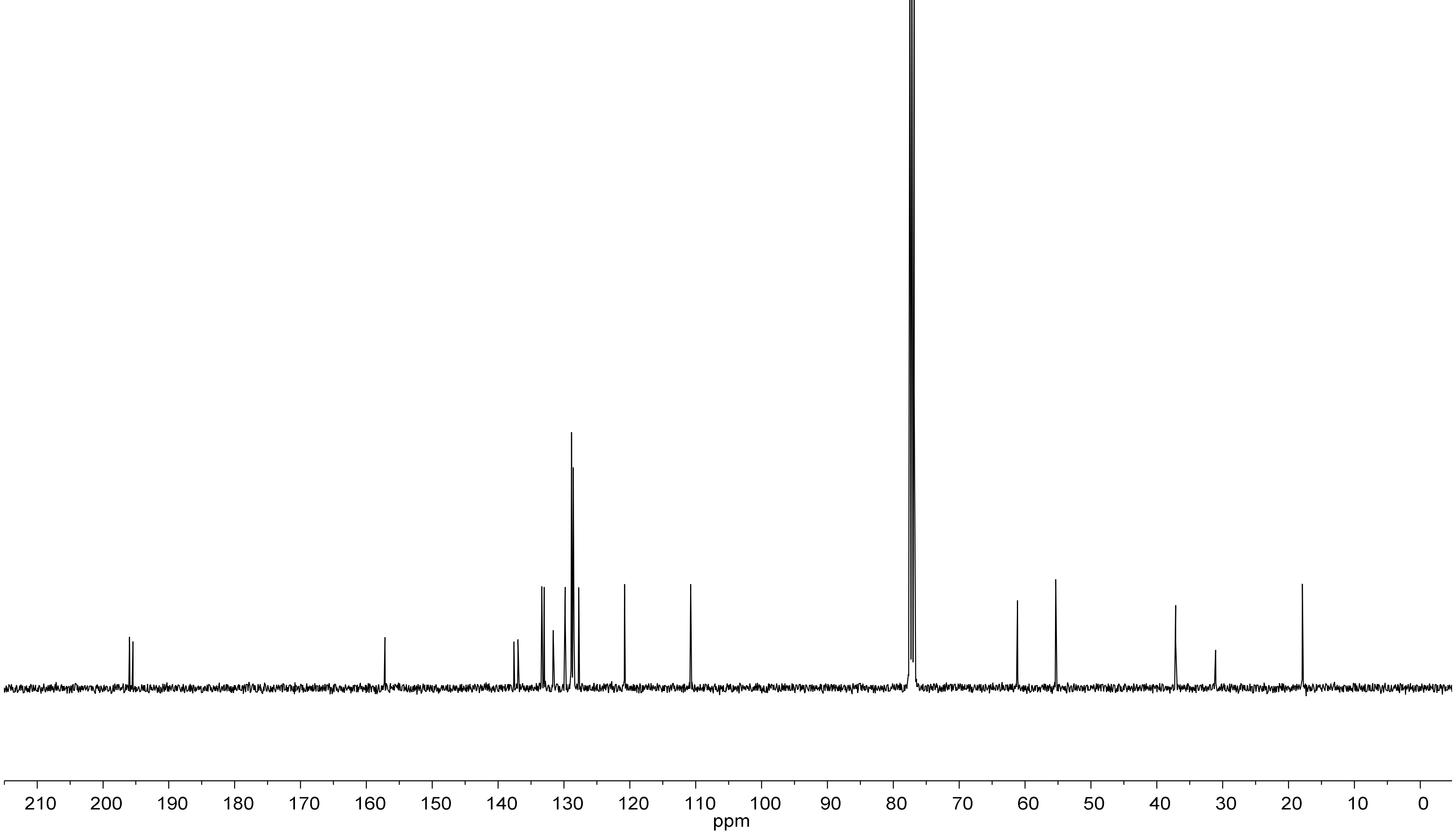

12 ^{1}H NMR (400 MHz, $CDCl_3$)

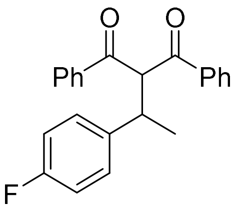

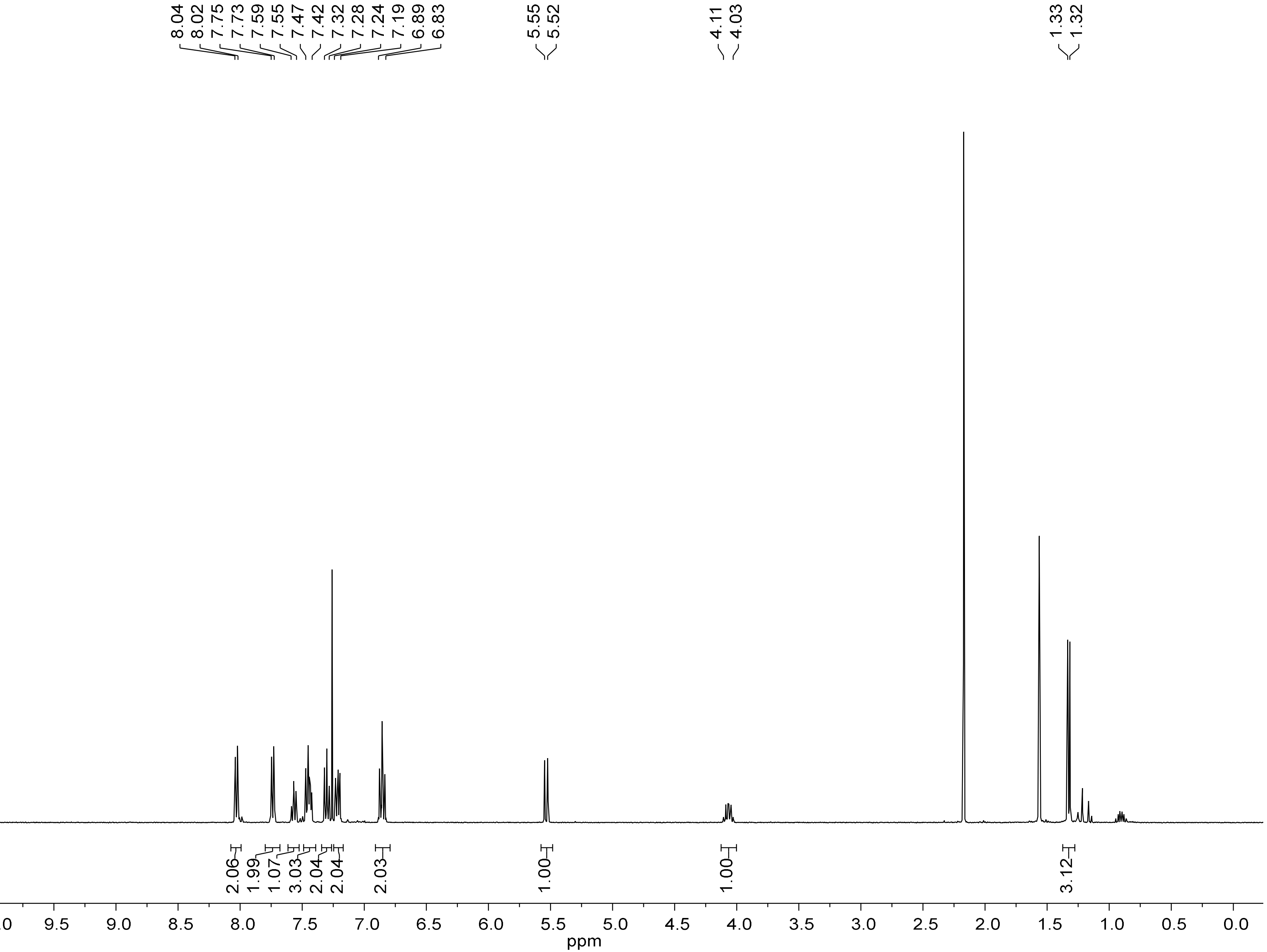

13 ^{1}H NMR (400 MHz, $CDCl_3$)

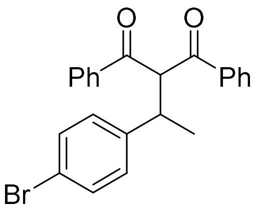

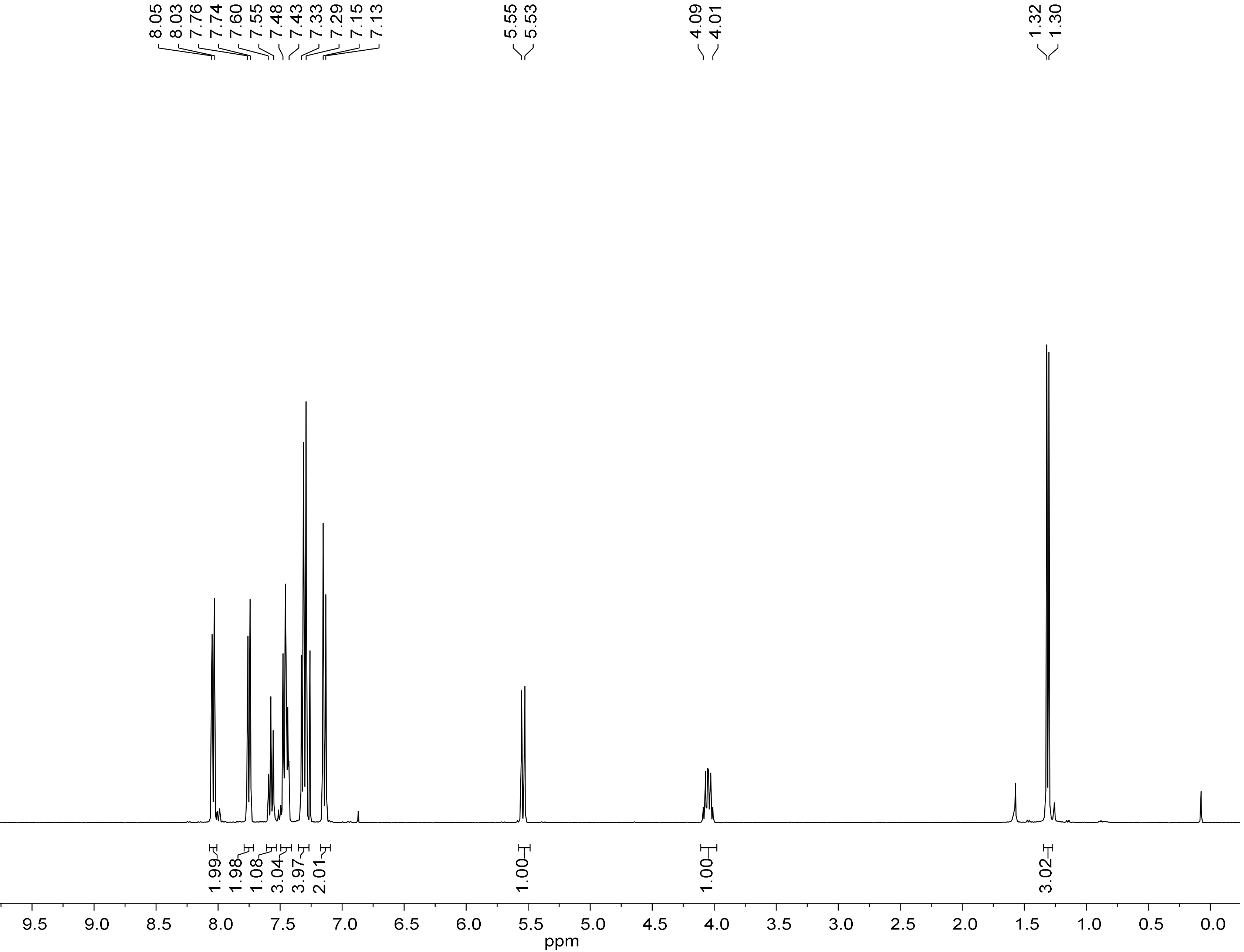

14 ^{1}H NMR (300 MHz, $CDCl_3$)

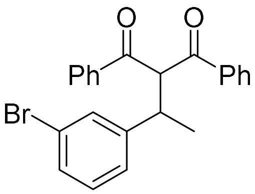

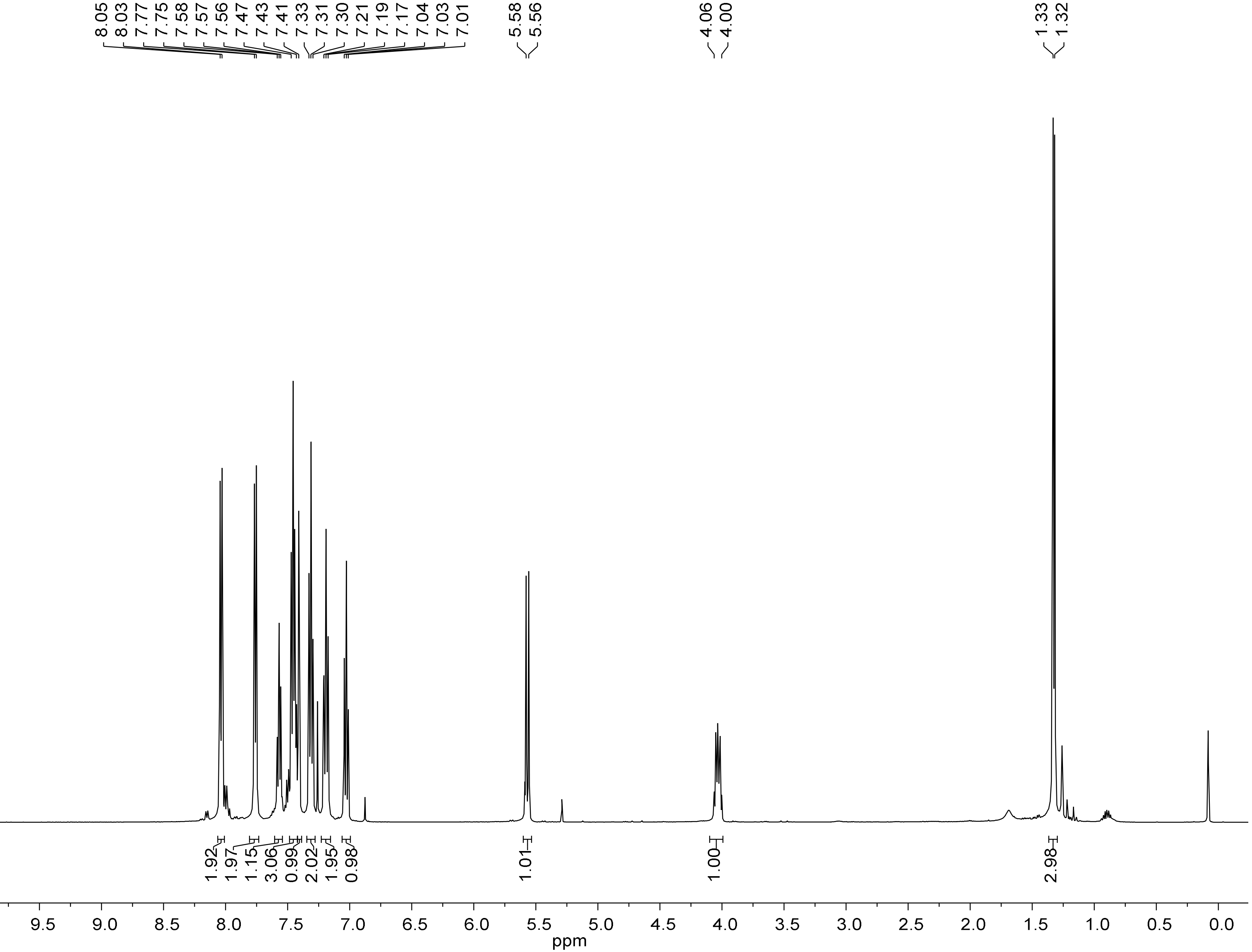

14 $^{13}C\{^{1}H\}$ NMR (126 MHz, $CDCl_3$)

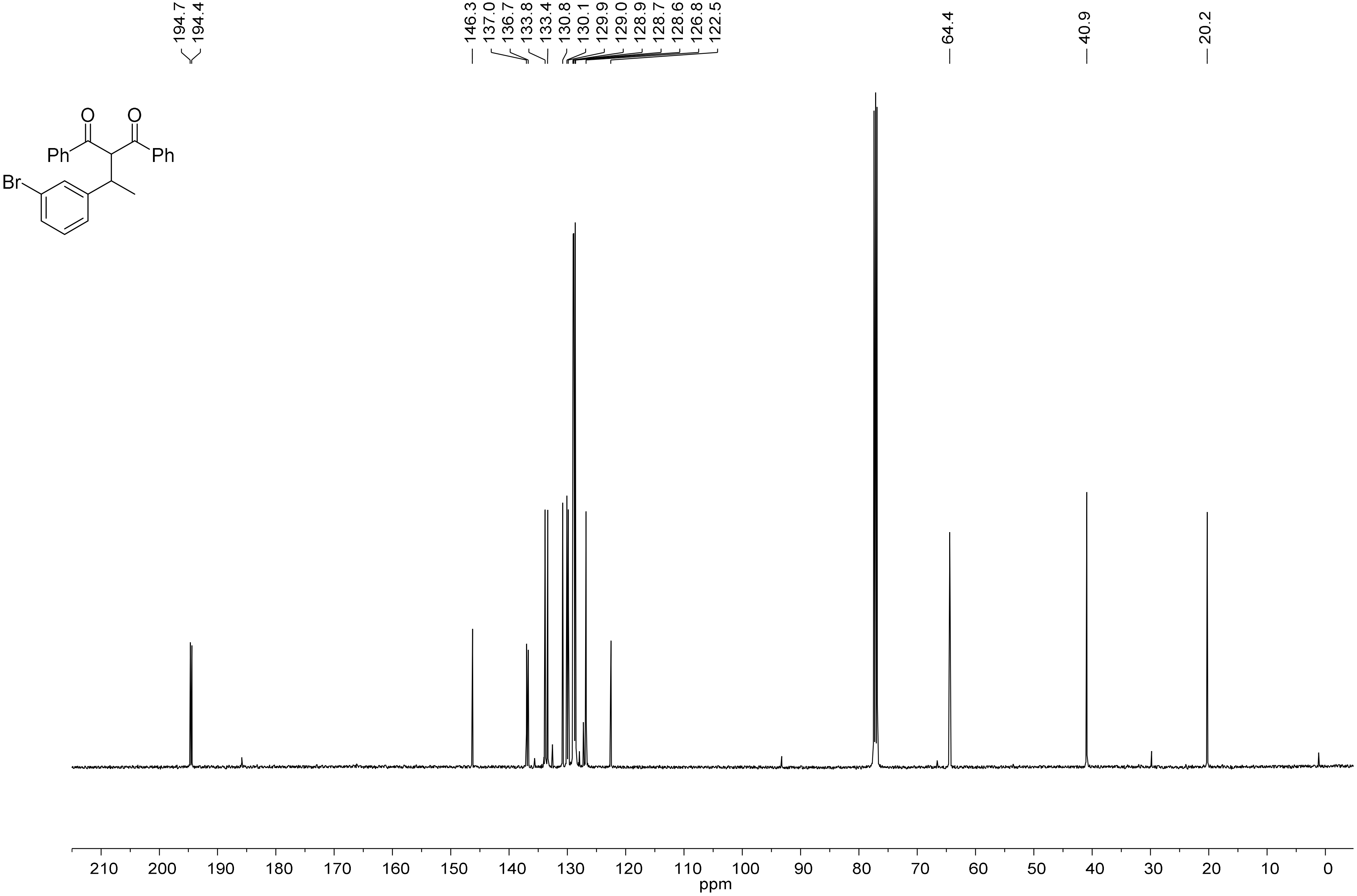

15 ^{1}H NMR (300 MHz, $CDCl_3$)

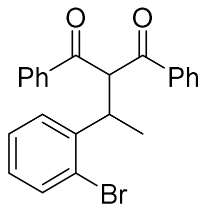

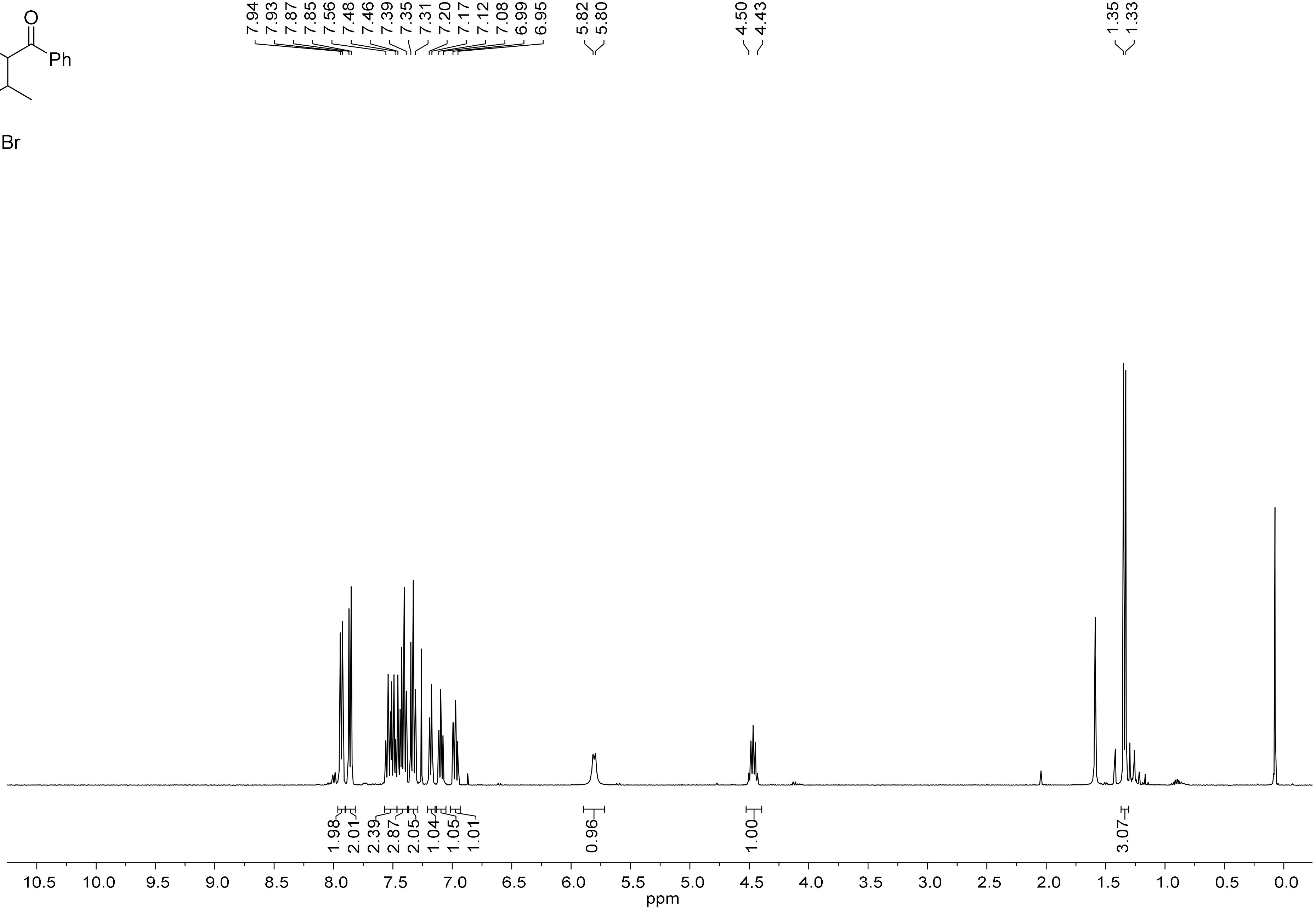

16 ^{1}H NMR (300 MHz, $CDCl_3$)

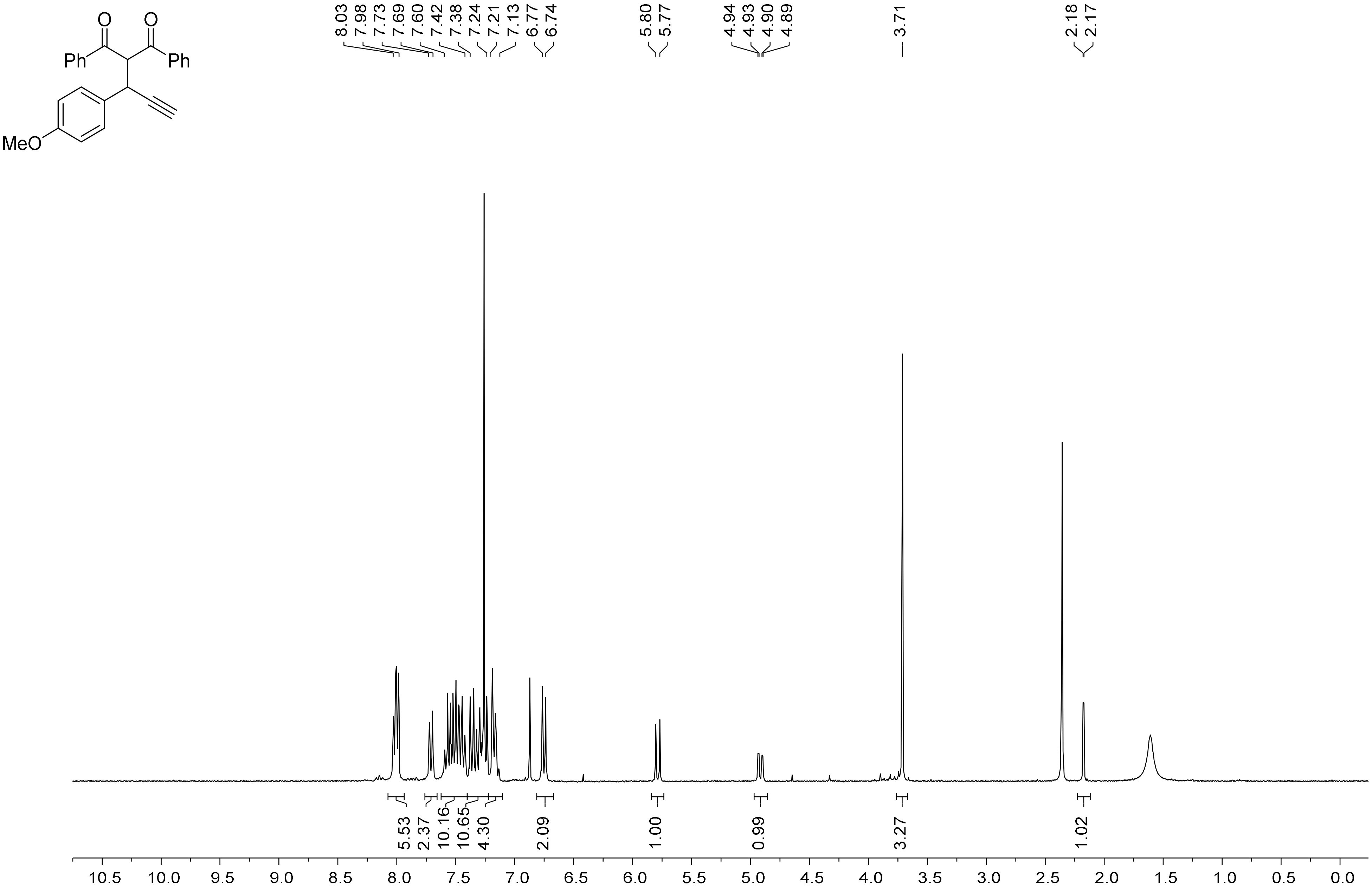

16 $^{13}C\{^{1}H\}$ NMR (126 MHz, $CDCl_3$)

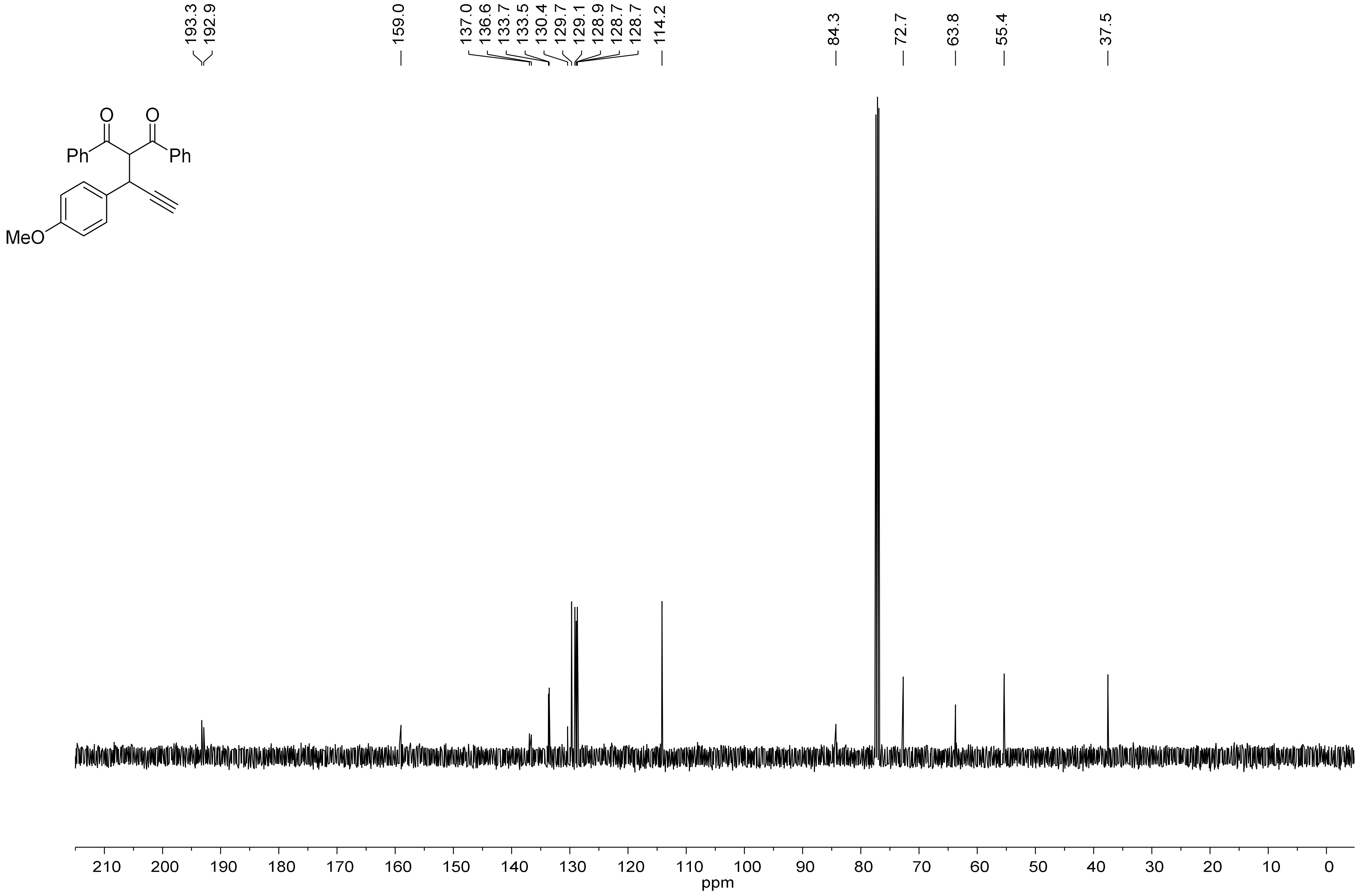

S17 ^{1}H NMR (300 MHz, $CDCl_3$)

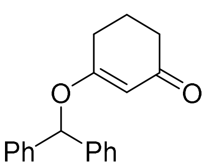

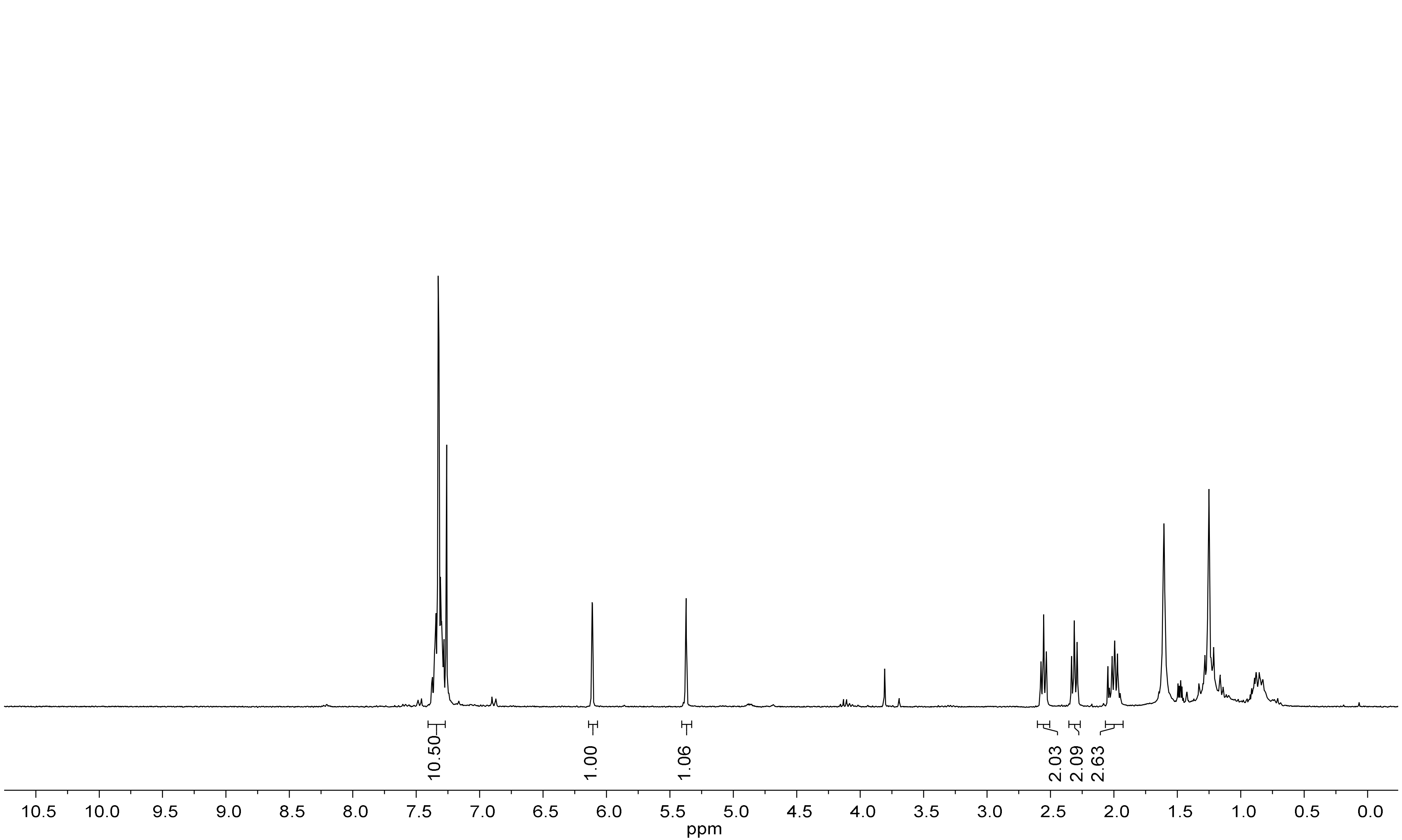

S17 $^{13}C\{^{1}H\}$ NMR (126 MHz, $CDCl_3$)

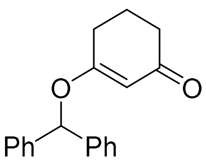

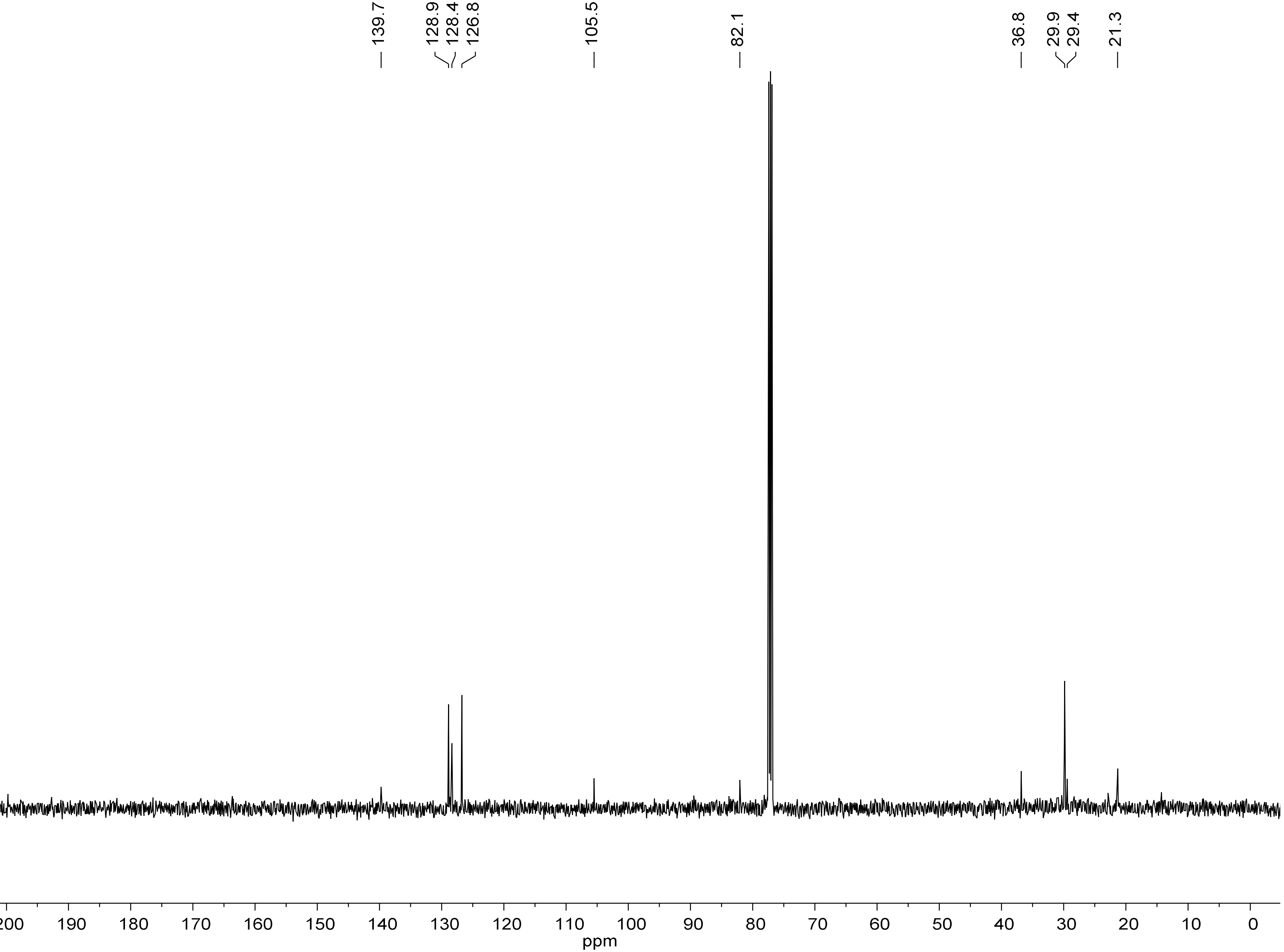

18 ^{1}H NMR (400 MHz, $CDCl_3$)

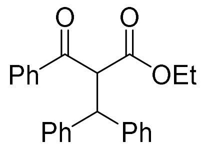

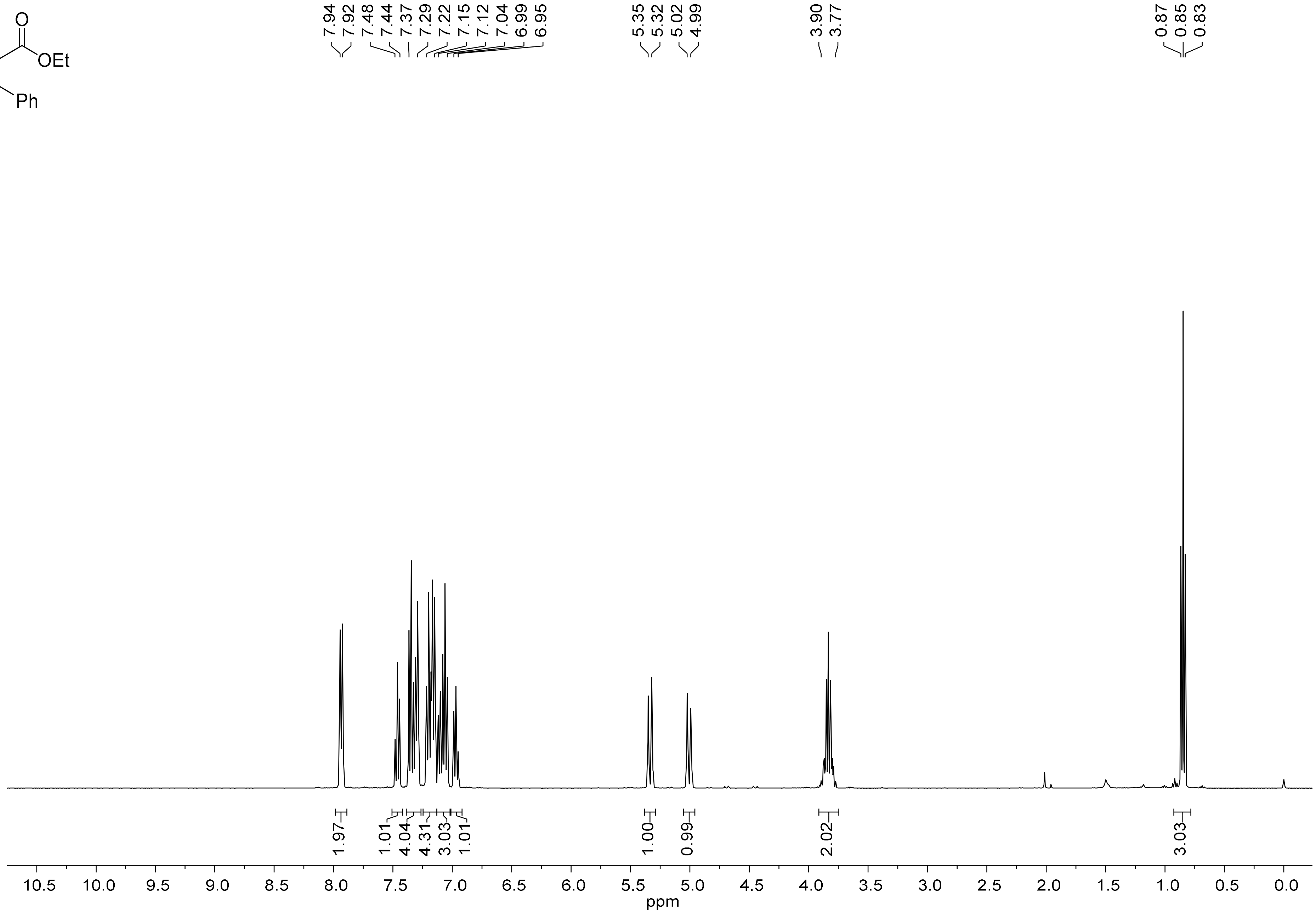

19 (57:43 dr) ^{1}H NMR (300 MHz, $CDCl_3$)

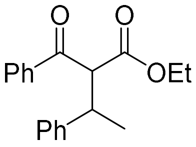

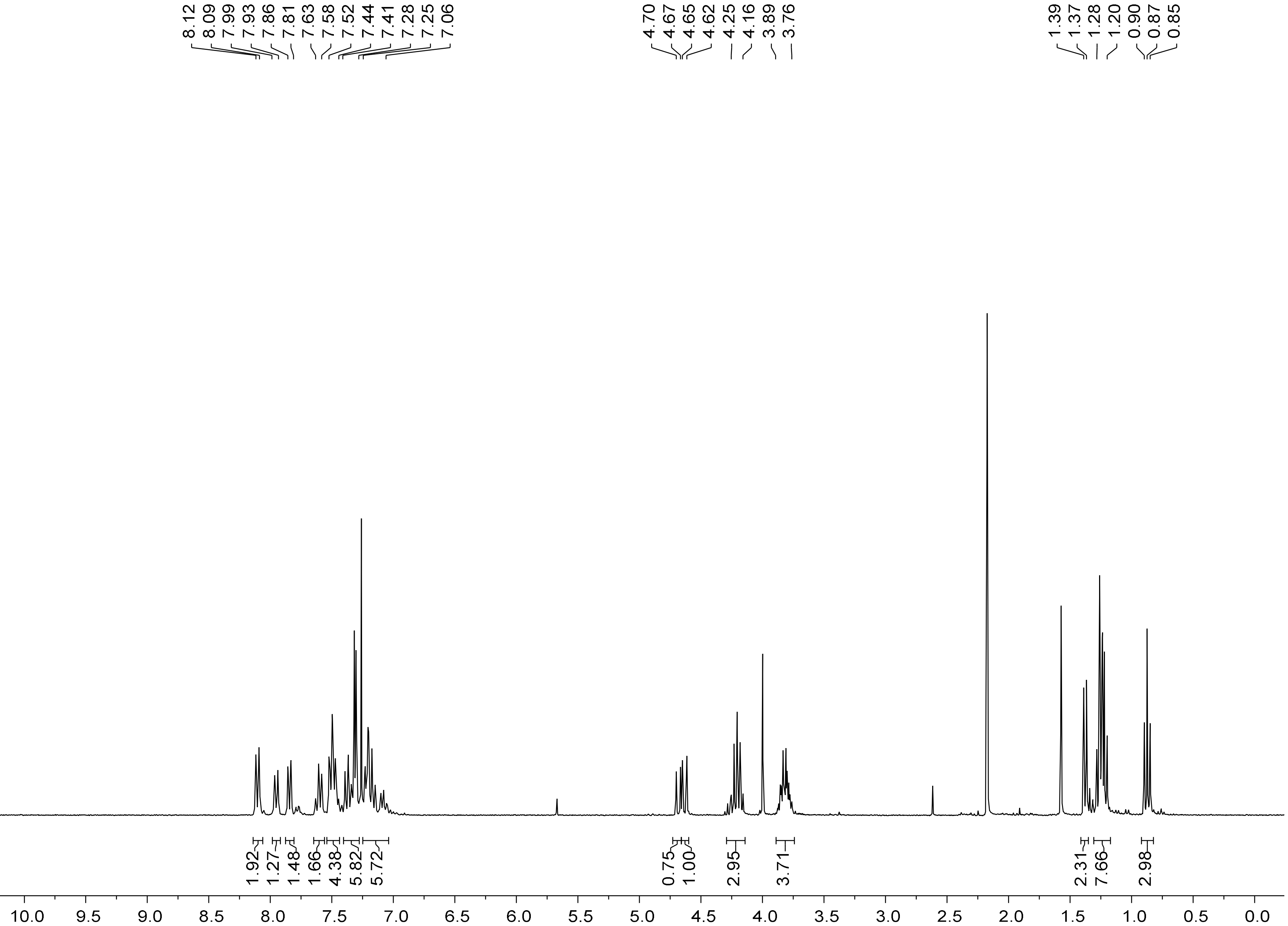

20 (54:46 dr) ^{1}H NMR (300 MHz, $CDCl_3$)

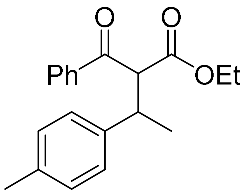

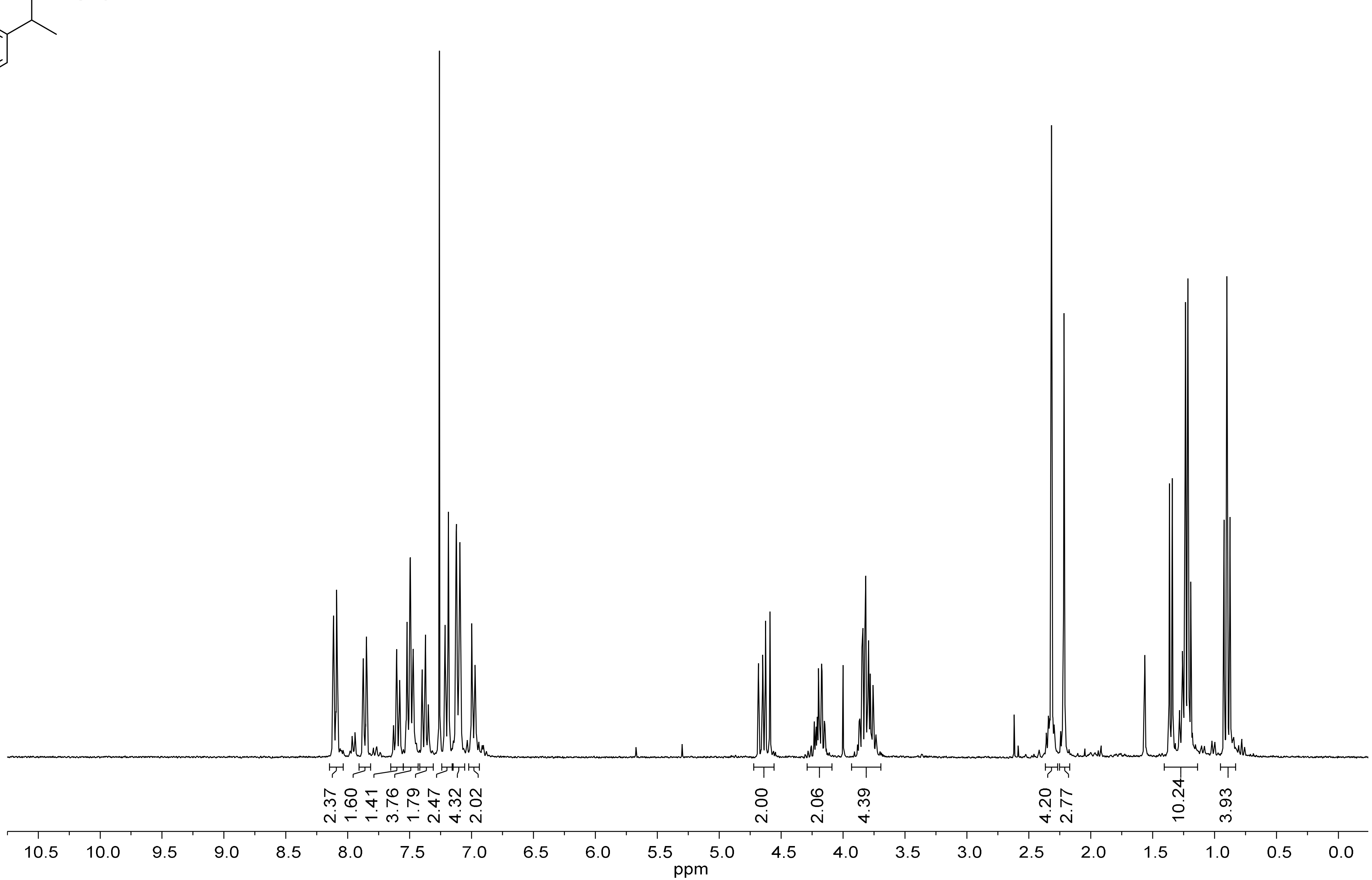

20 (54:46 dr) $^{13}C\{^{1}H\}$ NMR (126 MHz, $CDCl_3$)

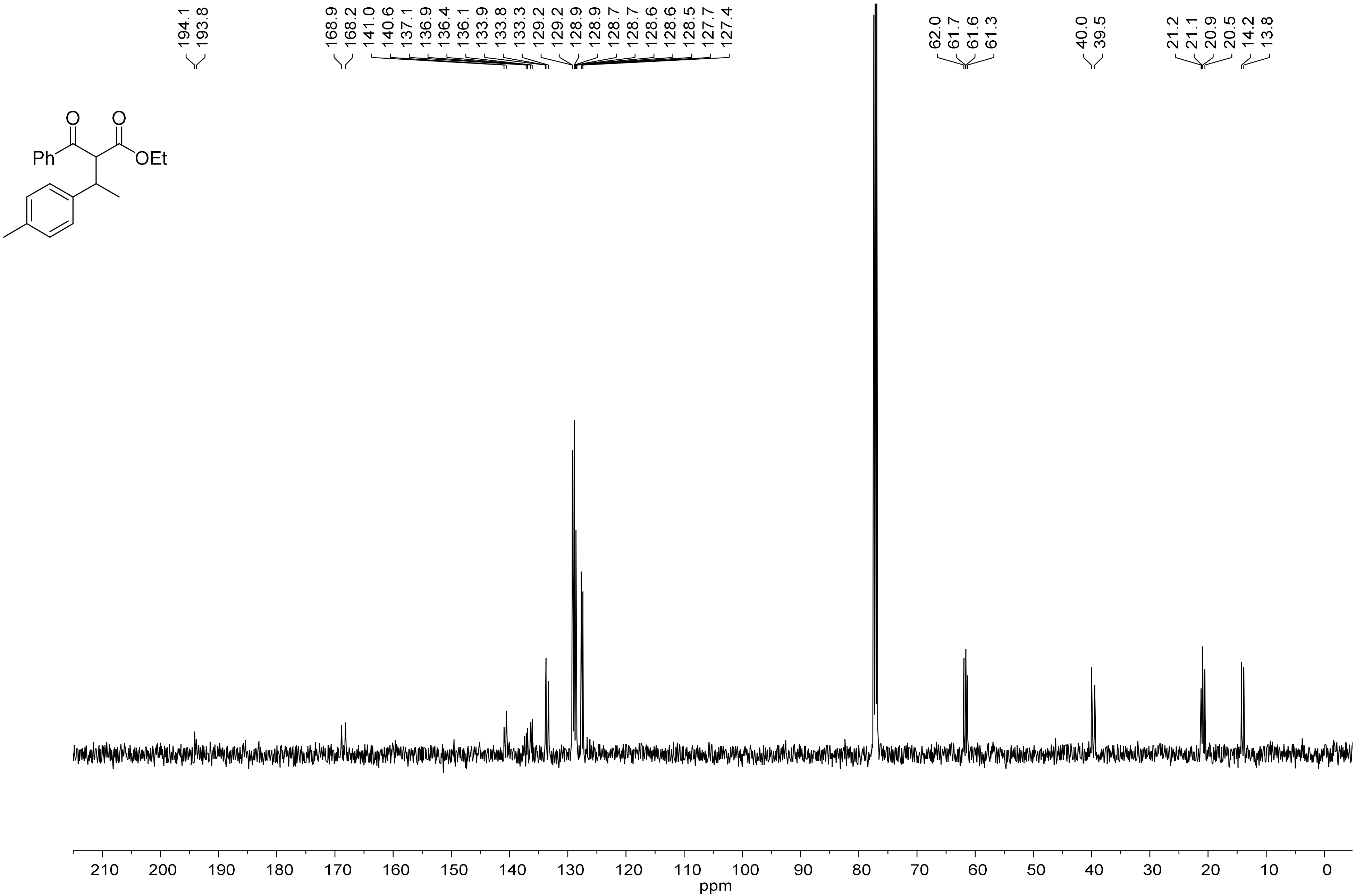

21 (63:37 dr) ^{1}H NMR (400 MHz, $CDCl_3$)

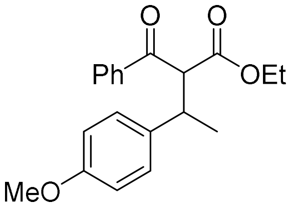

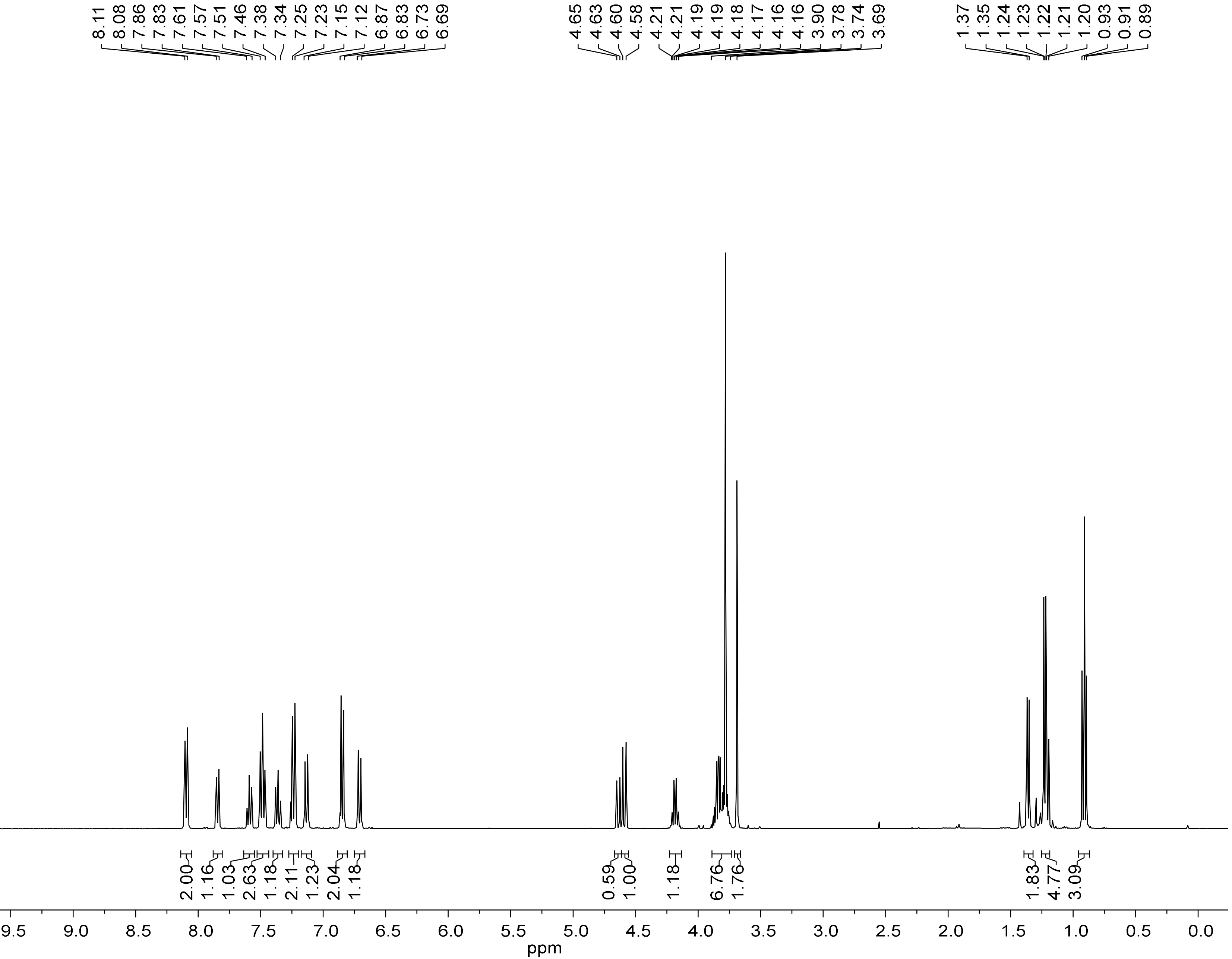

22 (52:48 dr) ^{1}H NMR (300 MHz, $CDCl_3$)

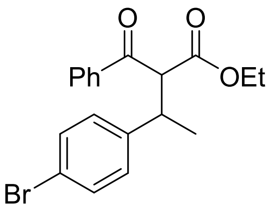

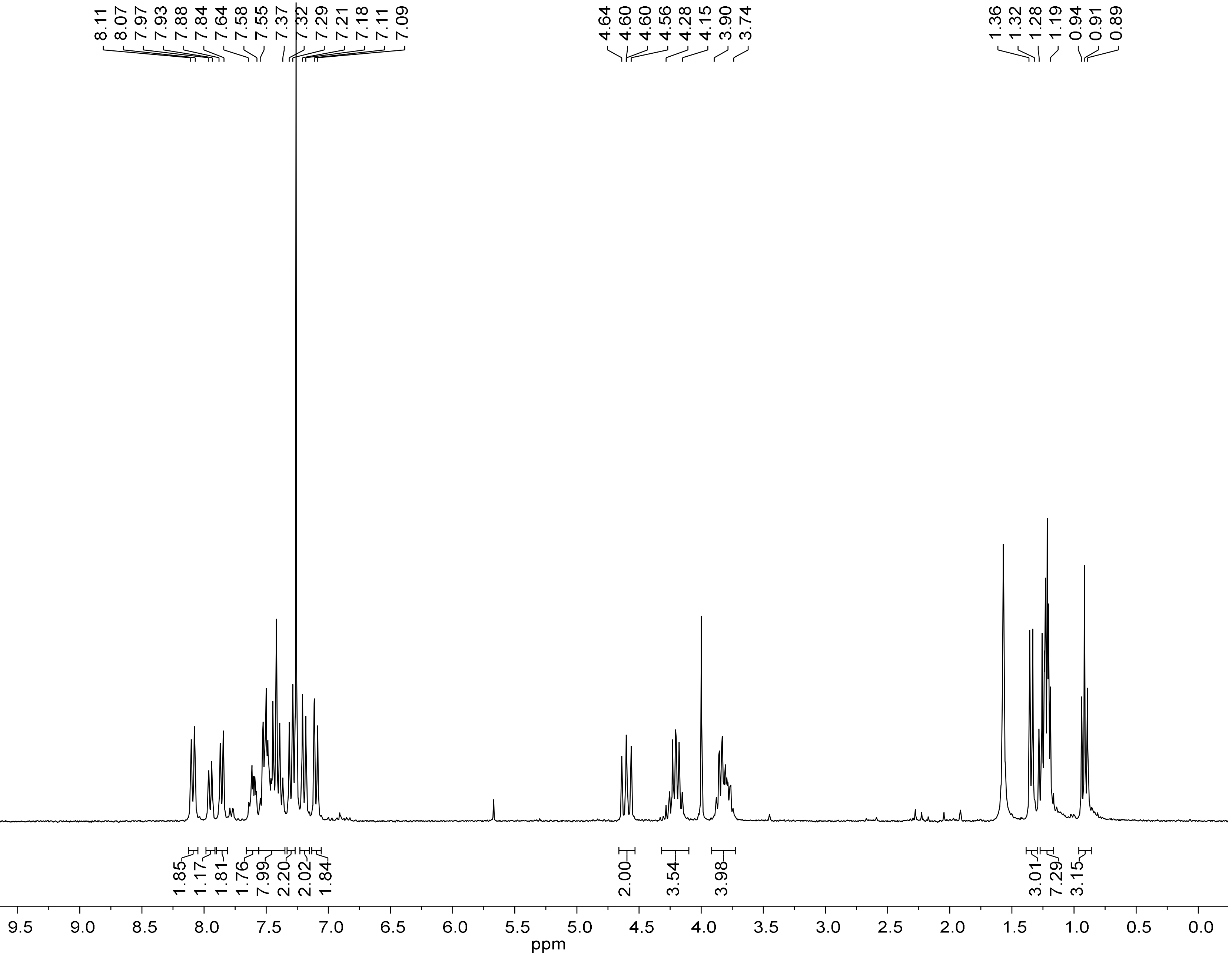

22 (52:48 dr) $^{13}C\{^{1}H\}$ NMR (126 MHz, $CDCl_3$)

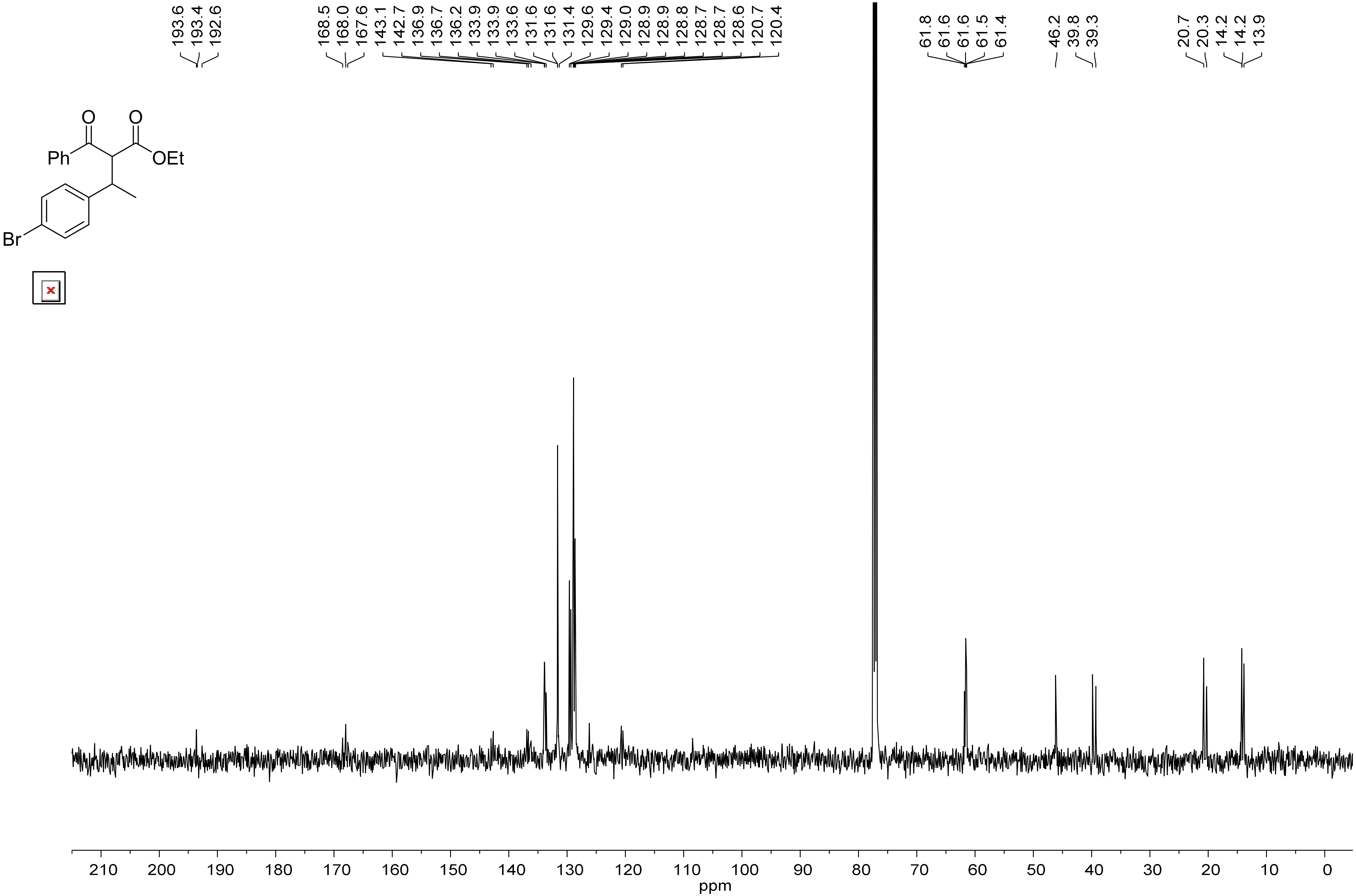

23 ^{1}H NMR (300 MHz, $CDCl_3$)

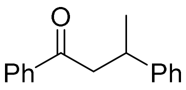

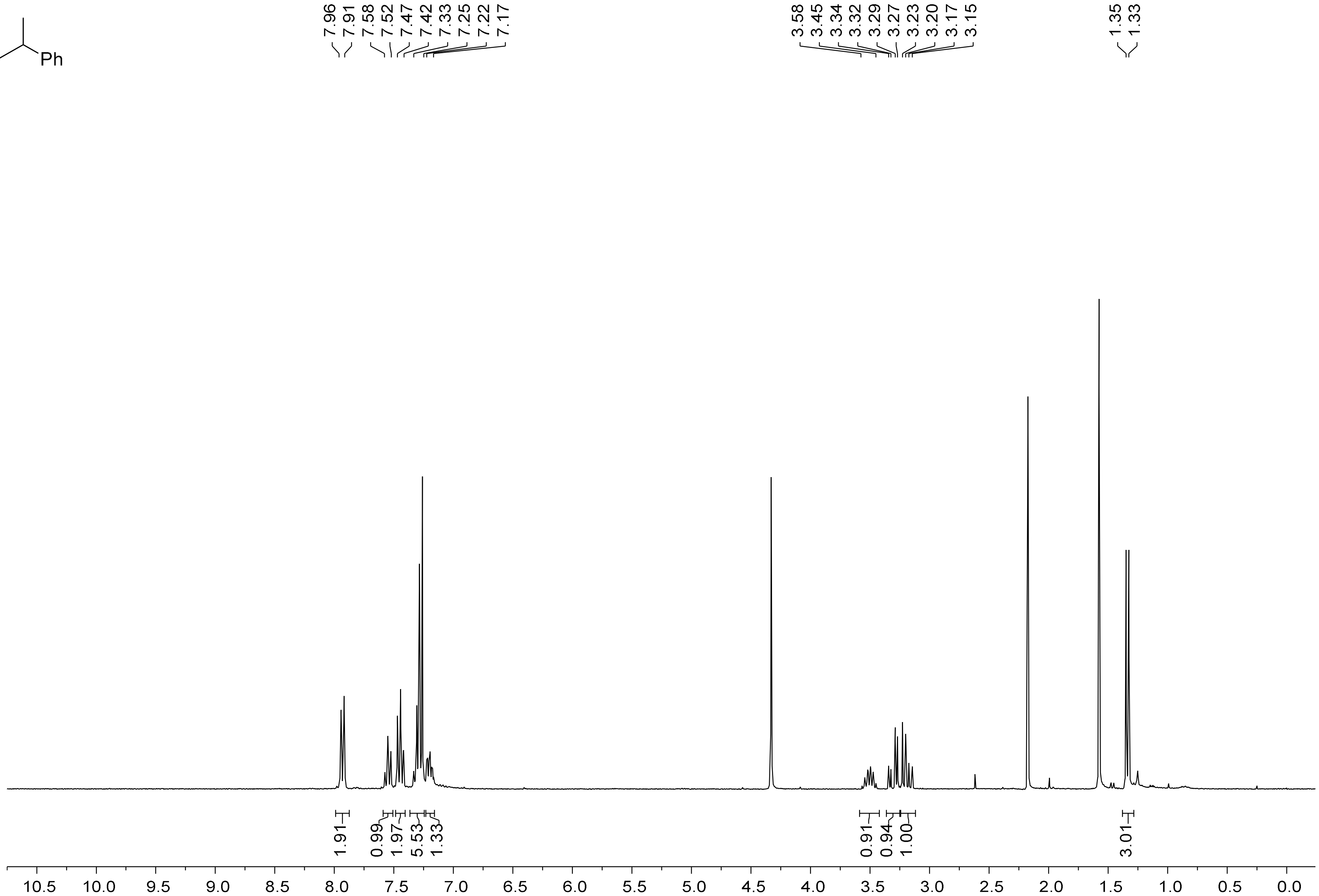

24 ^{1}H NMR (300 MHz, $CDCl_3$)

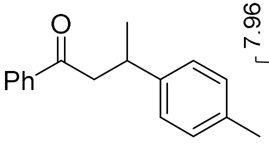

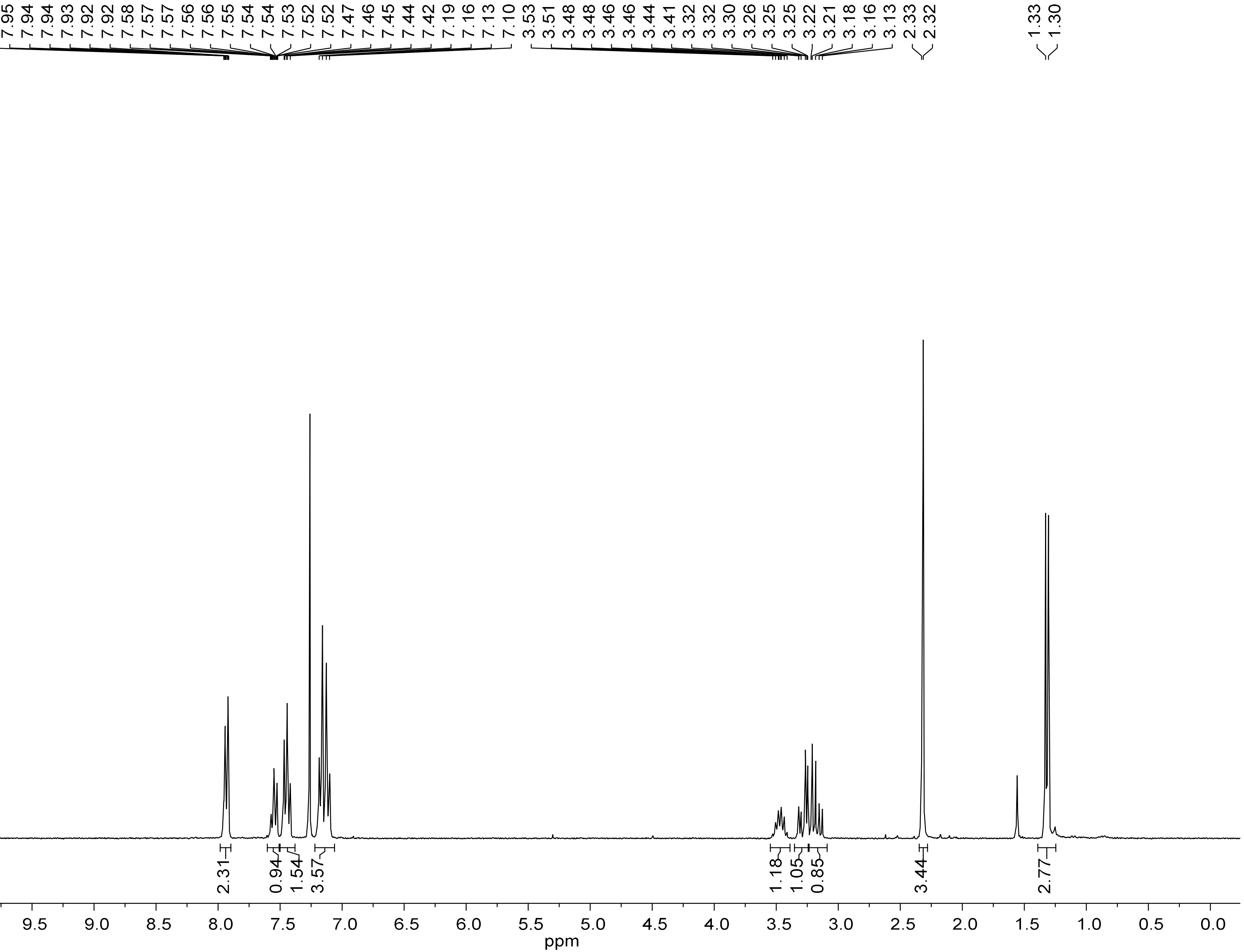

25 ^{1}H NMR (300 MHz, $CDCl_3$)

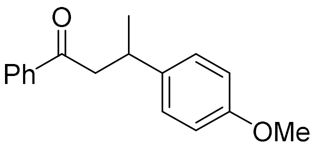

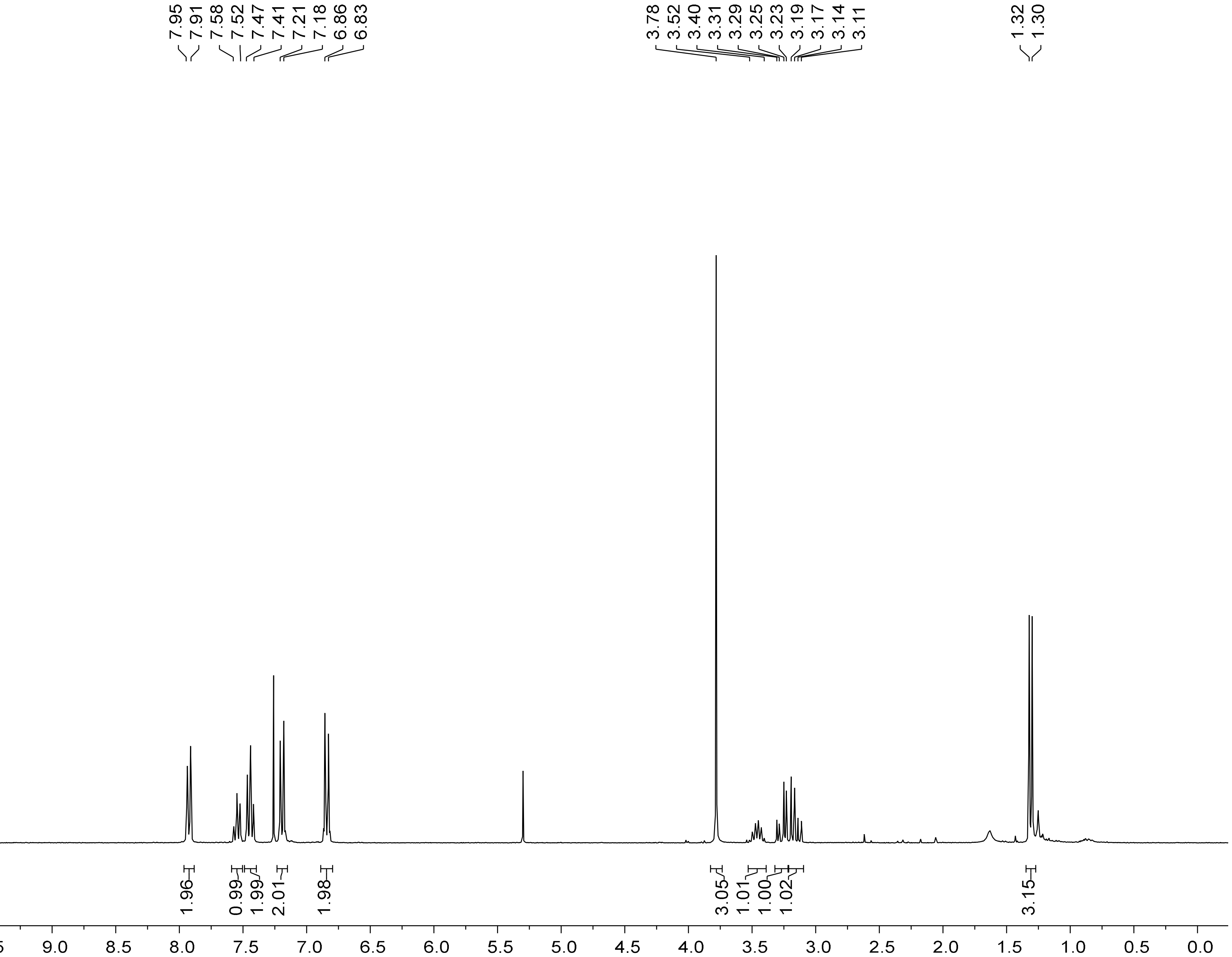

27 ^{1}H NMR (300 MHz, $CDCl_3$)

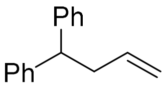

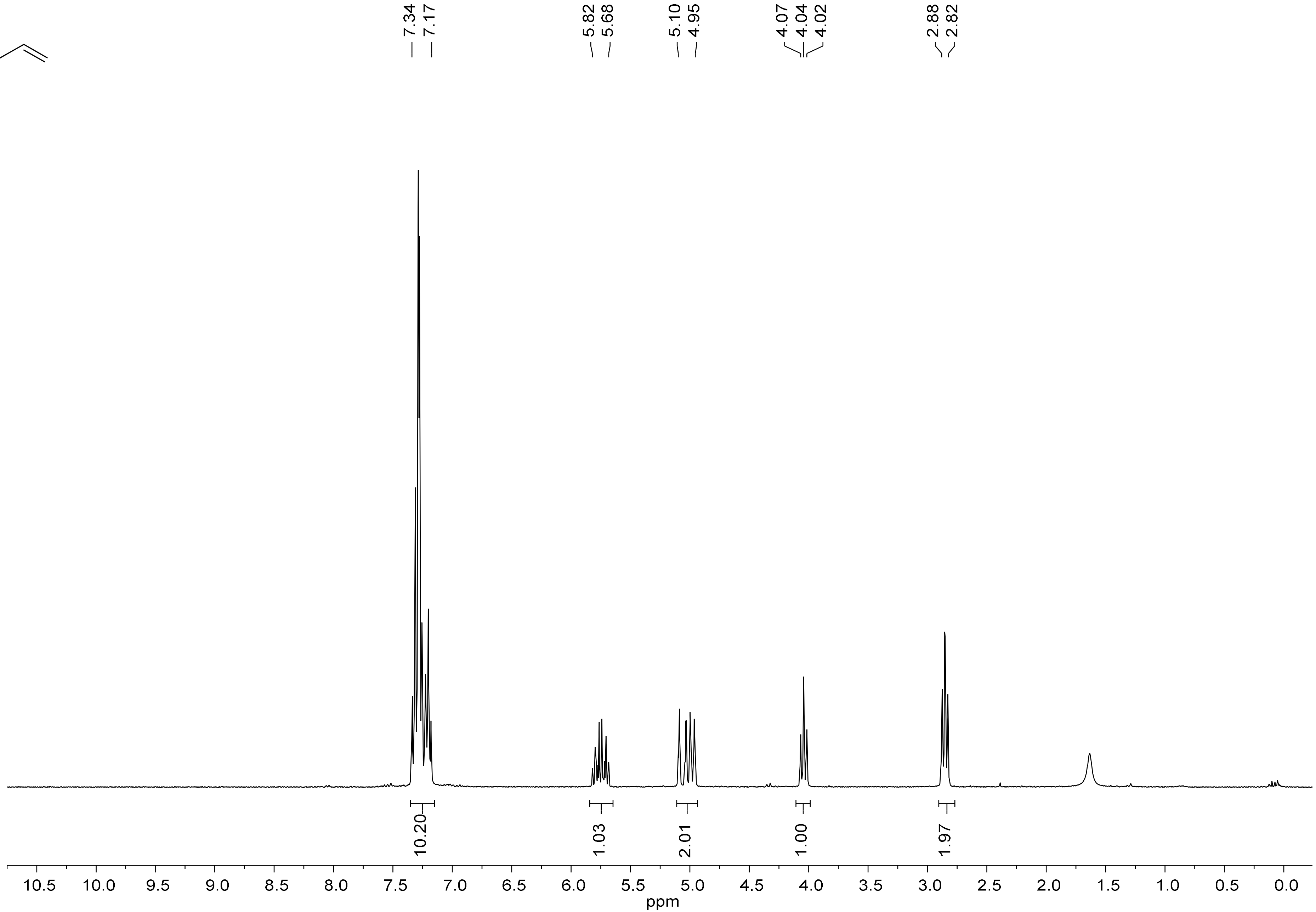

28 ^{1}H NMR (300 MHz, $CDCl_3$)

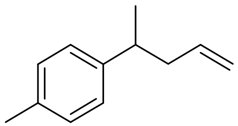

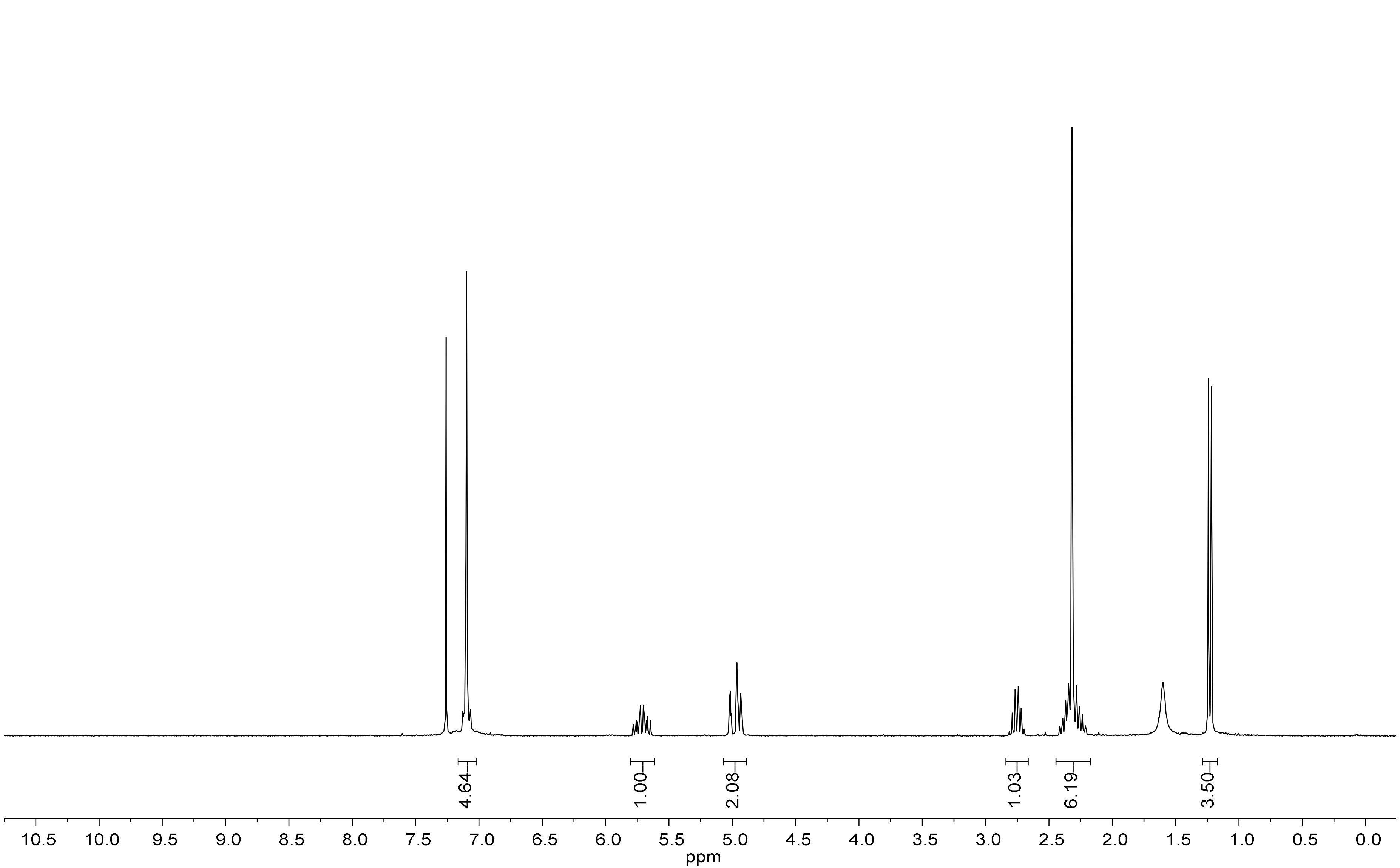

29 ^{1}H NMR (300 MHz, $CDCl_3$)

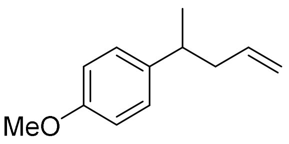

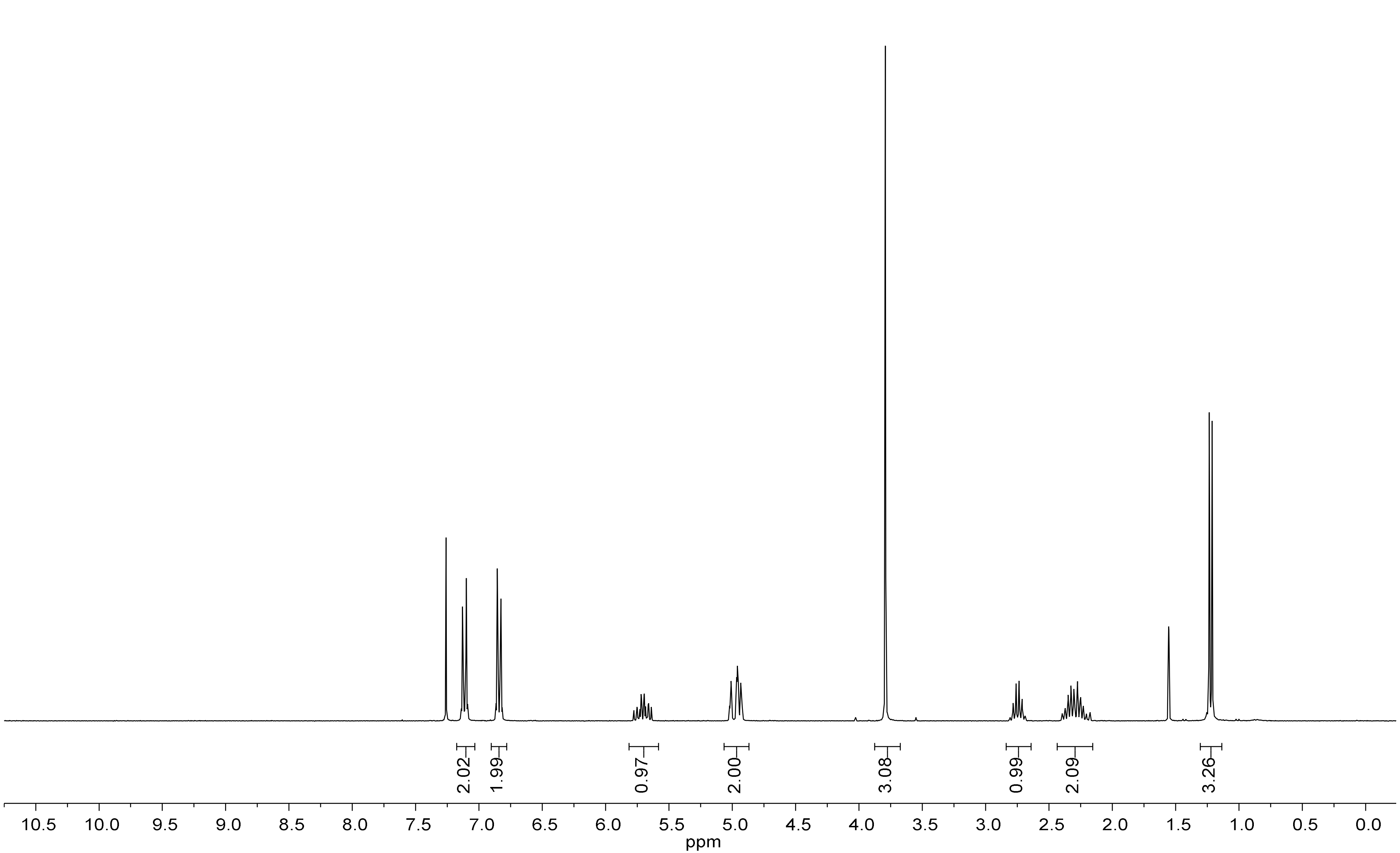

30 ^{1}H NMR (300 MHz, $CDCl_3$)

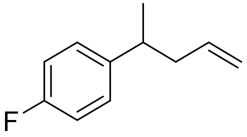

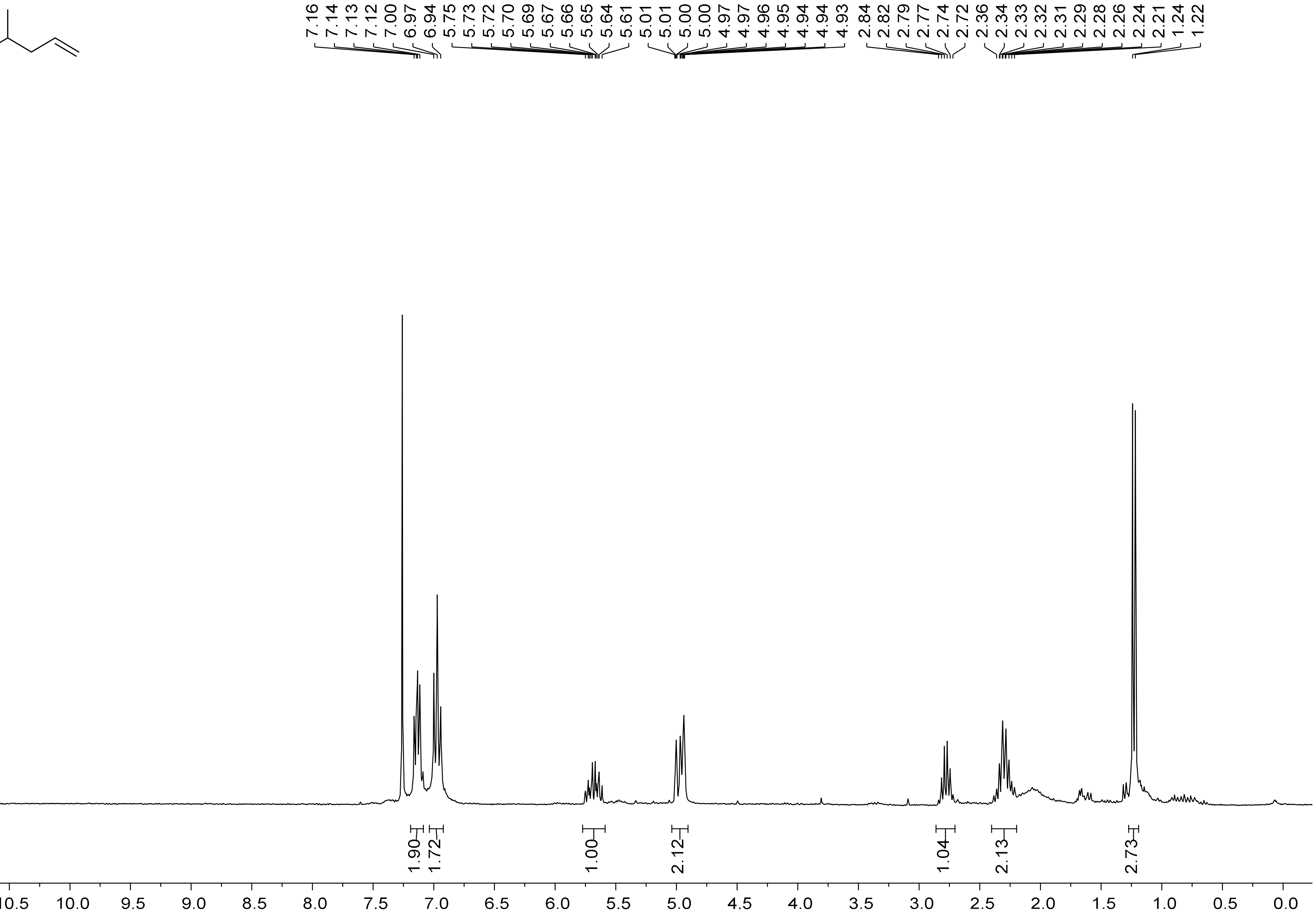

31 ^{1}H NMR (300 MHz, $CDCl_3$)

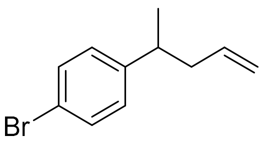

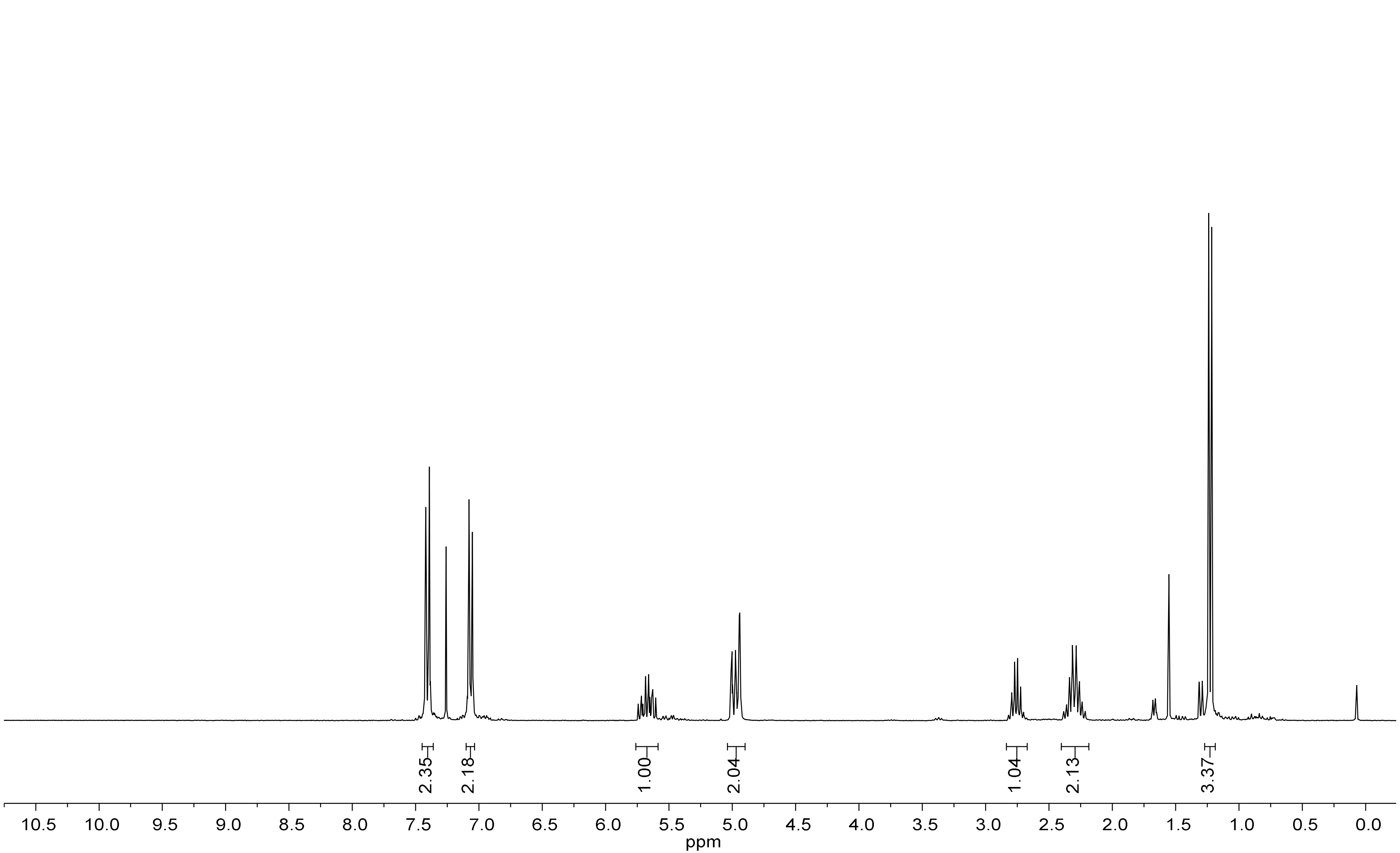

32 ^{1}H NMR (300 MHz, $CDCl_3$)

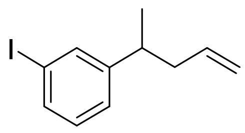

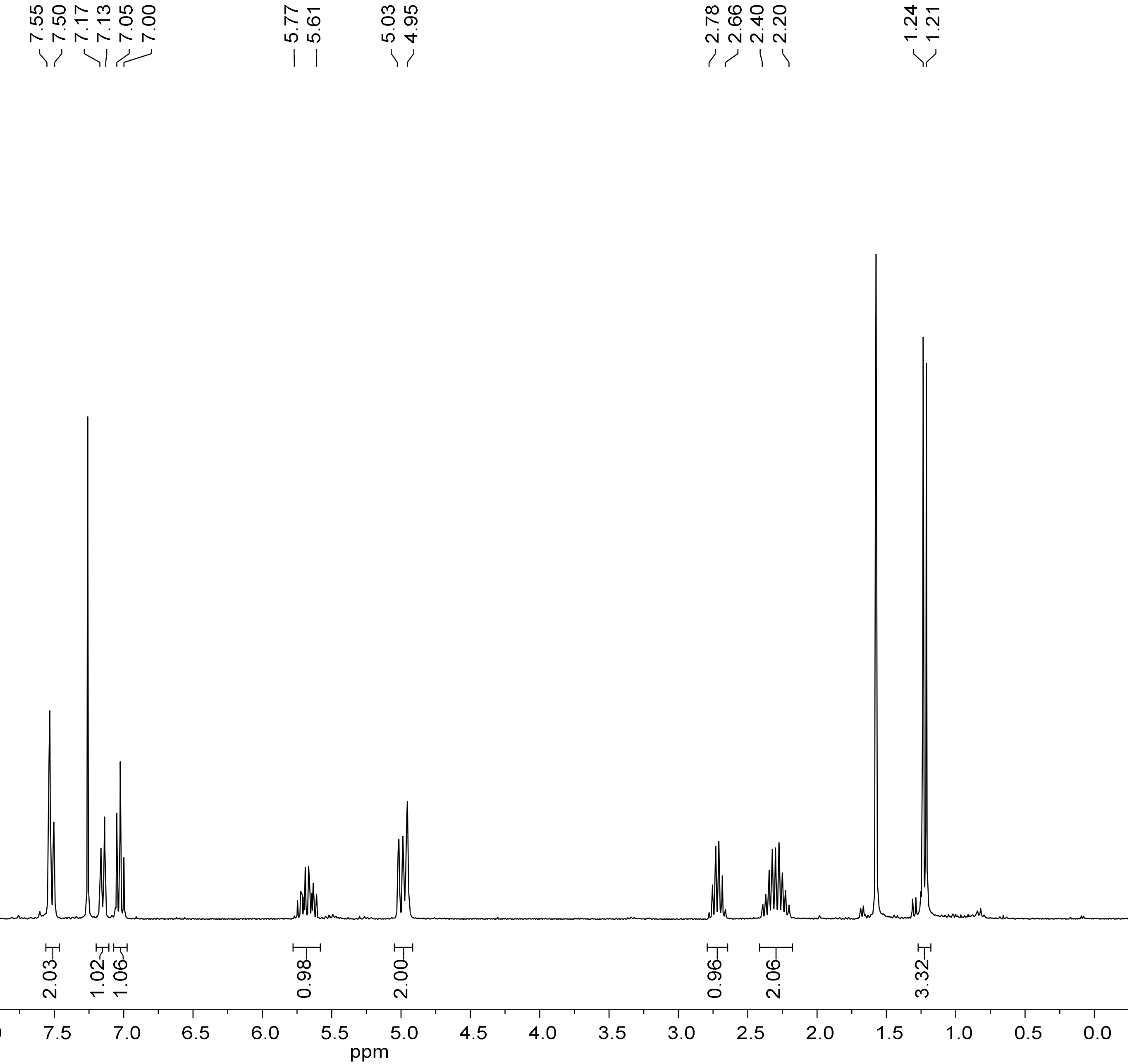

32 $^{13}C\{^{1}H\}$ NMR (126 MHz, $CDCl_3$)

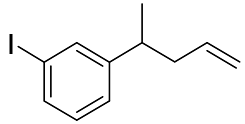

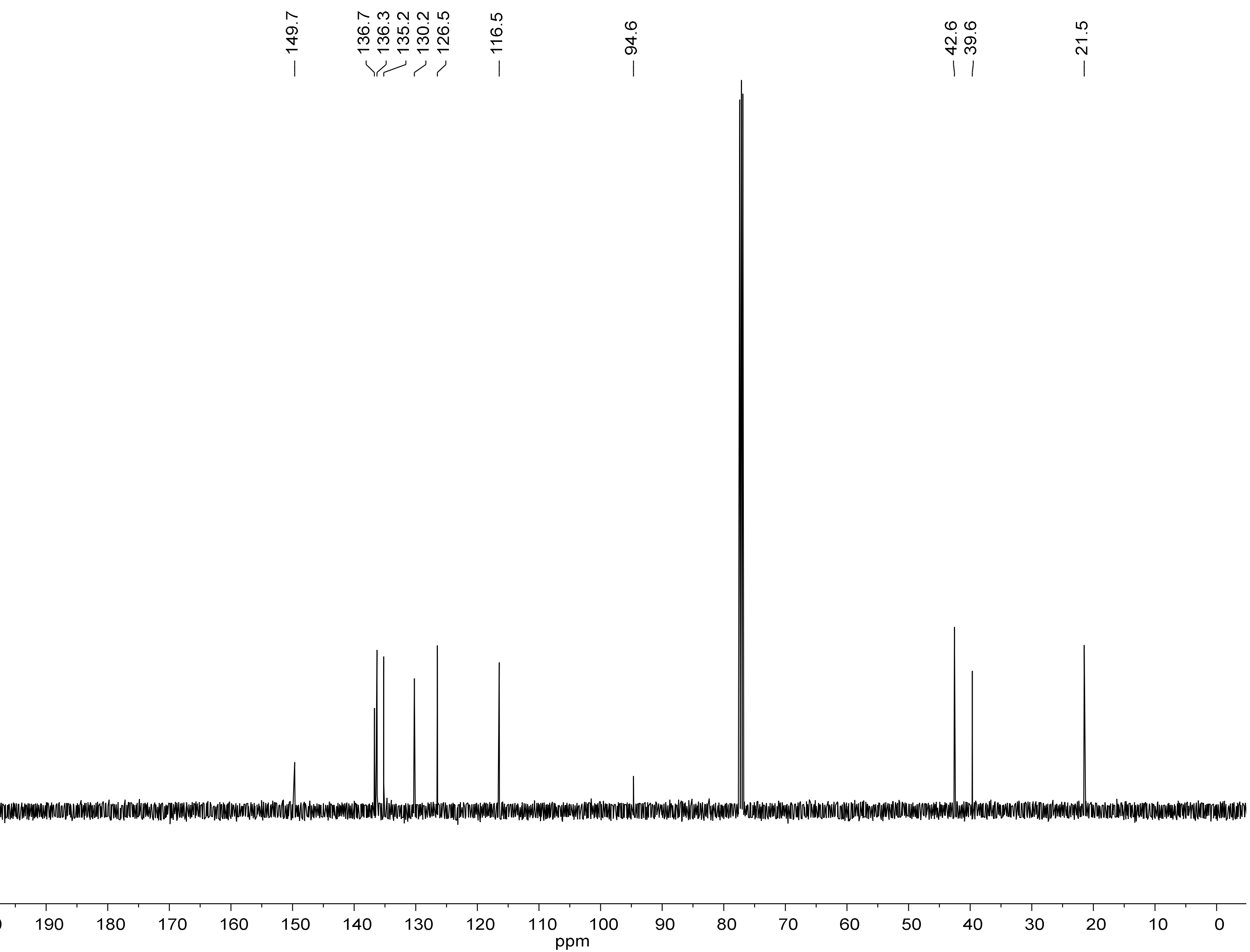

33 ^{1}H NMR (300 MHz, $CDCl_3$)

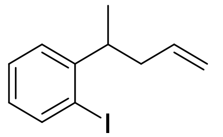

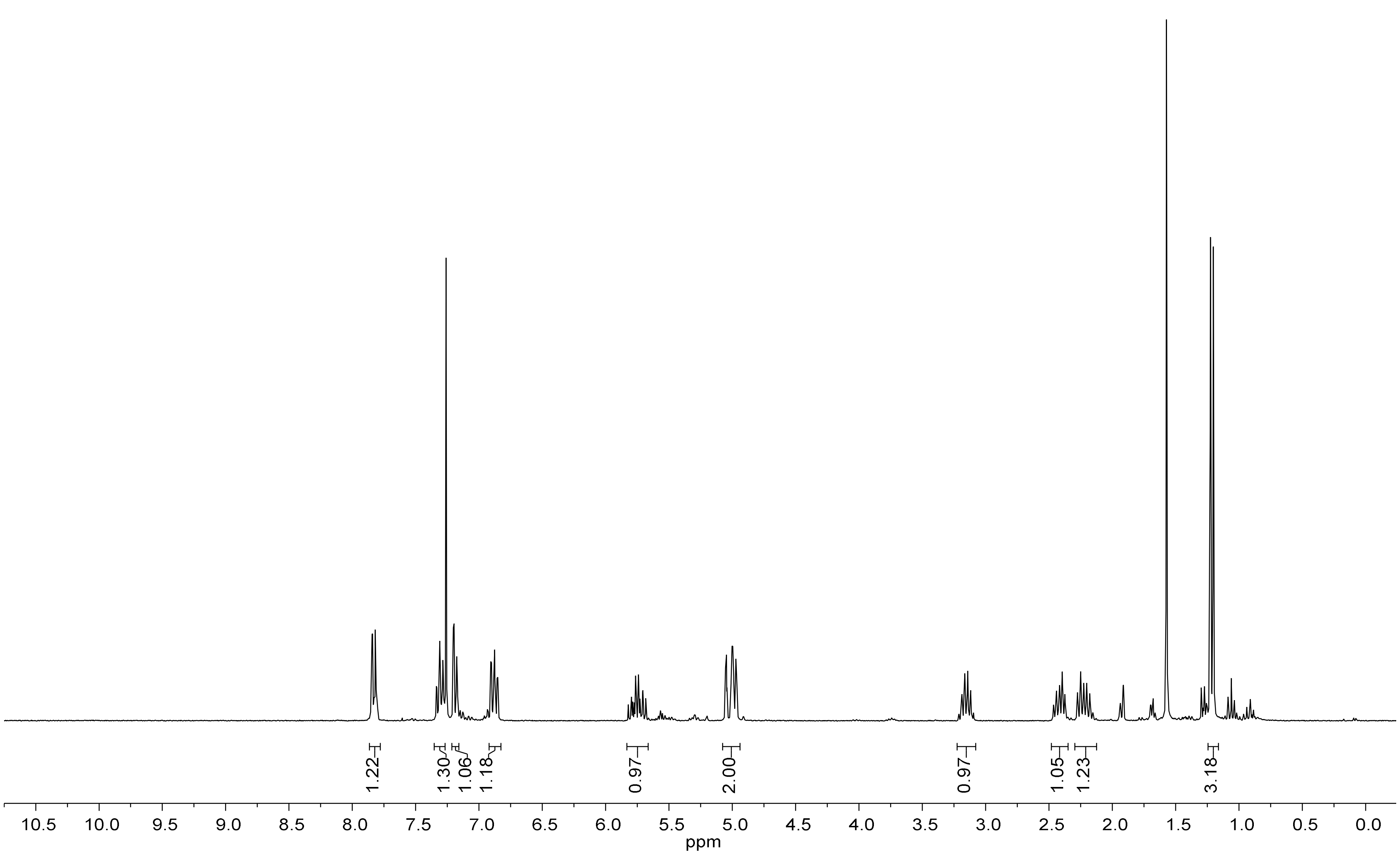

33 $^{13}C\{^{1}H\}$ NMR (126 MHz, $CDCl_3$)

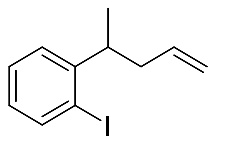

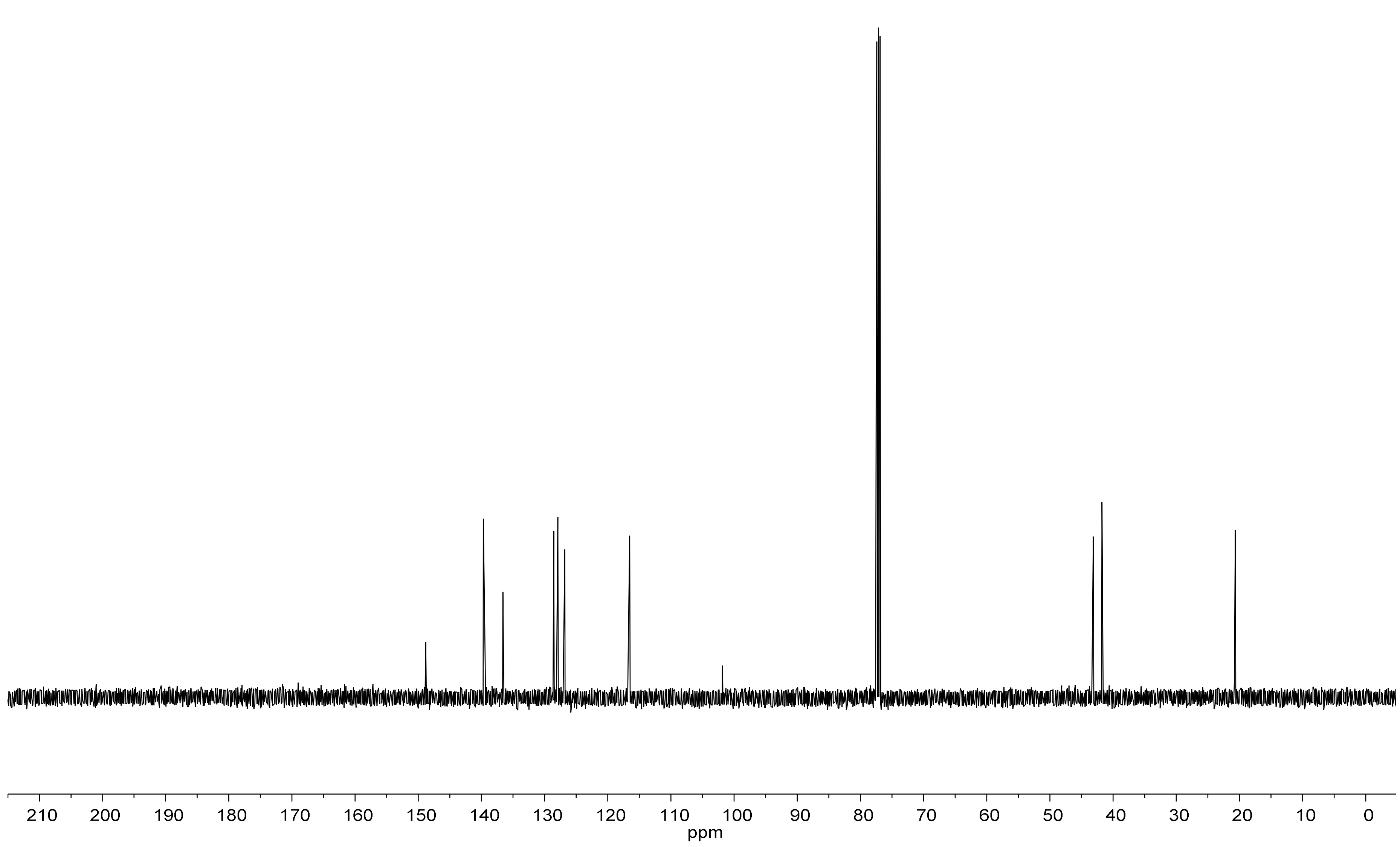

34 ^{1}H NMR (300 MHz, $CDCl_3$)

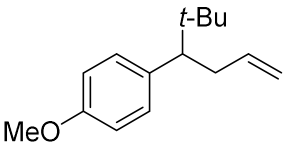

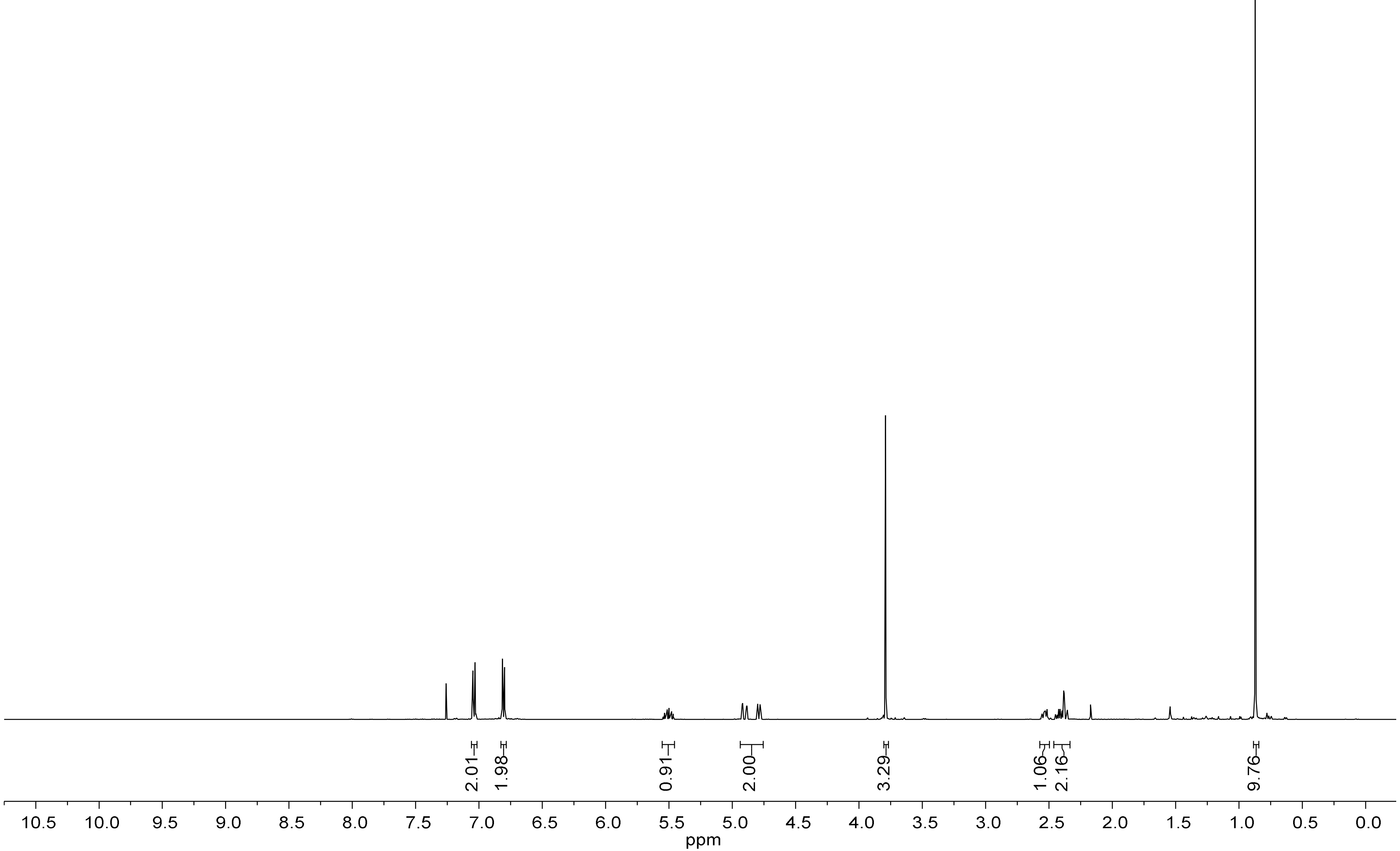

34 $^{13}C\{^{1}H\}$ NMR (126 MHz, $CDCl_3$)

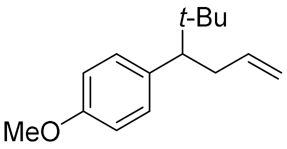

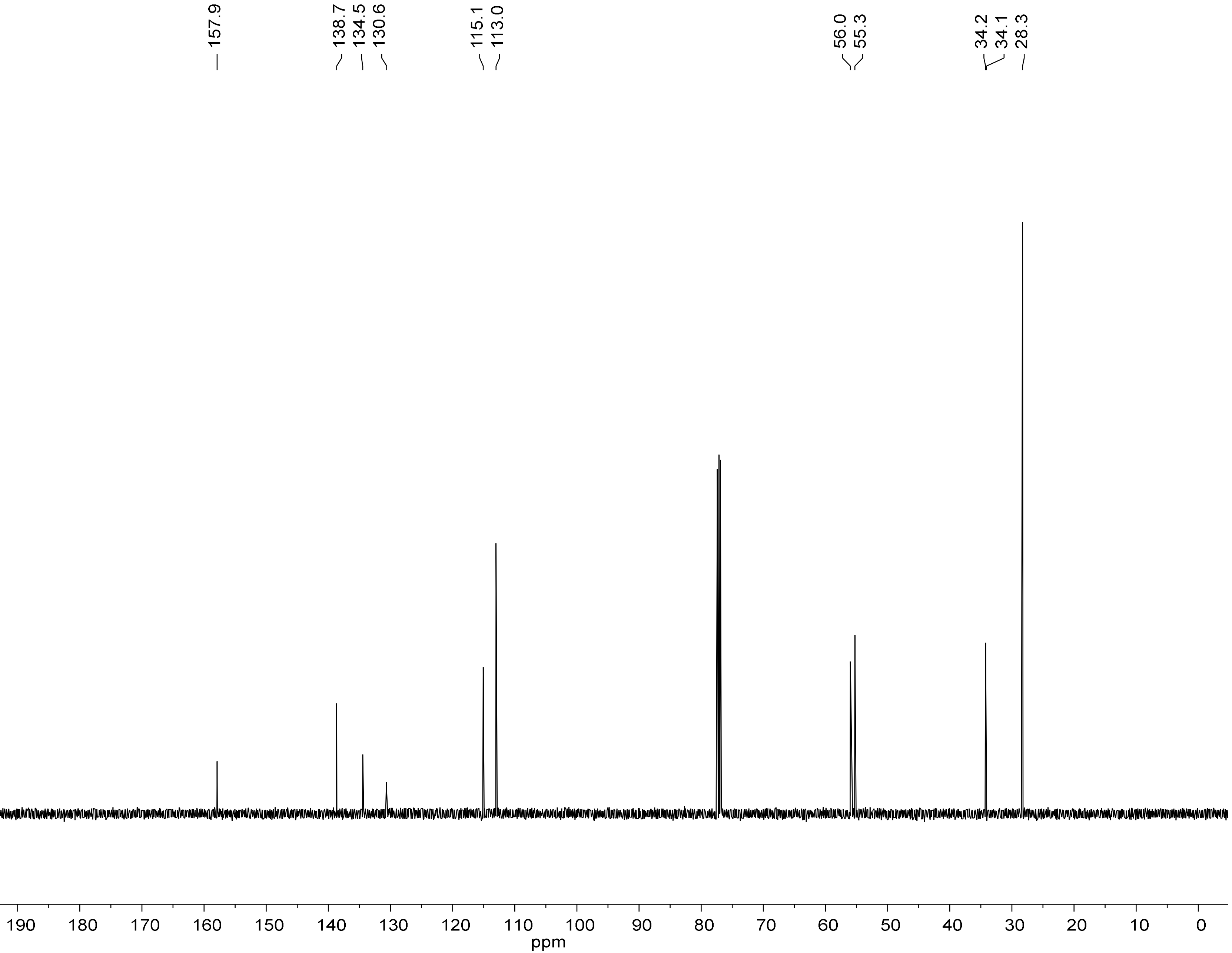

35 ^1H NMR (300 MHz, $CDCl_3$)

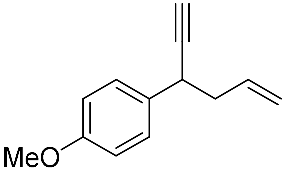

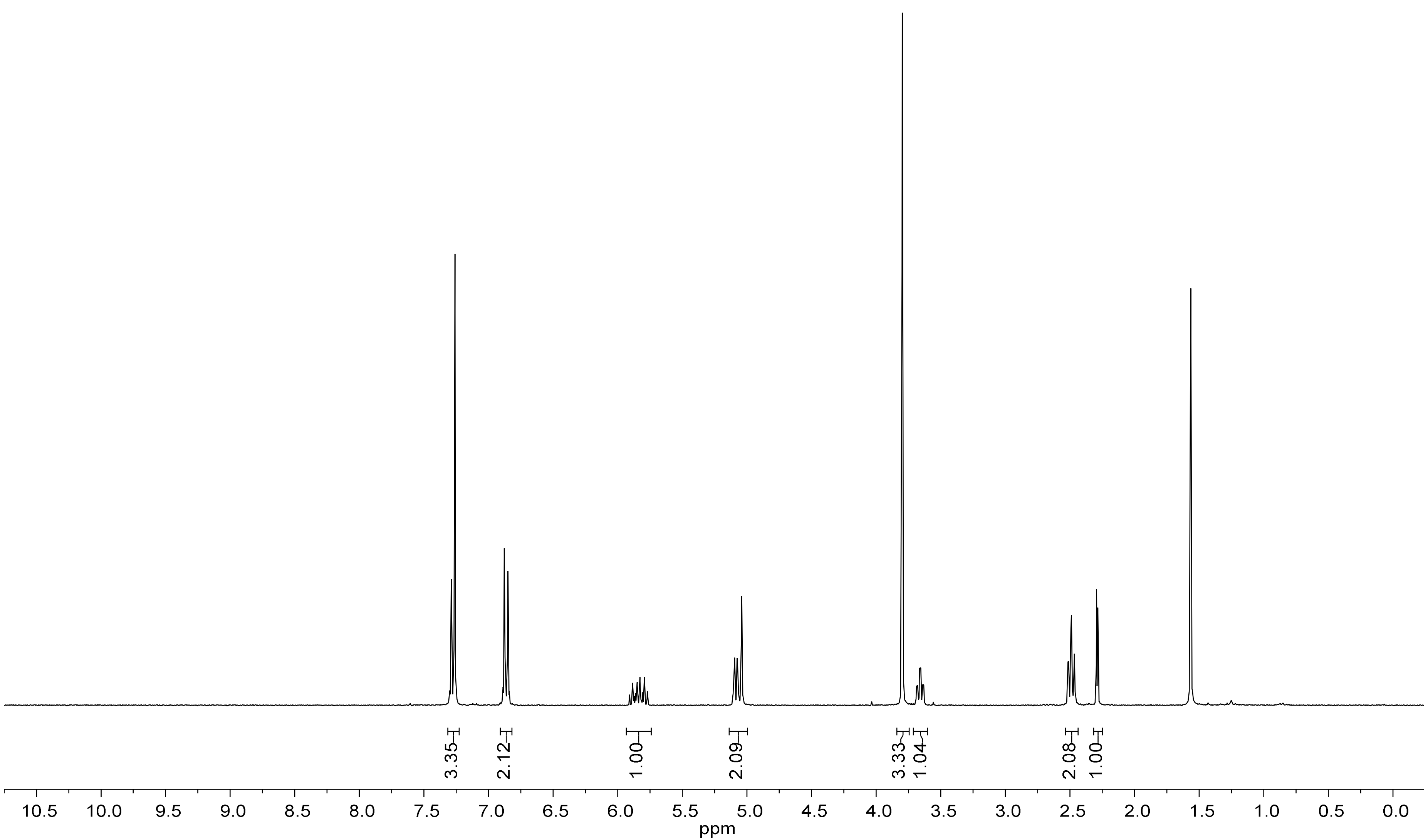

36 ^{1}H NMR (300 MHz, $CDCl_3$)

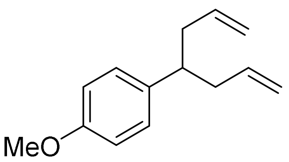

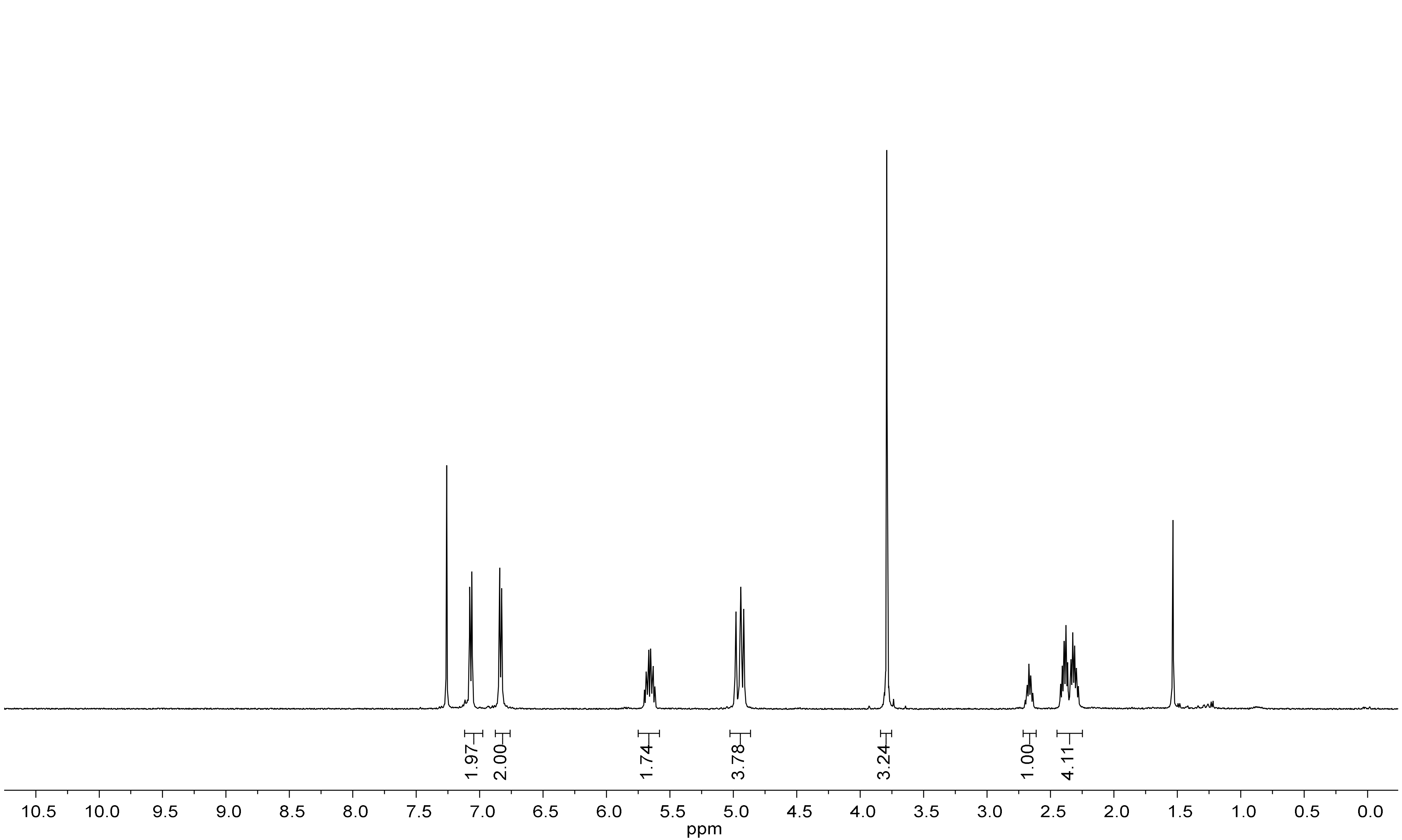

37 ^{1}H NMR (300 MHz, $CDCl_3$)

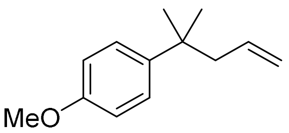

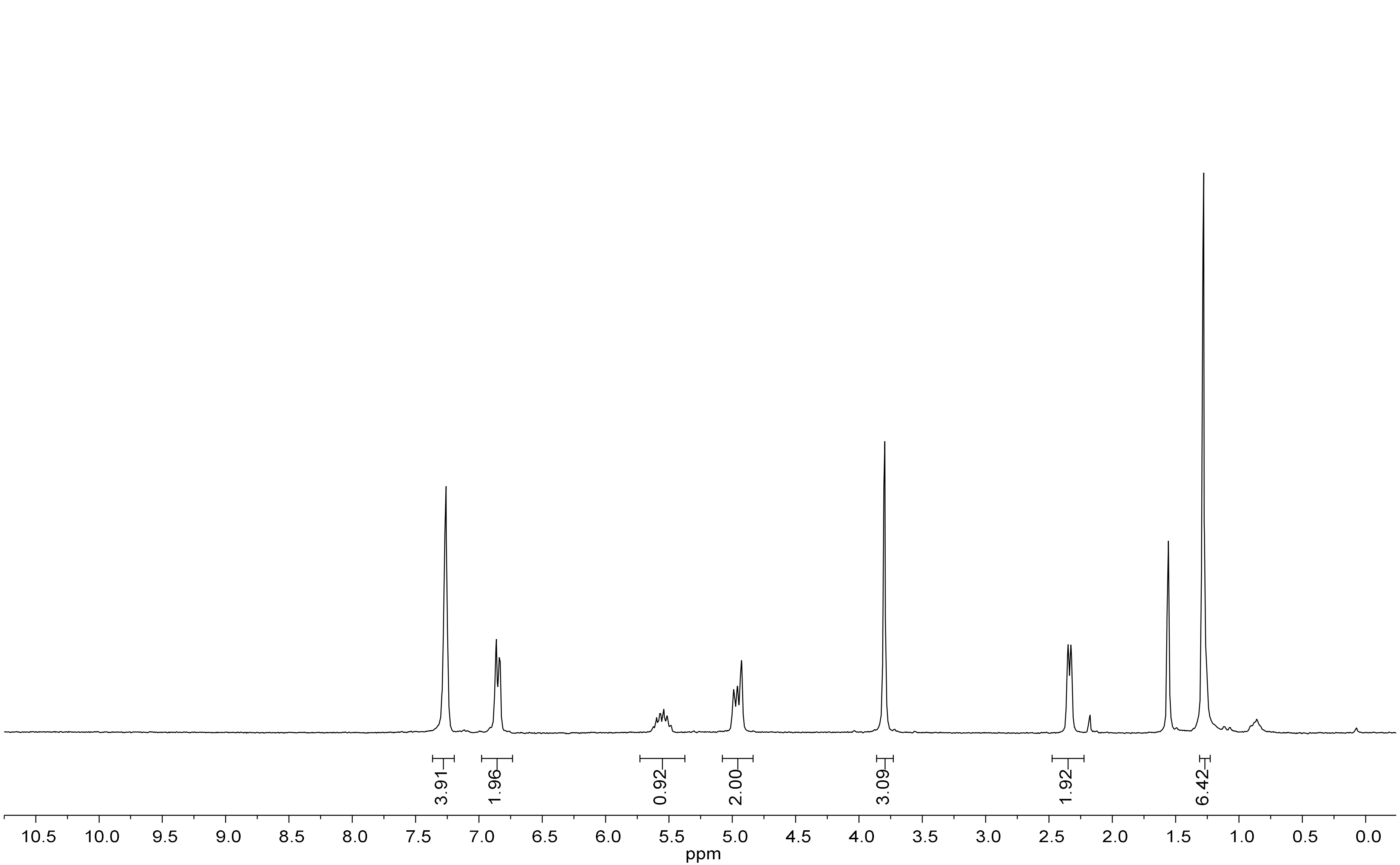

38 (64:36 dr) ^{1}H NMR (300 MHz, $CDCl_3$)

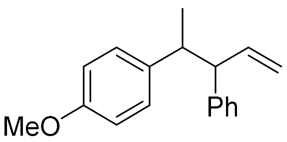

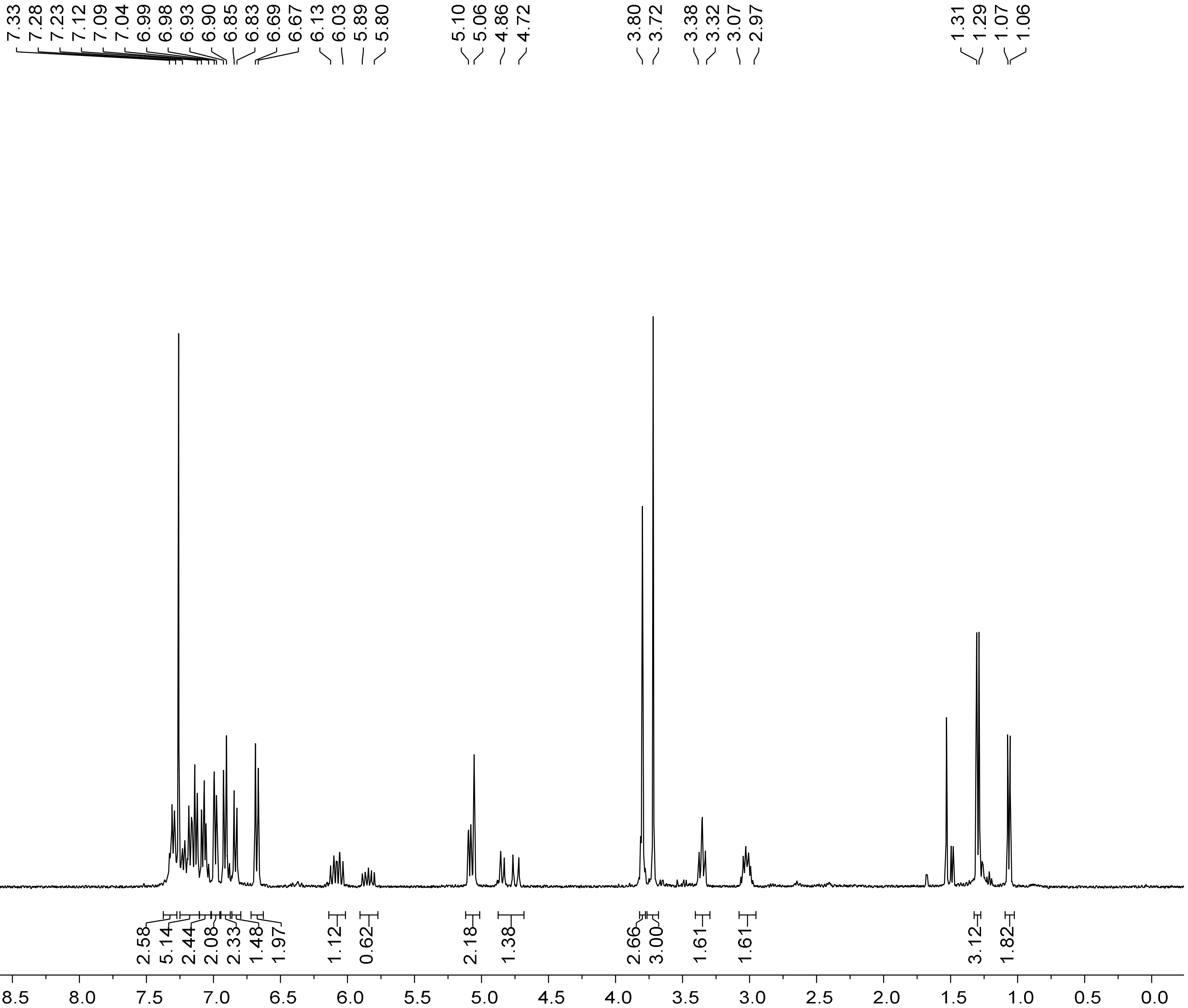

38 (64:36 dr) $^{13}C\{^{1}H\}$ NMR (126 MHz, $CDCl_3$)

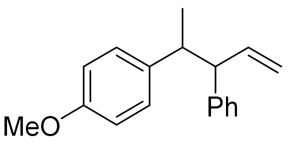

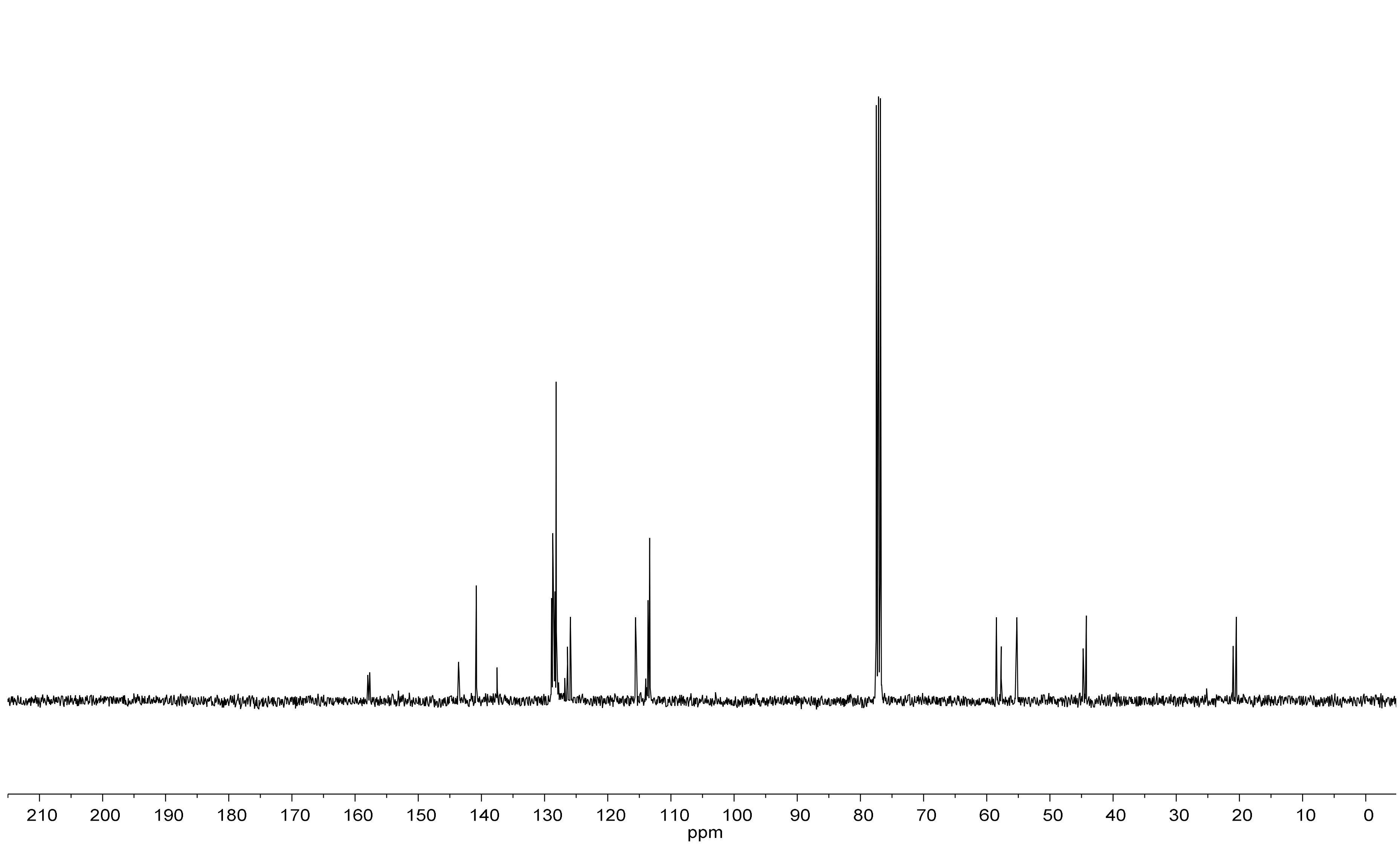

S13 and **S14** (87:13) ^{1}H NMR (400 MHz, $CDCl_3$)

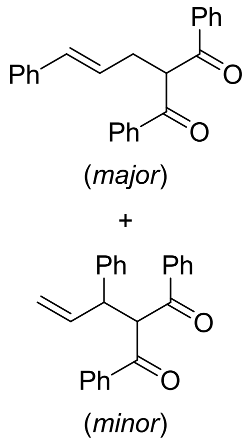

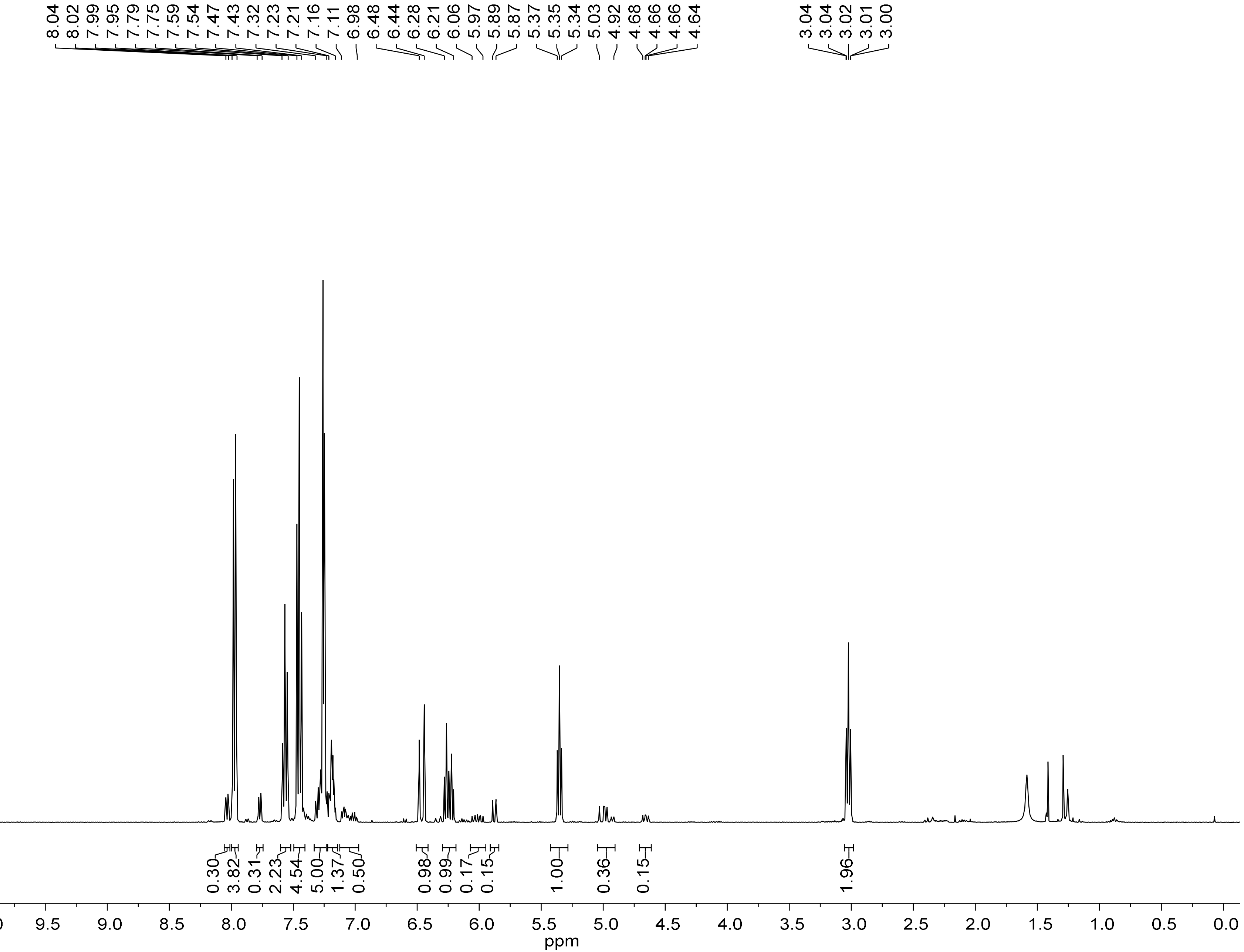

S15 ^{1}H NMR (300 MHz, $CDCl_3$)

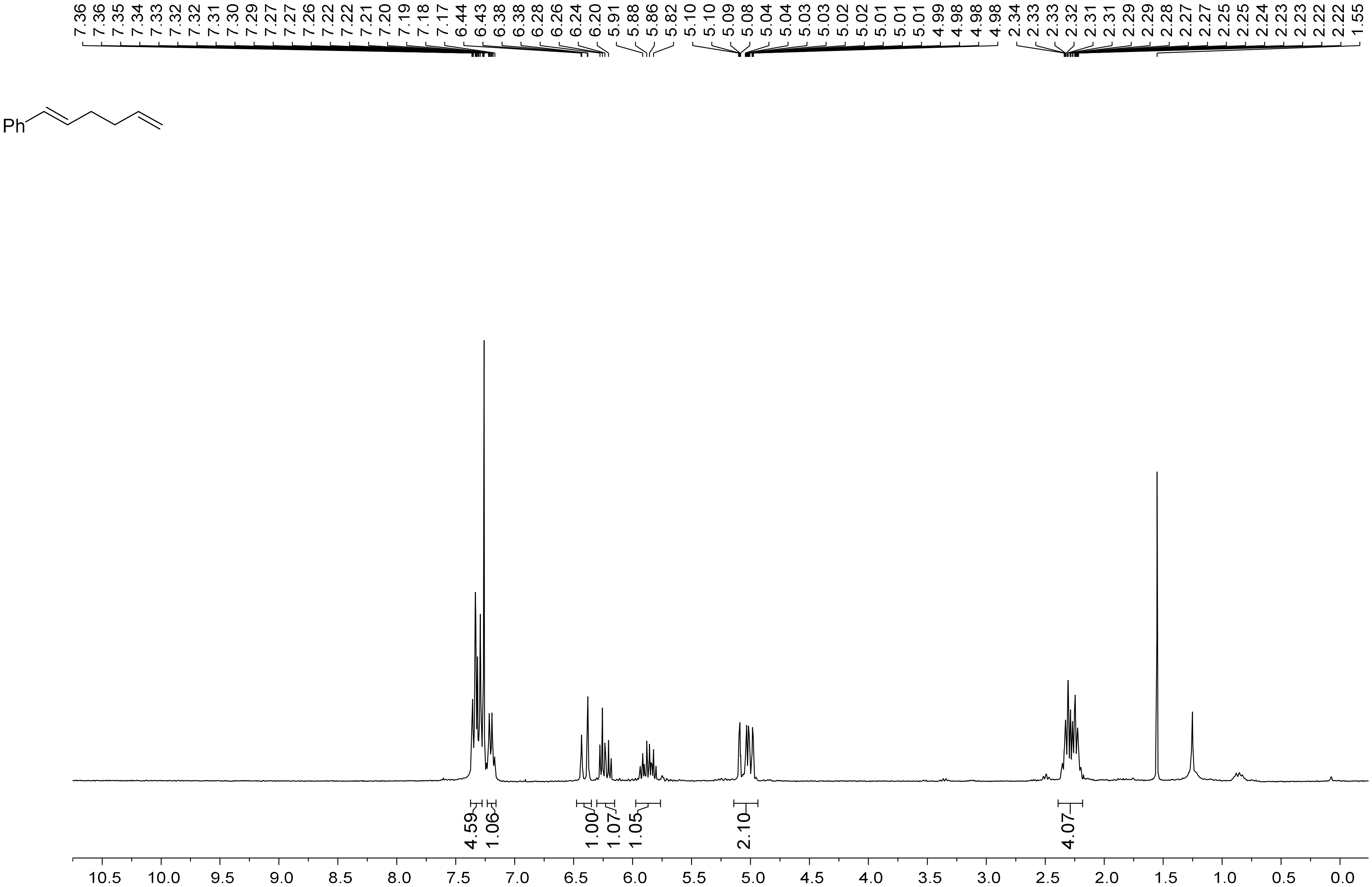

References

(1) Ang, H. T.; Rygus, J. P. G.; Hall, D. G. Two-component boronic acid catalysis for increased reactivity in challenging Friedel–Crafts alkylations with deactivated benzylic alcohols. *Org. Biomol. Chem.* **2019**, *17*, 6007-6014.

(2) Hsu, S.-F.; Plietker, B. Selective Transfer Hydrogenation and Hydrogenation of Ketones Using a Defined Monofunctional (P^N(Bn)^N(Bn)^P)–RuII Complex. *Chem. Eur. J.* **2014**, *20*, 4242-4245.

(3) Süsse, L.; Hermeke, J.; Oestreich, M. The Asymmetric Piers Hydrosilylation. *J. Am. Chem. Soc.* **2016**, *138*, 6940-6943.

(4) Barreiro, E.; Sanz-Vidal, A.; Tan, E.; Lau, S.-H.; Sheppard, T. D.; Díez-González, S. HBF4-Catalysed Nucleophilic Substitutions of Propargylic Alcohols. *Eur. J. Org. Chem.* **2015**, *2015*, 7544-7549.

(5) Bruneau-Voisine, A.; Wang, D.; Dorcet, V.; Roisnel, T.; Darcel, C.; Sortais, J.-B. Transfer Hydrogenation of Carbonyl Derivatives Catalyzed by an Inexpensive Phosphine-Free Manganese Precatalyst. *Org. Lett.* **2017**, *19*, 3656-3659.

(6) Nokami, T.; Yamane, Y.; Oshitani, S.; Kobayashi, J.-k.; Matsui, S.-i.; Nishihara, T.; Uno, H.; Hayase, S.; Itoh, T. The β-Silyl Effect on the Memory of Chirality in Friedel–Crafts Alkylation Using Chiral α-Aryl Alcohols. *Org. Lett.* **2015**, *17*, 3182-3185.

(7) Łowicki, D.; Bezłada, A.; Mlynarski, J. Asymmetric Hydrosilylation of Ketones Catalyzed by Zinc Acetate with Hindered Pybox Ligands. *Adv. Synth. Catal.* **2014**, *356*, 591-595.

(8) Schulte, B.; Fröhlich, R.; Studer, A. Atroposelective radical aryl migration reactions from sulfur to carbon. *Tetrahedron* **2008**, *64*, 11852-11859.

(9) Garcia, K. J.; Gilbert, M. M.; Weix, D. J. Nickel-Catalyzed Addition of Aryl Bromides to Aldehydes To Form Hindered Secondary Alcohols. *J. Am. Chem. Soc.* **2019**, *141*, 1823-1827.

(10) Yamashita, Y.; Suzuki, H.; Sato, I.; Hirata, T.; Kobayashi, S. Catalytic Direct-Type Addition Reactions of Alkylarenes with Imines and Alkenes. *Angew. Chem. Int. Ed.*

2018, *57*, 6896-6900.

(11) Hayashi, K.; Tanimoto, H.; Zhang, H.; Morimoto, T.; Nishiyama, Y.; Kakiuchi, K. Efficient Synthesis of α,β-Unsaturated Alkylimines Performed with Allyl Cations and Azides: Application to the Synthesis of an Ant Venom Alkaloid. *Org. Lett.* **2012**, *14*, 5728-5731.

(12) Zou, L.-H.; Priebbenow, D. L.; Wang, L.; Mottweiler, J.; Bolm, C. Copper-Catalyzed Synthesis of α-Thioaryl Carbonyl Compounds Through S□S and C□C Bond Cleavage. *Adv. Synth. Catal.* **2013**, *355*, 2558-2563.

(13) Rao, H. S. P.; Muthanna, N. Variations in the Blaise Reaction: Conceptually New Synthesis of 3-Amino Enones and 1,3-Diketones. *Eur. J. Org. Chem.* **2015**, *2015*, 1525-1532.

(14) Betterley, N. M.; Surawatanawong, P.; Prabpai, S.; Kongsaeree, P.; Kuhakarn, C.; Pohmakotr, M.; Reutrakul, V. Electrophilic Difluoro(phenylthio)methylation: Generation, Stability, and Reactivity of α-Fluorocarbocations. *Org. Lett.* **2013**, *15*, 5666-5669.

(15) Correia, C. A.; Li, C.-J. Catalytic alkylation of benzylic C–H bonds with 1,3-dicarbonyl compounds utilizing oxygen as terminal oxidant. *Tetrahedron Lett.* **2010**, *51*, 1172-1175.

(16) Noji, M.; Konno, Y.; Ishii, K. Metal Triflate-Catalyzed Cationic Benzylation and Allylation of 1,3-Dicarbonyl Compounds. *J. Org. Chem.* **2007**, *72*, 5161-5167.

(17) Liu, P. N.; Dang, L.; Wang, Q. W.; Zhao, S. L.; Xia, F.; Ren, Y. J.; Gong, X. Q.; Chen, J. Q. Perchloric Acid Catalyzed Homogeneous and Heterogeneous Addition of β-Dicarbonyl Compounds to Alcohols and Alkenes and Investigation of the Mechanism. *J. Org. Chem.* **2010**, *75*, 5017-5030.

(18) Chatterjee, P. N.; Roy, S. Alkylation of 1,3-dicarbonyl compounds with benzylic and propargylic alcohols using Ir–Sn bimetallic catalyst: synthesis of fully decorated furans and pyrroles. *Tetrahedron* **2011**, *67*, 4569-4577.

(19) Sanz, R.; Miguel, D.; Martínez, A.; Álvarez-Gutiérrez, J. M.; Rodríguez, F. Brønsted Acid-Catalyzed Benzylation of 1,3-Dicarbonyl Derivatives. *Org. Lett.* **2007**, *9*,

2027-2030.

(20) Cheng, H.-G.; Feng, B.; Chen, L.-Y.; Guo, W.; Yu, X.-Y.; Lu, L.-Q.; Chen, J.-R.; Xiao, W.-J. Rational design of sulfoxide–phosphine ligands for Pd-catalyzed enantioselective allylic alkylation reactions. *Chem. Commun.* **2014**, *50*, 2873-2875.

(21) Gao, W.-C.; Jiang, S.; Wang, R.-L.; Zhang, C. Iodine-mediated intramolecular amination of ketones: the synthesis of 2-acylindoles and 2-acylindolines by tuning N-protecting groups. *Chem. Commun.* **2013**, *49*, 4890-4892.

(22) Zhu, Q.; Gentry, E. C.; Knowles, R. R. Catalytic Carbocation Generation Enabled by the Mesolytic Cleavage of Alkoxyamine Radical Cations. *Angew. Chem. Int. Ed.* **2016**, *55*, 9969-9973.

(23) Umeda, R.; Jikyo, T.; Toda, K.; Osaka, I.; Nishiyama, Y. Rhenium complex-catalyzed carbon-carbon formation of alcohols and organosilicon compounds. *Tetrahedron Lett.* **2018**, *59*, 1121-1124.

(24) Saito, T.; Nishimoto, Y.; Yasuda, M.; Baba, A. Direct Coupling Reaction between Alcohols and Silyl Compounds: Enhancement of Lewis Acidity of Me3SiBr Using InCl3. *J. Org. Chem.* **2006**, *71*, 8516-8522.

(25) Bohan, P. T.; Toste, F. D. Well-Defined Chiral Gold(III) Complex Catalyzed Direct Enantioconvergent Kinetic Resolution of 1,5-Enynes. *J. Am. Chem. Soc.* **2017**, *139*, 11016-11019.

(26) Durand, A. C.; Brahmi, L.; Lahrech, M.; Hacini, S.; Santelli, M. Preparation of 4‐Arylcyclopentenes by Sequential Diallylation of Arylaldehydes and Ring‐Closing Metathesis. *Synth. Commun.* **2005**, *35*, 1825-1833.

(27) Mahoney, S. J.; Lou, T.; Bondarenko, G.; Fillion, E. Carbon-Based Leaving Group in Substitution Reactions: Functionalization of sp3-Hybridized Quaternary and Tertiary Benzylic Carbon Centers. *Org. Lett.* **2012**, *14*, 3474-3477.

(28) Cao, H.; Jiang, H.; Feng, H.; Kwan, J. M. C.; Liu, X.; Wu, J. Photo-induced Decarboxylative Heck-Type Coupling of Unactivated Aliphatic Acids and Terminal Alkenes in the Absence of Sacrificial Hydrogen Acceptors. *J. Am. Chem. Soc.* **2018**, *140*, 16360-

16367.
